# From Risk Markers to Preventive Action: A Proposed Semi-Quantitative Clinical Actionability Framework for Cardiometabolic Precision Prevention in Primary Care—A Narrative Review

**DOI:** 10.3390/jcm15218706

**Published:** 2026-11-07

**Authors:** Antoneta Dacia Petroaie, Elena Teona Cosovanu, Eric Oliviu Cosovanu, Otilia Novac, Ana Maria Slanina, Elena Popa, Agnes Iacinta Bacusca, Mihaela Manole, Monica Iuliana Ungureanu, Carmen Liliana Barbacariu, Luiza Simona Pohaci Antonesei, Manuela Stefan, Elena Adorata Coman

**Affiliations:** “Grigore T. Popa” University of Medicine and Pharmacy, 700115 Iasi, Romania; antoneta.petroaie@umfiasi.ro (A.D.P.); otilia.novac@umfiasi.ro (O.N.); ana.slanina@umfiasi.ro (A.M.S.); elena.popa@umfiasi.ro (E.P.); agnes.bacusca@umfiasi.ro (A.I.B.); mihaela.manole@umfiasi.ro (M.M.); monica.ungureanu@umfiasi.ro (M.I.U.); carmen.barbacariu@umfiasi.ro (C.L.B.); pohaci.antonesei@umfiasi.ro (L.S.P.A.); stefan.manuela@umfiasi.ro (M.S.); elena.coman@umfiasi.ro (E.A.C.)

**Keywords:** cardiometabolic risk, primary prevention, primary health care, risk assessment, biomarkers, clinical utility, precision medicine, lipoprotein(a), coronary artery calcium, albuminuria

## Abstract

Cardiometabolic prevention in primary care draws on a growing number of risk markers, yet a marker’s capacity to predict risk is often conflated with its capacity to change management. This narrative review proposes a semi-quantitative clinical actionability framework that takes the marker–context–action use case as its unit of appraisal and assigns ordinal scores (0–3) to seven domains: measurement readiness, prognostic validity, decision-relevant information, therapeutic linkage, clinical-utility evidence, harms and burden, and transportability and equity. Gating rules prevent prognostic strength alone from determining the tier. Applied by the authors to 19 use cases, the framework provisionally assigned five to Tier A (routine actionability): low-density lipoprotein cholesterol, blood pressure, type 2 diabetes detection, kidney function with albuminuria in diabetes or hypertension, and familial hypercholesterolaemia case-finding. Nine were assigned to Tier B (selective actionability), including the coronary artery calcium, apolipoprotein B, lipoprotein(a), natriuretic peptide and ankle–brachial index use cases, three to Tier C and two to Tier D. Most assignments depended on one-point differences in therapeutic linkage or clinical-utility scores. Tiers describe evaluated use cases, not intrinsic marker properties; the framework has not been validated. It implies that closing implementation gaps in Tier A use cases should generally precede the introduction of newer markers.

## 1. Introduction

Atherosclerotic cardiovascular disease (ASCVD), heart failure (HF), type 2 diabetes (T2D) and chronic kidney disease (CKD) share risk factors, pathophysiological pathways and therapies and are now framed as a single cardiovascular–kidney–metabolic (CKM) continuum calling for staged prevention across the life course [1], an approach translated by a 2026 multisociety guideline into recommendations for prevention, detection and management [2]. Metabolic dysfunction-associated steatotic liver disease (MASLD), defined by hepatic steatosis with at least one cardiometabolic risk factor in the absence of harmful alcohol intake [3], is associated with a higher risk of cardiovascular events [4]. Cardiovascular diseases remain a leading cause of death and disability worldwide [5], and in 2022 an estimated 828 million adults had diabetes, with 445 million (59%) of those aged 30 years or older with diabetes being untreated [6]. Most people who will develop these conditions spend years beforehand as asymptomatic adults in primary care, where preventive decisions are made, deferred or missed.

The number of available markers and prediction models has grown faster than the evidence on how to use them: a systematic review identified 363 prediction models for cardiovascular disease in the general population, only 36% of which had been externally validated [7]. The 2025 European dyslipidaemia update lists elevated lipoprotein(a) [Lp(a)], persistently elevated high-sensitivity C-reactive protein (hs-CRP) and subclinical atherosclerosis on imaging among its risk modifiers [8], and the 2026 United States dyslipidaemia guideline recommends measuring Lp(a) at least once in adulthood, adopts the Predicting Risk of cardiovascular disease EVENTs (PREVENT) equations and supports selective coronary artery calcium (CAC) scoring [9]. The core PREVENT equations include the estimated glomerular filtration rate (eGFR), whereas the urinary albumin-to-creatinine ratio (UACR), glycated haemoglobin (HbA1c) and a social deprivation index are optional predictors [10]. Delivery lags behind: primary prevention in people at high cardiovascular risk was poorly developed in 16 European countries [11], and albuminuria testing in hypertension or diabetes was infrequent across a global consortium of health systems [12].

In cardiometabolic disease, the most consequential form of precision prevention may lie in deciding which of the many available markers to measure and act upon. European guidance estimates 10-year cardiovascular risk with the Systematic Coronary Risk Evaluation 2 (SCORE2) algorithms, calibrated to four risk regions [13], whereas United States guidance has moved to the PREVENT equations [9], which estimate total cardiovascular disease, including HF, in adults aged 30–79 years [10]. Both guideline families add markers to refine these estimates, but neither provides a common metric for comparing how much each marker contributes to decisions, as distinct from prediction.

Established frameworks describe how a new marker should be evaluated [14,15], and a 2026 American Heart Association scientific statement has updated the criteria for assessing predictive and clinical utility, including actionability, generalisability, cost and implications for health equity [16]. These frameworks define the dimensions of predictive and clinical utility but do not operationalise them into a cross-marker semi-quantitative appraisal centred on a defined use of the marker. Two questions are therefore often conflated: whether a marker predicts risk and whether measuring it leads to decisions that improve outcomes. General health checks, which offer many tests to adults unselected for disease or risk factors, had little or no effect on total or cardiovascular mortality in a Cochrane review of randomised trials [17], whereas a marker with modest predictive value can change management when it identifies people who benefit from an effective therapy [18]. Semi-quantitative instruments for grading actionability have been developed in genomic medicine [19]; the present framework adapts this approach to cardiometabolic prevention by centring appraisal on a defined marker–context–action use case.

A narrative synthesis suits this problem because the relevant evidence, from Mendelian randomisation studies, prospective cohorts, individual-participant meta-analyses, randomised trials of treatments and of testing strategies, implementation studies and guidelines, cannot be pooled. The review has four objectives: to clarify the concepts that separate risk markers from actionable markers; to propose a semi-quantitative clinical actionability framework adapted to primary care; to apply it to 19 marker–context–action use cases across five marker classes; and to derive implications for primary care pathways and research priorities. The framework is offered as a provisional, structured basis for appraisal and comparison; it has not been validated and is not a clinical guideline.

## 2. Approach to Evidence Identification and Synthesis

This narrative review was planned and reported with reference to the Scale for the Assessment of Narrative Review Articles (SANRA) [20]. The literature was identified through iterative, concept-driven searching of PubMed/MEDLINE, supplemented by backward and forward citation tracking from landmark trials, individual-participant meta-analyses and current guidelines. Searches addressed cardiometabolic outcomes, the markers appraised in Section 5 and evaluative concepts such as incremental value, reclassification, clinical utility, testing-strategy trials, harms and equity. Priority was given to randomised trials of marker-directed or marker-guided care and of testing strategies, individual-participant meta-analyses and large prospective cohorts, Mendelian randomisation studies, and current guidelines and consensus documents; older seminal work was retained where it established a concept. Only peer-reviewed journal publications were cited; preprints, conference abstracts and trial results announced without peer-reviewed publication were not used to score any domain (one such announcement is mentioned for context in Section 5.3), and studies conducted outside the scored population or decision were used only as indirect evidence, as specified by the domain anchors (Table 1). For each use case, evidence that challenged the prevailing interpretation, such as null outcome trials or failed replication of incremental value, was specifically sought; conflicting evidence is reported for the use case concerned (Section 5 and Table S1) and handled by the reading rules of the anchors, not by selecting the more favourable result. Evidence was synthesised thematically and appraised qualitatively for design, size, directness, consistency and transportability. Because the searches were iterative and concept-driven rather than protocol-based, the review does not claim exhaustive coverage.

Use cases were selected to illustrate the framework across the categories of markers that are measured, or proposed for measurement, in cardiometabolic prevention: established causal risk factors, markers of cardiometabolic organ involvement, circulating risk enhancers, subclinical atherosclerosis imaging, and genomic, multi-omic and life-course markers. Within each category, markers were included when they are recommended or discussed in current European or United States guidance on prevention [2,8,9], are available or under active evaluation for use in primary care, and have been examined in outcome-based studies of prognosis or clinical utility. Each marker was scored for the context and decision in which the strongest evidence exists, so the resulting tiers describe these selected use cases, not the markers in general (Section 4.1); when one test informs two decisions supported by different evidence, as glycaemic testing does for T2D and for prediabetes, the decisions were scored as separate use cases. The set is illustrative and does not claim to include every available marker. Comorbid conditions that are diagnosed and managed in their own right, such as chronic obstructive pulmonary disease (COPD) and obstructive sleep apnoea (OSA), were not scored as use cases; their relation to the framework is considered in Section 8.

Conventional clinical characteristics that are recorded in every assessment were not scored as separate use cases when their role is to define the context of a decision or to enter risk equations. Adiposity illustrates this choice. Body-mass index and waist circumference are recommended for CKM assessment, define an early CKM stage and are targets of lifestyle and pharmacological treatment [1,2], and obesity is listed among the European risk modifiers [8]. As sources of incremental risk information, however, they add little: in 58 prospective cohorts, adding body-mass index, waist circumference or waist-to-hip ratio to conventional risk factors did not meaningfully improve discrimination or reclassification [21]. Adiposity therefore enters the framework as a variable that defines the context of several use cases (Table 2) and as a treatment target within CKM care, not as a separately scored marker. Social deprivation is treated in the same way (Section 5.5).

Domain scores represent ordinal structured judgements made by the authors against the predefined anchors in Table 1 (Section 4), several of which were refined during revision of the manuscript, based on the evidence summarised for each use case; the scoring procedure, the reassessment performed during revision and their limitations are described in Section 4.5. The rationale for every score, including the route by which decision-relevant information was credited and the two subcomponent ratings of transportability and equity, is documented in Table S1; the quantitative results of the studies that inform the scores are summarised in Table S2; a self-assessment against the SANRA items is provided in Table S3; the sensitivity of the tier assignments to changes in scores, thresholds, weights and rules is reported in Table S4A,B; and the construct definition of each domain, with the rules that prevent double counting, is given in Table S5. Together, these tables allow a reader to trace each score to its evidentiary basis.

## 3. Conceptual Foundations: From Association to Action

### 3.1. Risk Markers, Risk Factors, Targets and Actionable Markers

Four related terms are often used interchangeably, and much of the confusion surrounding new cardiometabolic markers stems from this. A risk marker is any measurable characteristic that is statistically associated with future disease. A risk factor is a marker that also lies on the causal pathway to that disease. A therapeutic target is a causal factor whose deliberate modification has been shown to reduce clinical events. An actionable marker, as used here, is a marker whose measurement in a defined population changes a clinical decision in a way that is expected to improve outcomes, net of harms and burden.

These categories overlap only partially. Low-density lipoprotein cholesterol (LDL-C) meets all four definitions: evidence from more than 200 cohort studies, Mendelian randomisation studies and randomised trials including more than 2 million participants shows a consistent, dose-dependent, log-linear relation between exposure to low-density lipoproteins and ASCVD that increases with the duration of exposure [22], and lowering LDL-C reduces events [23]. C-reactive protein (CRP) is a reproducible risk marker, but genetically raised CRP was not associated with coronary heart disease (risk ratio 1.00 per 1 standard deviation; 95% confidence interval [CI] 0.90–1.13), in contrast with a risk ratio of 1.33 per standard deviation for measured CRP [24]. Its measurement can nevertheless change decisions by identifying people who benefit from statin therapy [18]. Lp(a) is supported as a causal risk factor by genetic evidence, with a hazard ratio for myocardial infarction of 1.22 per doubling of genetically determined Lp(a) [25], yet it has not been established as a therapeutic target. CAC is not a risk factor at all but a direct measure of subclinical coronary atherosclerosis, which explains both its prognostic strength [26] and why its value lies in triage and not in being lowered.

Two routes to actionability follow from these distinctions. Along the target route, a marker is acted upon because lowering it lowers risk; this requires causal evidence and trials of marker-directed therapy. Along the triage route, a marker is acted upon because it identifies people in whom an intervention of proven efficacy yields a larger (or smaller) absolute benefit than risk equations alone would suggest; this requires decision-relevant incremental information and, ideally, trials in marker-defined populations. Most emerging cardiometabolic markers can at present claim only the triage route.

### 3.2. Lessons from Established Test-Evaluation Frameworks

The American Heart Association criteria for novel cardiovascular risk markers set out the sequence that later frameworks have followed: initial proof of concept, prospective validation in independent populations, documentation of incremental information over standard markers, assessment of effects on patient management and outcomes, and cost-effectiveness [14]. They parallel the hierarchical model of diagnostic efficacy, which progresses from technical performance and diagnostic accuracy through diagnostic thinking and therapeutic efficacy to patient and societal outcomes [15]. The 2026 scientific statement that updates the American Heart Association criteria organises the evaluation of models, biomarkers and tools around general considerations in risk prediction, statistical assessment of predictive utility and appraisal of clinical utility, including actionability, scalability and generalisability, and it adds reproducibility, cost–value considerations and implications for health equity [16]. Three lessons from this tradition shape the present framework. First, association and even improved discrimination do not establish clinical utility; decision-analytic measures such as net benefit, which weigh the consequences of acting on a result across clinically relevant thresholds, are more informative than changes in the C-statistic [27]. Second, most marker studies stop at the stage of incremental prognostic value, and few reach randomised evaluation of marker-guided care. Third, these frameworks specify which evidence is needed but do not grade how much has accumulated on a common scale, which makes comparisons across markers difficult.

The first lesson deserves emphasis because it explains many apparently contradictory findings. A marker can add little to the C-statistic across a whole population yet change decisions for a meaningful proportion of people close to a treatment threshold, and the reverse is also possible. Whether a result is informative therefore depends on where the decision threshold lies and on how many people are near it, which is why the present framework assesses incremental information in the decision zone instead of across the entire risk distribution [14,27].§

Genomic medicine has addressed the last problem with an explicitly semi-quantitative instrument. A metric developed for incidental and secondary findings from genome-scale sequencing scores five elements of actionability, namely the severity and likelihood of the disease outcome, the efficacy and burden of the intervention and the knowledge base, each from 0 to 3, giving a total of 0 to 15 [19]. It shows that actionability can be graded transparently and revised as evidence matures. It was not, however, designed for population-level prevention in primary care, where the harms and costs of testing large numbers of asymptomatic people, the feasibility of measurement and follow-up, and the risk of widening inequities weigh as heavily as the strength of the marker–outcome association.

### 3.3. From Risk-Based to Benefit-Based Prevention

Rose’s observation that a large number of people at small risk may give rise to more cases than a small number at high risk remains central to prevention [28]. Contemporary guidelines resolve this tension pragmatically, combining population-wide measures with treatment thresholds based on estimated absolute risk [9,29]. Risk-based thresholds approximate benefit because a treatment with a constant relative effect produces larger absolute gains at higher baseline risk. The approximation has limits. In the Cholesterol Treatment Trialists’ meta-analysis, the relative reduction in major vascular events per 1 mmol/L LDL-C lowering was, if anything, greater at the lowest levels of baseline risk, yet the absolute gain in people with a 5-year risk below 10% was about 11 fewer major vascular events per 1000 treated over 5 years for each 1 mmol/L reduction [23]. An approach based on the estimated absolute risk reduction from statin therapy identified lower-risk individuals whose expected benefit was equal to or greater than that of higher-risk individuals [30]. A 2026 scientific statement of the American Heart Association and the American College of Cardiology on CKM syndrome describes combining predicted risk from the PREVENT equations with the expected relative risk reduction of a therapy to estimate absolute benefit, and it places cardiac biomarkers and imaging within a stepwise process of risk calculation, personalisation and reclassification [31]. For the purpose of this review, precision prevention is defined accordingly: the use of markers to match the type, timing and intensity of preventive interventions to expected individual benefit. A marker is actionable to the extent that it changes this expected benefit, or the choice of intervention, for the person tested.

### 3.4. Measurement Properties That Matter in Primary Care

Measurement characteristics that receive little attention in prognostic studies often determine whether a marker can be acted upon in practice. Biological variability matters: UACR varies within individuals, so CKD guidance requires an elevated value to be confirmed on a repeat sample [32], and hs-CRP rises transiently with intercurrent illness, which is why the European update specifies persistently elevated values [8]. Stability also matters but in the opposite direction. Lp(a) concentration is largely genetically determined, and consensus statements therefore recommend measuring it at least once in adults [33]. The setting of measurement can dominate its information content. In a cohort of 59,124 patients with a median follow-up of 9.7 years, 24 h ambulatory systolic blood pressure (BP) was more strongly associated with all-cause death than clinic systolic BP (hazard ratio [HR] per standard deviation 1.41 versus 1.18 in the upper four fifths of each distribution), and after mutual adjustment the association of clinic BP was almost abolished (HR 1.04; 95% CI 1.00–1.09) [34]. Units and assays create further friction: Lp(a) is reported in mass or molar units by assays that are not yet fully standardised, and the European Atherosclerosis Society panel does not recommend a standard factor for converting between them, suggesting only an approximate range of 2–2.5 nmol/L per mg/dL [33]. These properties are captured in the framework as measurement readiness and inform the timing of repeat testing.

## 4. A Proposed Semi-Quantitative Clinical Actionability Framework

### 4.1. Unit of Appraisal: The Marker–Context–Action Triad

Actionability is not a property of a marker in isolation. The same measurement may be decisive in one population and uninformative in another, and the same result may be actionable for one decision but not another. In people with diabetes or hypertension, UACR identifies CKD for which therapies reduce kidney and cardiovascular events [35,36]; no comparable evidence supports routine testing in unselected young adults. Natriuretic peptides have been evaluated as a guide to intensified prevention in people with T2D or other cardiovascular risk factors [37,38] but not in the general population. The framework therefore scores use cases defined by three elements: the marker, the population in which it is measured and the decision it is intended to inform. The tiers reported here therefore describe the evaluated use cases, each defined for the context and decision with the strongest evidence (Section 2), and not intrinsic properties of the markers.

### 4.2. Seven Domains

**Figure 1:**
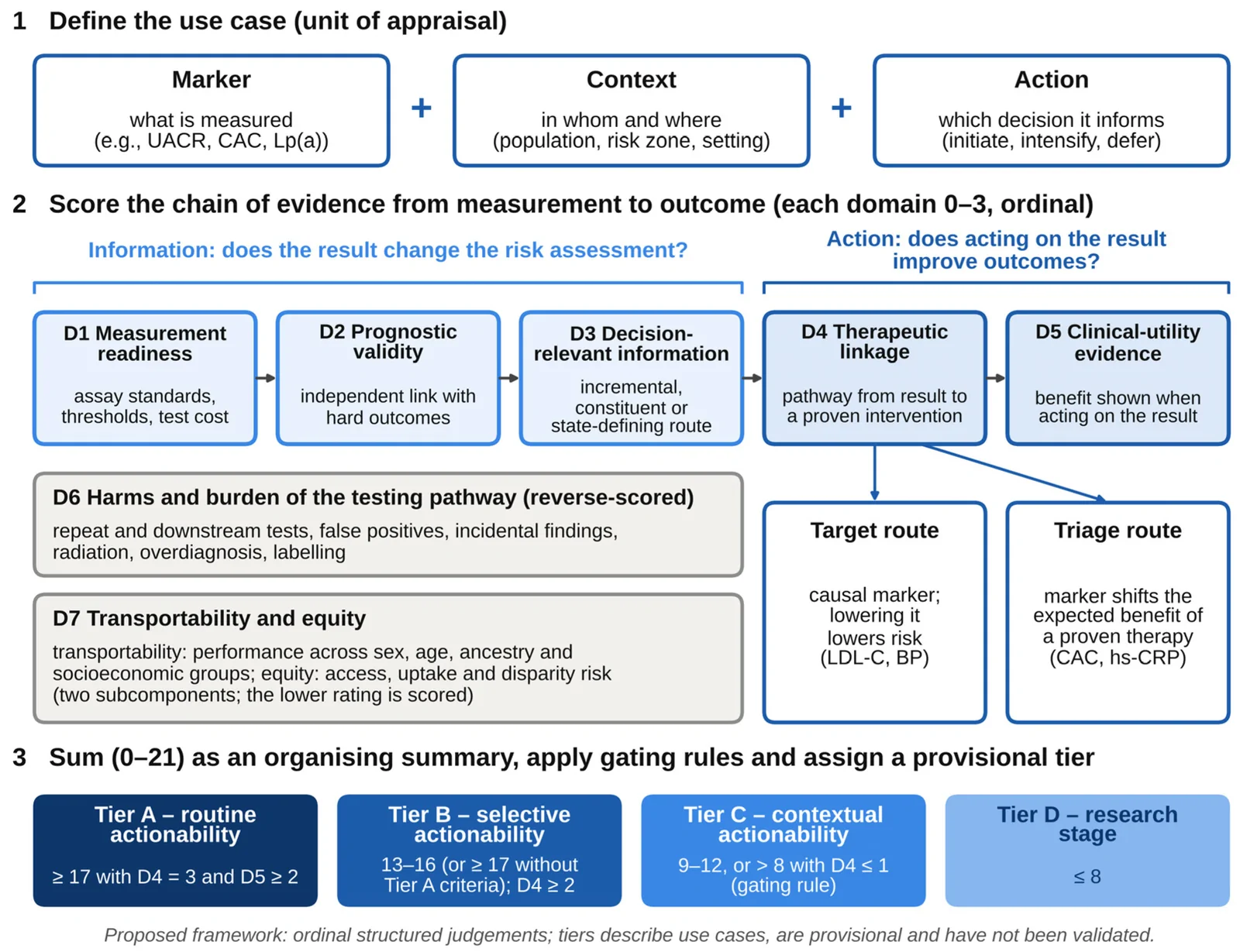
Structure of the proposed semi-quantitative clinical actionability framework. Each use case is defined by a marker, the context in which it is measured and the decision it informs (1). Seven domains are each given an ordinal score from 0 to 3 (2): D1–D3 describe the information provided by the result, with decision-relevant information (D3) credited through an incremental, constituent or state-defining route, and D4 and D5 describe the consequences of acting on it, through either the target route (lowering a causal marker lowers risk) or the triage route (the marker shifts the expected benefit of a proven therapy); D6 (reverse-scored) captures the harms and burden of the testing pathway, and D7 transportability and equity, rated as two subcomponents of which the lower is scored. The total (0–21), an organising summary rather than a measurement, is combined with two gating rules (D4 ≤ 1 caps a use case at Tier C; Tier A requires D4 = 3 and D5 ≥ 2) to assign one of four provisional tiers (3). Original schematic developed by the authors to depict the proposed framework. BP, blood pressure; CAC, coronary artery calcium; hs-CRP, high-sensitivity C-reactive protein; LDL-C, low-density lipoprotein cholesterol; Lp(a), lipoprotein(a); UACR, urinary albumin-to-creatinine ratio.

Each use case is scored on seven domains from 0 to 3 (total 0–21) using the predefined anchors in Table 1, of which those for D1, D3, D6 and D7 were refined during revision (Section 4.5). The scores are ordinal structured judgements: each level denotes a category of evidence defined by its anchor, not an interval-scale measurement, a probability, an effect size or a validated estimate of clinical utility, and the score assigned is the highest level whose anchor is met by the evidence cited for that domain in the scored context. The domains follow the chain of evidence that links a measurement to a better outcome (Figure 1). D1 (measurement readiness) captures analytical and practical readiness in primary care: analytical validity, standardisation, established thresholds, availability, turnaround and the cost of the test itself. D2 (prognostic validity) captures the strength, consistency and independence of the association with hard cardiometabolic outcomes, defined here as cardiovascular events, kidney failure, severe liver disease and death. D3 (decision-relevant information) asks whether the result informs the decision at hand. Three routes are distinguished, and those credited are recorded for every use case (Table S1): incremental information beyond baseline assessment, graded by the extent of reclassification near the decision threshold, including de-risking (route I); being a core constituent of guideline-recommended risk equations, so that the information is already incorporated in baseline risk estimation (route C); or direct identification of a clinically recognised high-risk state for which guidelines direct management irrespective of calculated risk (route S). The routes are not statistically equivalent: route I is supported by measures of discrimination and reclassification, whereas routes C and S reflect the role that guidelines assign to the marker in defining the baseline estimate or the high-risk state. They are combined in one domain because each makes the result necessary for, or determinative of, the decision; routes C and S are credited with the maximum score, and the consequences of capping them are examined in Section 5.6.

D4 (therapeutic linkage) assesses whether a credible pathway leads from the result to an intervention of established efficacy and how directly the result triggers or modifies it. The pathway is direct (score 3) when the marker is an established treatment target, or defines a condition, for which an intervention reduces hard outcomes; it is indirect (score 2) when the result shifts the expected absolute benefit of an intervention of proven efficacy (triage route) or when the marker is a target whose modification has improved surrogate outcomes only. D5 (clinical-utility evidence) grades direct evidence that care informed by the marker, delivered to a population defined by the marker or directed at the condition it defines, improves meaningful outcomes. The two domains ask different questions of the evidence. D4 records whether and how directly a result leads to an effective intervention; D5 records how directly the benefit of acting on the result has been demonstrated in the scored context. A treatment trial therefore establishes the pathway in D4 but contributes to D5 only if it enrolled people defined by the marker or tested therapy directed at the marker or its condition, so that no trial is counted twice for the same property: in D4 it shows that the linked intervention is efficacious, and in D5 that acting on the result benefits the people it selects. For target-route markers such as LDL-C and BP, treatment is directed at the measured value, so the same trials legitimately inform both domains, and the two scores are correlated by design; the effect of counting D4 and D5 only once is examined in Section 5.6. Trials of testing strategies are graded in D5; they also raise D4 only when they show that treating the condition identified by the marker reduces hard outcomes. Three reading rules keep D5 consistent across use cases: conflicting randomised evidence at a given level does not support that level; consistent evidence of no benefit scores 0; and when a randomised evaluation of the testing strategy itself did not improve hard outcomes, D5 cannot exceed 2, even if treatment of the condition detected is effective. Intermediate outcomes count as validated when they are measured risk factors or disease states with an established relation to hard outcomes, such as LDL-C, BP or incident T2D; calculated risk scores are predicted-risk constructs and are not counted as intermediate outcomes.

D6 (harms and burden) is reverse-scored and captures the harms and burdens that the testing pathway imposes on the person tested: the index measurement, confirmatory and downstream investigations, invasive follow-up, incidental findings, radiation, false-positive results, overdiagnosis and labelling. The cost and availability of the test are scored only in D1, and inequity of access only in D7, so that the same cost or access problem is not penalised in more than one domain. Adverse effects of the treatments that follow are not scored in D6 because they are already reflected in the net outcome evidence graded in D4 and D5; this convention is applied to every use case. D7 (transportability and equity) contains two distinct subcomponents, which are rated separately on the same anchors: transportability (T), meaning that measurement, thresholds and prognostic performance hold across sex, age, ancestry and socioeconomic groups and across settings, and equity (E), meaning equitable access, uptake and follow-through and a low risk that implementation widens disparities. The D7 score is the lower of the two ratings, so that a score of 3 requires affirmative evidence of both; the absence of an identified limitation is not sufficient. Scoring the two subcomponents as separate domains was considered but not adopted in this first-generation framework because direct evidence on equity is sparse for most use cases and an eighth domain would add a further unsupported quantitative assumption to the total. Both ratings are reported for every use case (Table S1), and whether separate scoring is preferable should be tested in validation studies.

The domains are deliberately heterogeneous. D1 to D3 describe the information chain, from a reliable measurement to decision-relevant risk information; D4 and D5 describe consequences for care; and D6 and D7 describe the burden and fairness of testing in real practice. A use case can therefore score well overall for different reasons, and the profile across domains is as informative as the total. For each domain, Table S5 specifies what the domain measures, which evidence belongs to it, which evidence is scored elsewhere and the rule that prevents double counting; these construct definitions were made explicit during revision of the manuscript and were applied in a reassessment of every score (Section 4.5 and Section 5).

### 4.3. Scoring Algorithm, Tiers and Gating Rules

The seven domain scores are converted into a tier by a fixed algorithm, applied identically to every use case (Figure 1 and Table 1). The scores are summed with equal weight (total 0–21), and the total is read together with two gating rules: a use case with a total of 8 or less is assigned to Tier D (research stage); otherwise, it is assigned to Tier A (routine actionability) if its total is at least 17, its therapeutic linkage score is 3 and its clinical-utility score is at least 2; to Tier C (contextual actionability) if its therapeutic linkage score is 1 or less, however high its total; to Tier B (selective actionability) if its total is at least 13; and to Tier C otherwise. The total is a transparent organising summary of the domain profile, not a measurement: a difference of one point carries no precise quantitative clinical meaning, and use cases with the same total can have different profiles. In Tier B, the test is performed, or its result acted on, when it could plausibly change a specific decision; in Tier C, a result may contribute to an individual decision in a specific context but does not by itself justify testing or a change in treatment. The labels describe the degree of actionability supported by current evidence within the proposed framework; they are provisional outputs, not treatment recommendations, and the framework has not undergone formal guideline development, consensus or independent validation. The first gating rule encodes the central premise of the framework: prediction without a therapeutic consequence cannot justify routine testing. The second prevents a use case from reaching the highest tier on prognostic and mechanistic grounds alone. Tiers describe what can be done with a result, not how often information is collected: a history item or a one-time laboratory measurement with low burden may be obtained broadly, and the tier of its use case then governs the action taken on the result.

Equal weighting is a design choice, not an empirical finding. No validated basis currently exists for weighting the domains differently, and unequal weights would introduce a further unsupported quantitative assumption; equal weights keep the summation transparent at this developmental stage and treat each domain as a distinct component of actionability. The asymmetry that matters most, that strong prediction should not compensate for weak therapeutic linkage or clinical-utility evidence, is handled by the gating rules rather than by weights: a therapeutic linkage score of 1 or less caps a use case at Tier C, and Tier A requires a clinical-utility score of at least 2. Equal weighting has not been shown to be optimal, and alternative weights can change classifications (Section 5.6). The tier thresholds are heuristic. They were set so that benchmark risk factors with undisputed actionability fall in Tier A and markers without an associated intervention cannot; beyond this, the cut-points have no empirical derivation, and they assume that ordinal levels can be summed and that the benchmark use cases represent routine actionability. This procedure is calibration, not validation. Because the benchmark use cases informed the thresholds, their assignment to Tier A follows from the calibration and cannot be cited as evidence that the thresholds are valid; the assignments of the other use cases are not independent tests either because the same authors set the thresholds and assigned the scores. Evaluation of the framework therefore requires evidence independent of the calibration: reliability, shown by agreement between assessors not involved in its development, and validity, shown by comparison with the judgements of independent expert panels and, ultimately, by evidence that framework-informed decisions improve care (Section 8). The thresholds were not changed during revision; instead, the dependence of each assignment on them was quantified (Section 5.6).

### 4.4. A Worked Example: Coronary Artery Calcium

Scoring CAC in adults aged 40–75 years at borderline or intermediate risk illustrates how the domains interact. Measurement is standardised but requires computed tomography referral, with availability and test cost that vary between health systems (D1 = 2). Prognostic validity is strong and graded across ethnic groups (D2 = 3) [26], and among intermediate-risk participants of the Multi-Ethnic Study of Atherosclerosis (MESA), CAC improved discrimination and reclassification more than the other novel markers compared, with de-risking when the score is zero (D3 = 3, route I) [39,40]. Its therapeutic consequence runs entirely through the triage route, by favouring or deferring statin therapy (D4 = 2). Randomised evidence shows improvements in risk-factor profiles without increased downstream testing [41], but a population screening programme that included CAC did not significantly reduce all-cause mortality (D5 = 2) [42]. Radiation exposure and incidental findings lower D6 to 1, and D7 scores 2: prediction is consistent across four ethnic groups (transportability rated 3), but equitable access to scanning has not been shown (equity rated 2). The total of 15 places the evaluated CAC use case in Tier B: within the framework, a test to consider when its result can change a statin decision, not one to apply to everyone.

### 4.5. Scoring Procedure and Interpretation

The scores are structured author judgements, not measurements. Their purpose is to make the reasoning behind an appraisal explicit and open to challenge, domain by domain, not to replace guideline processes. The scores for the 19 use cases were originally assigned by the authors against the anchors, and the evidence and rationale for every score are recorded in Table S1. The use cases were not scored independently by two or more assessors, and no formal procedure for resolving disagreement was used; inter-rater agreement was therefore not measured and cannot be reported (Section 7). During revision of the manuscript, after the anchors for D1, D3, D6 and D7 had been refined and the construct definitions in Table S5 made explicit, every score was re-assessed by asking whether the cited evidence met the anchor of the assigned level; this reassessment was made with knowledge of the original scores and was neither blinded nor independent, and six scores were changed (Section 5). Because the anchors, routes, subcomponents and rules against double counting are specified explicitly (Table 1 and Table S5), each score can be inspected and the rules re-applied by others; explicit rules do not, however, show that independent assessors would reach the same scores, and the reliability of the scoring remains to be assessed (Section 8). Scores are also time-dependent: a single adequately powered outcome trial may move a use case between tiers, and the evidence expected to do so is identified in Section 8. Compared with checklist frameworks, including the updated American Heart Association criteria [14,15,16], the proposed framework grades the evidence accumulated at each step on a common ordinal scale for a defined use; compared with genomic actionability metrics [19], it adds domains for the burden of testing, primary care feasibility and equity, and it introduces gating rules that prevent strong prediction from masking weak therapeutic linkage.

### 4.6. Scoring a New Use Case

The framework can be applied to markers not considered here, and scoring is most transparent when it follows a fixed order. The triad is specified first, together with the treatment threshold that defines the decision zone. The claimed route to action is identified next because the target route requires causal evidence and trials of marker-directed therapy, whereas the triage route requires evidence that the result shifts expected benefit near that threshold. The strongest evidence for each domain is then located, including null trials and failed replication; each item of evidence is assigned to the domain whose construct it informs (Table S5), the D3 route and the two D7 subcomponents are recorded, and harms are assessed across the whole testing pathway. Finally, the gating rules are applied, the tier is read from the total, and the domain most likely to change the tier is recorded as its migration trigger. The scores in this review follow this sequence, except that the assignment of evidence to domains according to Table S5 and the recording of the D3 route and of the D7 subcomponents were introduced during revision and applied in the reassessment of all scores (Section 4.5). The scores reflect the judgement of the authors, and independent rescoring and formal validation of the framework are priorities for future work (Section 8).

## 5. Applying the Framework Across Marker Classes

**Figure 2:**
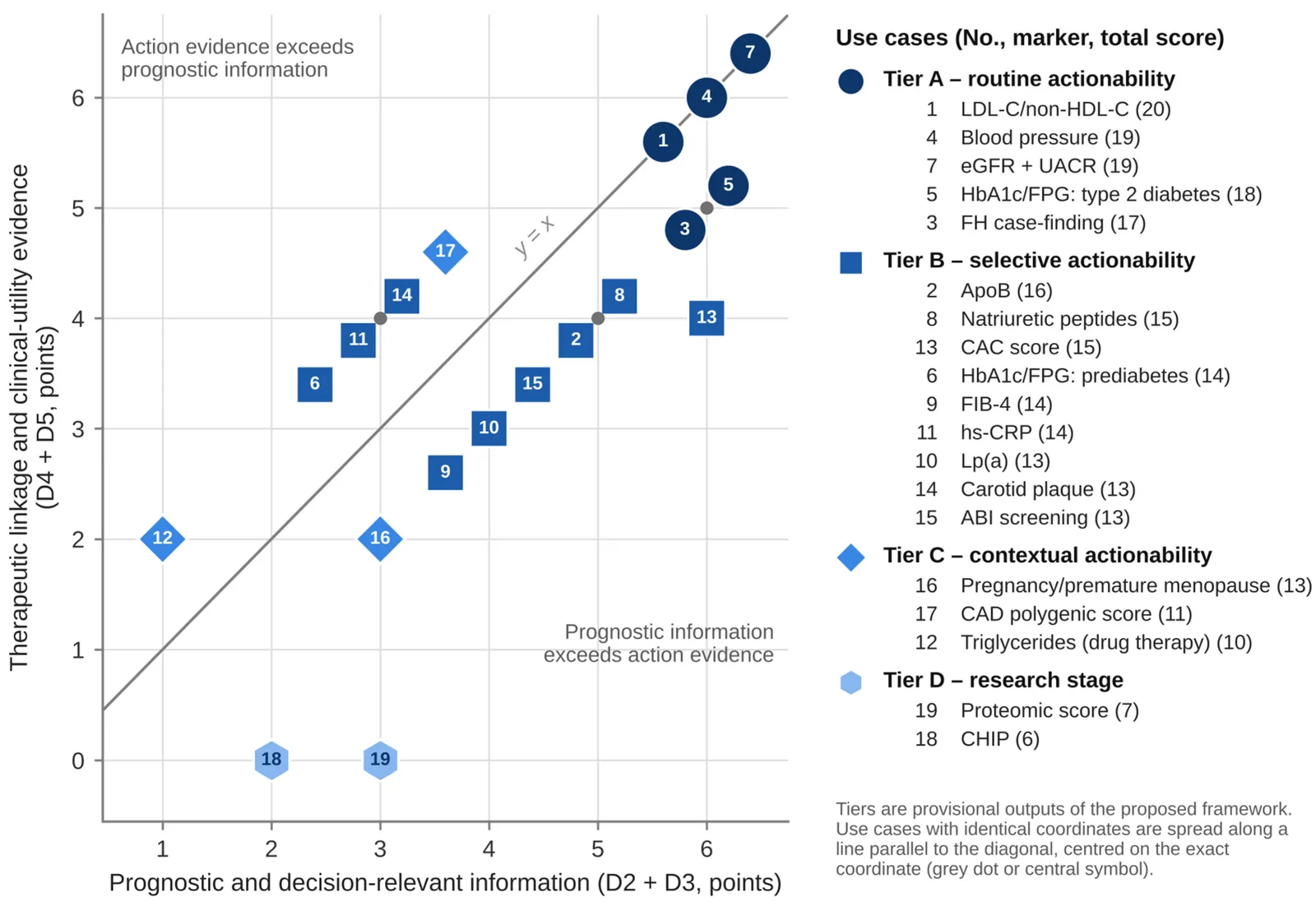
Actionability landscape of the 19 marker–context–action use cases. The horizontal axis shows the sum of the prognostic-validity and decision-relevant information scores (D2 + D3) and the vertical axis the sum of the therapeutic linkage and clinical-utility-evidence scores (D4 + D5); each sum ranges from 0 to 6. Symbol shape and shade denote the provisional tier, and numbers identify the use cases listed in Table 2 (total score in parentheses in the key). Where several use cases share the same coordinates, their symbols are spread for legibility along a line parallel to the diagonal and centred on the exact coordinate (marked by a grey dot or by the central symbol), so that each symbol keeps its true position relative to the diagonal; the displacement does not represent a difference in scores. Use cases below the diagonal have more prognostic information than evidence of benefit from action. Positions reflect ordinal author-assigned scores and describe the evaluated use cases, not the markers in general. Original visualisation generated from the domain scores reported in Table 2. ABI, ankle–brachial index; ApoB, apolipoprotein B; CAC, coronary artery calcium; CAD, coronary artery disease; CHIP, clonal haematopoiesis of indeterminate potential; eGFR, estimated glomerular filtration rate; FH, familial hypercholesterolaemia; FIB-4, Fibrosis-4 index; FPG, fasting plasma glucose; HbA1c, glycated haemoglobin; hs-CRP, high-sensitivity C-reactive protein; LDL-C, low-density lipoprotein cholesterol; Lp(a), lipoprotein(a); non-HDL-C, non-high-density lipoprotein cholesterol; UACR, urinary albumin-to-creatinine ratio.

Nineteen use cases spanning five marker classes were scored (Table 2). The rationale for each domain score, including the D3 route and the D7 subcomponent ratings, is given in Table S1 and the quantitative results of the studies that inform the scores in Table S2. In the reassessment performed during revision (Section 4.5), six scores were changed because the level assigned did not correspond to the cited evidence under the anchors and the construct definitions: decision-relevant information for the Lp(a) use case, from 2 to 1 (Section 5.3), and for the triglyceride and clonal haematopoiesis of indeterminate potential (CHIP) use cases, from 1 to 0, because no statistically significant increment had been shown (Section 5.3 and Section 5.5); transportability and equity for the CHIP and proteomic use cases, from 1 to 2, because limited validation corresponds to the level-2 rather than the level-1 anchor (Section 5.5); and prognostic validity for the ankle–brachial index (ABI) use case, from 2 to 3 (Section 5.4). Only the last changed a tier, moving the ABI use case from Tier C to Tier B. Within the proposed framework, five use cases were assigned to Tier A, nine to Tier B, three to Tier C and two to Tier D. Figure 2 plots the predictive and therapeutic components of the scores against each other.

### 5.1. Established Causal Risk Factors: The Actionability Benchmark

LDL-C is the reference against which other markers can be judged. Its causal role is established by genetic, epidemiological and randomised evidence [22], and statin therapy reduced major vascular events by 21% per 1 mmol/L LDL-C reduction (rate ratio 0.79; 95% CI 0.77–0.81), with rate ratios of 0.62 and 0.69 in the two lowest categories of baseline risk [23]. Measurement is inexpensive, standardised and repeatable, non-high-density lipoprotein (non-HDL) cholesterol can be derived from the same panel, and cholesterol fractions from that panel enter every major risk equation [10,13] (D3 = 3, route C). The use case scores the maximum on six domains. D7 scores 2: relative benefit is maintained across categories of baseline risk and largely irrespective of age and sex [23], but consistency across ancestry and socioeconomic groups has not been affirmatively documented, and access to and uptake of lipid-lowering therapy are uneven; in 41 low- and middle-income countries, 8.0% of eligible people used statins for primary prevention, and use was lower where health expenditure was lower [43].

Apolipoprotein B (apoB) shows how strong biology can coexist with limited evidence of clinical utility. When triglyceride-lowering variants in the lipoprotein lipase gene and LDL-C-lowering variants in the LDL receptor gene were compared per 10 mg/dL lower apoB, their associations with coronary heart disease were almost identical (odds ratios 0.771 and 0.773), suggesting that the number of apoB-containing particles, and not their cholesterol or triglyceride content, drives risk [44]. ApoB can therefore capture risk that LDL-C underestimates when the two are discordant, and the 2026 United States guideline considers apoB testing useful to assess residual risk once LDL-C and non-HDL cholesterol goals are met, particularly with elevated triglycerides, diabetes or low achieved LDL-C [9]. The decision-relevant information is specific to these contexts. In 26 prospective cohorts (139,581 participants), adding apoB and apolipoprotein A-I to conventional risk factors barely improved discrimination (C-index change 0.0006) [45], whereas among 13,015 statin-treated individuals followed for a median of 8 years, discordantly high apoB with LDL-C below the median was associated with a higher risk of myocardial infarction (HR 1.49; 95% CI 1.15–1.92) [46], indicating that LDL-C underestimates risk in this subgroup. Because this is a discordance analysis rather than a formal reclassification analysis, the score of 2 for decision-relevant information (route I) in the discordance contexts scored is a judgement; a score of 1 would leave the use case in Tier B (total 15). Statin trials lowered apoB, but apoB was not used to select participants or guide treatment, and no randomised trial has compared apoB-guided with LDL-C-guided treatment, so evidence for the decision this use case informs is genetic and observational (D5 = 1). D7 scores 2 because consistent performance across groups and equitable access have not been affirmatively shown. With a total of 16, the use case is assigned to Tier B. Within the framework, only a trial showing benefit from apoB-guided treatment would move it to Tier A; a one-point increase in any other domain would leave it in Tier B because of the second gating rule (Table S4A).

In familial hypercholesterolaemia (FH), the marker defines a high-risk state on its own [29] (D3 = 3, route S). In child–parent screening in primary care, one in 273 children carried an FH-causing mutation, and for every 1000 children screened, eight people (four children and four parents) were identified as having a positive screening result [47]. In a placebo-controlled trial in 214 children with FH, pravastatin lowered LDL-C by 24% and reduced the progression of carotid intima–media thickness over 2 years [48], and in the 20-year follow-up of this cohort, the cumulative incidence of cardiovascular events by the age of 39 years was 1%, compared with 26% among their affected parents [49]. Randomised evidence in FH therefore extends to validated intermediate outcomes but not to hard outcomes, which limits D5 to 2, and the implications of a genetic diagnosis for the person tested lower D6, but the use case is assigned to Tier A with a total of 17, on the boundary with Tier B; it is also the only assignment that depends on crediting route S with the maximum D3 score (Section 5.6).

Blood pressure has a similar evidence profile. In an individual-participant meta-analysis of 48 randomised trials including 344,716 participants, each 5 mmHg reduction in systolic BP lowered the risk of major cardiovascular events (HR 0.91; 95% CI 0.89–0.94 in people without prior cardiovascular disease), with no reliable evidence of heterogeneity across baseline BP categories [50]. Intensive treatment reduced major cardiovascular events further in high-risk adults without diabetes (HR 0.75; 95% CI 0.64–0.89), although serious adverse events of hypotension, syncope, electrolyte abnormalities and acute kidney injury were more frequent [51]; as for every use case, such treatment effects are reflected in the outcome evidence and not in D6. Systolic BP is a core constituent of the major risk equations [10,13] (D3 = 3, route C). As noted in Section 3.4, ambulatory measurement carries most of the prognostic information [34], and both the European and the United States hypertension guidelines recommend out-of-office measurement to confirm the diagnosis [52,53], so confirmation outside the clinic is part of the use case. D6 is reduced to 2 by overdiagnosis from unconfirmed office readings and by the burden of confirmation. D7 scores 2 because treatment and control remain unequal: in 2019, 47% of women and 38% of men with hypertension worldwide were treated, and 23% and 18%, respectively, were controlled, with wide variation between countries [54].

Glycaemic testing with HbA1c or fasting glucose in adults with overweight, obesity or other risk factors serves two decisions with different actionability, which are therefore scored as separate use cases. Detecting T2D identifies a condition whose cardiovascular risk European prevention guidance classifies as moderate to very high according to diabetes duration and target-organ damage [29]; diabetes also enters risk equations such as PREVENT [10] (D3 = 3, routes S and C) and confers about twice the risk of coronary heart disease and ischaemic stroke (HRs 2.00 and 2.27 in a meta-analysis of 102 prospective studies) [55], and it triggers glucose-lowering, lipid-lowering and organ-protective therapy. In newly diagnosed T2D, intensive glucose control was followed during 10 years of post-trial monitoring by fewer myocardial infarctions and deaths from any cause [56], and in T2D with CKD, sodium–glucose cotransporter 2 (SGLT2) inhibition and finerenone reduce kidney and cardiovascular events [35,36]. Screening itself, however, has not been shown to improve hard outcomes: in ADDITION-Cambridge, a cluster-randomised trial including 20,184 people at high risk of undiagnosed diabetes in 33 general practices, all-cause mortality over a median of 9.6 years did not differ between screening and unscreened practices (HR 1.06; 95% CI 0.90–1.25) [57]. Under the D5 reading rules, this null trial of the testing strategy limits clinical-utility evidence to 2 despite effective treatment of the condition detected. With a total of 18, the T2D-detection use case is assigned to Tier A.

Identifying prediabetes has a weaker profile. Prediabetes is only modestly associated with cardiovascular disease, with relative risks of 1.13–1.30 depending on its definition [58], and in people without diabetes, fasting glucose or impaired fasting glucose did not significantly improve the prediction of vascular disease [55], whereas adding HbA1c to conventional risk factors increased the C-index by 0.0018 (95% CI 0.0003–0.0033) without a significant improvement in reclassification [59], a statistically significant but marginal increment (D3 = 1, route I); route S is not credited because prediabetes is a risk factor rather than a disease [58] and European prevention guidance does not treat it as a high-risk category [29]. The action it triggers is lifestyle intervention. In the Diabetes Prevention Program, lifestyle intervention reduced the incidence of diabetes by 58% and metformin by 31% over a mean of 2.8 years [60], and in the Da Qing study, lifestyle intervention was followed after 30 years by fewer cardiovascular events (HR 0.74; 95% CI 0.59–0.92) and deaths from any cause (HR 0.74; 95% CI 0.61–0.89) [61]. The long-term follow-up of the Diabetes Prevention Program, however, found no reduction in major cardiovascular events with either metformin or lifestyle intervention [62]. Because the linked intervention has established efficacy on an intermediate outcome, incident diabetes, but inconsistent effects on hard outcomes, therapeutic linkage and clinical-utility evidence each score 2; because these trials enrolled people with impaired glucose tolerance, their application to prediabetes defined by HbA1c or fasting glucose is an extrapolation, and intervention data for these definitions are few [58]. Among 3412 older adults (mean age 75.6 years), regression to normoglycaemia or death was more frequent than progression to diabetes: of those with HbA1c-defined prediabetes, 9% progressed, 13% regressed and 19% died over a median of 5 years [63]. With a total of 14, the prediabetes use case is assigned to Tier B; the risk of labelling without benefit appears greatest in older adults.

### 5.2. Markers of Cardiometabolic Organ Involvement: Kidney, Heart and Liver

eGFR combined with UACR in people with diabetes or hypertension forms the highest-scoring use case outside the benchmark factors. In an individual-participant meta-analysis, adding UACR to conventional risk factors improved the C-statistic for cardiovascular mortality by 0.0139 and for HF by 0.0196, gains larger than those achieved by eGFR [64]. eGFR is a core predictor in the PREVENT equations, and UACR is an optional predictor in their extended form [10]; moderate or severe CKD defined by these markers is also classified as a high-risk or very-high-risk state in European prevention guidance [29] (D3 = 3, routes I, C and S). Therapeutic linkage is direct. In DAPA-CKD, which enrolled 4304 participants with an eGFR of 25–75 mL/min/1.73 m^2^ and a UACR of 200–5000 mg/g, dapagliflozin reduced the primary kidney and cardiovascular composite outcome (9.2% versus 14.5%; HR 0.61; 95% CI 0.51–0.72) irrespective of diabetes status [35], and in a pooled analysis of two outcome trials, finerenone reduced both cardiovascular and kidney outcomes in people with T2D and CKD [36]. Race-free creatinine–cystatin C equations have improved the accuracy and equity of eGFR estimation [65], and current kidney and CKM guidance recommends using both eGFR and UACR to characterise CKD [2,32]. Yet albuminuria testing remains uncommon in people with diabetes and even less common in those with hypertension [12]. The combination of near-maximal scores and incomplete uptake is a central finding of this synthesis.

Natriuretic peptides are among the strongest prognostic markers in apparently healthy people. In an individual-participant meta-analysis of 95,617 participants from 40 prospective studies, people in the top third of N-terminal pro-B-type natriuretic peptide (NT-proBNP) had twice the risk of coronary heart disease, stroke or HF of those in the bottom third (risk ratio 2.00; 95% CI 1.77–2.26), and adding NT-proBNP to conventional risk factors improved the C-index by 0.019 [66]. In people with diabetes, an American Diabetes Association consensus report supports screening with natriuretic peptides or high-sensitivity cardiac troponin to detect asymptomatic stages of HF [67]. Randomised evidence of utility exists but is limited, and it spans both peptides. In PONTIAC, which randomised 300 patients with T2D, NT-proBNP above 125 pg/mL and no cardiac disease, intensified renin–angiotensin system blockade and beta-blockade reduced hospitalisations or deaths from cardiac causes over 2 years (HR 0.351; 95% CI 0.127–0.975) [37]. In STOP-HF, which enrolled 1374 people with cardiovascular risk factors from 39 primary care practices, B-type natriuretic peptide (BNP) screening, with echocardiography and collaborative care for those with values of 50 pg/mL or higher, reduced the prevalence of asymptomatic left ventricular dysfunction with or without newly diagnosed HF (odds ratio 0.55; 95% CI 0.37–0.82) [38]. Because the consensus recommendation refers to measurement of a natriuretic peptide without specifying BNP or NT-proBNP [67], the use case is defined for natriuretic peptides with analyte-specific thresholds; numerical results are not interchangeable between the two assays, which limits measurement readiness (D1 = 2). Both trials were small, and neither has been replicated at scale; the use case is assigned to Tier B (total 15). The triad matters here: in unselected adults without diabetes or other cardiovascular risk factors, the same measurement has no trial evidence of utility, and when scored for that context, its therapeutic linkage and clinical-utility scores would each fall by one point, moving it to Tier C.

MASLD links the liver to the cardiometabolic continuum. In a meta-analysis of 36 longitudinal studies including more than 5.8 million individuals, MASLD (then termed non-alcoholic fatty liver disease) was associated with a higher risk of fatal or non-fatal cardiovascular events (HR 1.45; 95% CI 1.31–1.61), and the risk rose with the stage of fibrosis (HR 2.50; 95% CI 1.68–3.72) [4]. The Fibrosis-4 index (FIB-4) itself carries prognostic information for liver outcomes: in a Swedish population cohort in which it could be calculated for 126,941 individuals followed for up to 27 years, non-invasive fibrosis scores including FIB-4 identified people at higher risk of incident severe liver disease, with modest accuracy in the general population and better performance in people with risk factors for fatty liver disease [68]. Because severe liver disease is one of the hard outcomes of the framework, this evidence supports D2 = 2 for FIB-4, whereas advanced fibrosis, the state it screens for, is treated as an intermediate outcome. The joint European guideline recommends a stepwise approach using FIB-4 followed by imaging such as transient elastography to rule advanced fibrosis in or out in people with cardiometabolic risk factors, and it supports resmetirom for non-cirrhotic steatohepatitis with significant fibrosis where approved [3], a therapy whose benefit has so far been shown on histological endpoints (D4 = 2). A two-tier primary care pathway detected five times more advanced fibrosis and cirrhosis than standard care (odds ratio 5.18; 95% CI 2.97–9.04) [69]. Because no hard-outcome evidence for FIB-4-guided pathways is yet available, D5 scores 1, and the use case is assigned to Tier B (total 14).

### 5.3. Circulating Risk Enhancers: Lipoprotein(a), Inflammation and Triglycerides

Lp(a) shows most clearly how causal evidence and actionability can diverge. Genetic evidence supports causality [25]. In a large national biobank followed for a median of 11.2 years, the association with incident ASCVD appeared linear across the distribution, with an HR of 1.11 (95% CI 1.10–1.12) per 50 nmol/L [70], and in the Women’s Health Study a single baseline measurement predicted cardiovascular events over 30 years [71]. Guideline and consensus documents support measuring Lp(a) at least once in adulthood [8,9,33]. The European update treats values above 50 mg/dL or above 105 nmol/L as a risk modifier [8], and the United States guideline treats values of 125 nmol/L or more (50 mg/dL or more) as risk-enhancing [9]. That the two guidelines pair 50 mg/dL with different molar values (105 and 125 nmol/L) reflects the lack of a standard conversion factor [33]. Across the population, however, the incremental information is marginal: adding Lp(a) to conventional risk factors increased the C-index by 0.0016 in 24 prospective cohorts (133,502 participants), net reclassification improved by less than 1%, and an estimated 4.1% of people at intermediate risk would be reclassified to high risk [45]. Recognition as a risk modifier or risk-enhancing factor is not itself evidence of decision-relevant information, and under the refined anchor the D3 score was changed during revision from 2 to 1 (route I). The decisive gap concerns therapeutic linkage. Mendelian randomisation suggests that an absolute Lp(a) reduction of 101.5 mg/dL (95% CI 71.0–137.0) would be needed to match the effect on coronary risk of a 1 mmol/L (38.67 mg/dL) reduction in LDL-C [72]. No peer-reviewed outcome trial has yet shown that lowering Lp(a) reduces cardiovascular events. According to topline results announced by its sponsor on 4 September 2026, the phase 3 Lp(a)HORIZON trial (NCT04023552), which compared pelacarsen with placebo in 8323 patients with established cardiovascular disease and elevated Lp(a), did not meet its primary endpoint; peer-reviewed results were not available at the time of revision. In accordance with Section 2, these results were not used to score any domain; the D4 and D5 scores already reflect the absence of an Lp(a)-lowering therapy shown to reduce cardiovascular events. Lp(a) therefore has three distinct roles: it is a causal risk factor; it is currently actionable only along the triage route, by prompting earlier and more intensive LDL-C lowering and stricter control of other risk factors; and it would become a therapeutic target only if outcome trials demonstrate benefit from lowering it. Because its concentration is predominantly genetically determined [33], an elevated value also has implications for first-degree relatives, which is the rationale for cascade testing. Assay heterogeneity, isoform-dependent measurement and the coexistence of mass and molar units limit measurement readiness [33], and a single result without a specific therapy carries some risk of labelling. With a total of 13, the evaluated Lp(a) use case remains in Tier B within the proposed framework, on the boundary with Tier C (Section 5.6).

hs-CRP shows that a marker can inform treatment decisions without being causal. In an individual-participant analysis of 38 prospective cohorts with CRP measurements (166,596 participants), adding CRP to conventional risk factors increased the C-index by only 0.0039, and targeted assessment of CRP or fibrinogen in people at intermediate risk was estimated to prevent one additional event over 10 years for every 400–500 people screened [73]. Mendelian randomisation did not support a causal effect of CRP [24]. Nevertheless, in JUPITER, among 17,802 people with LDL-C below 130 mg/dL (3.4 mmol/L) and hs-CRP of at least 2 mg/L, rosuvastatin reduced first major cardiovascular events (HR 0.56; 95% CI 0.46–0.69) [18]. The European update considers only persistently elevated values, above 2 mg/L [8]. No anti-inflammatory therapy is established for primary prevention, and the use case of hs-CRP as a risk enhancer informing statin initiation is assigned to Tier B (total 14).

Triglycerides show why the use case must specify the action. The scored triad is not triglyceride concentration as a causal exposure but the use of a moderately elevated triglyceride level to select adults for triglyceride-directed drug therapy. As a prognostic marker, triglyceride concentration was not associated with coronary heart disease after adjustment for high-density lipoprotein (HDL) and non-HDL cholesterol (HR 0.99; 95% CI 0.94–1.05) in an individual-participant analysis of 302,430 people [74], and genetic evidence indicates that the risk carried by triglyceride-rich lipoproteins tracks the number of apoB-containing particles [44] (D2 = 1). Because no statistically significant increment in prediction has been shown (adding triglycerides to conventional risk factors that included total and HDL cholesterol changed the C-index by 0.0000 in 24 cohorts [45]), decision-relevant information scores 0 (route I); this score was changed during revision from 1 to 0. The outcome trials addressed related but different therapeutic questions. Icosapent ethyl, a purified eicosapentaenoic acid ester, reduced ischaemic events in REDUCE-IT (HR 0.75; 95% CI 0.68–0.83), with more frequent hospitalisation for atrial fibrillation or flutter (3.1% versus 2.1%), but not significantly in its primary-prevention cohort (HR 0.88; 95% CI 0.70–1.10) [75]. An eicosapentaenoic and docosahexaenoic acid formulation compared with corn oil in STRENGTH (HR 0.99; 95% CI 0.90–1.09) [76] and the fibrate pemafibrate in PROMINENT (HR 1.03; 95% CI 0.91–1.15), which lowered triglycerides by 26% but raised apoB by 5% [77], did not reduce events. Differences in agents, comparators and effects on apoB offer plausible mechanistic explanations for these discordant results, consistent with the genetic evidence [44], but none has been established directly. Because the available outcome trials have yielded heterogeneous results and do not establish the clinical benefit of apoB-guided triglyceride-lowering therapy, particularly in primary prevention, therapeutic linkage and clinical-utility evidence each receive a score of 1, assigning this use case to Tier C (total score 10). In primary care, an elevated triglyceride level is therefore better used as a prompt to assess non-HDL cholesterol or apoB and to address secondary causes than as a general indication for triglyceride-directed drug therapy, whose benefit has been shown only for icosapent ethyl in statin-treated people with established cardiovascular disease or diabetes and other risk factors [75].

### 5.4. Subclinical Atherosclerosis Imaging

CAC scoring provides substantial decision-relevant information in adults at intermediate risk. In MESA, among 6722 participants from four ethnic groups, CAC scores above 300 were associated with a 9.7-fold higher adjusted risk of any coronary event than a score of zero, and each doubling of the score increased the risk of a major coronary event by 15–35% [26]. Among intermediate-risk participants, CAC improved discrimination and reclassification more than the ABI, hs-CRP, family history, carotid intima–media thickness or brachial flow-mediated dilation [39]. Of statin-eligible participants, 44% had a CAC score of zero, with an observed ASCVD event rate of 4.2 per 1000 person-years [40], which gives CAC a de-risking role in addition to upward reclassification (D3 = 3, route I). Both current dyslipidaemia guidelines support selective CAC scoring. The European update treats subclinical coronary atherosclerosis on imaging or an increased CAC score as a risk modifier [8], and the United States guideline links any detectable CAC to an LDL-C goal below 100 mg/dL (2.6 mmol/L), with lower targets at higher scores [9]. The limitations lie in utility and harms. In EISNER, randomisation to CAC scanning among 2137 volunteers led to favourable changes in systolic BP and LDL-C, and in waist circumference among participants with increased abdominal girth, without increasing downstream testing or costs [41]. In DANCAVAS, however, which randomised 46,611 men aged 65–74 years to an invitation to screening that included CAC scoring or to usual care, screening did not significantly reduce all-cause mortality over 5.6 years (HR 0.95; 95% CI 0.90–1.00), although stroke was reduced (HR 0.93; 95% CI 0.86–0.99) [42]. The United States Preventive Services Task Force judged the evidence insufficient to assess the balance of benefits and harms of adding CAC, the ABI or hs-CRP to traditional risk assessment [78]. A CAC scan delivers an effective radiation dose of about 1 mSv with contemporary scanners [79], and incidental findings can initiate cascades of care, which most physicians in a national survey reported had caused psychological or financial harm to their patients [80]; together, these lower D6 to 1. The use case is assigned to Tier B (total 15).

Carotid ultrasound behaves differently depending on what is measured. Intima–media thickness improves classification only marginally [39], whereas plaque is a more specific marker of disease: in a meta-analysis of 11 population-based studies including 54,336 participants, plaque predicted myocardial infarction more accurately than intima–media thickness, although discrimination remained modest (area under the curve 0.64 versus 0.61) [81]. Evidence on its incremental value is heterogeneous. In MESA, adding plaque presence to traditional risk factors increased the C-statistic for cardiovascular disease from 0.756 to 0.760, less than the addition of CAC presence did (0.776) [82], whereas larger improvements were reported for plaque combined with intima–media thickness in the Atherosclerosis Risk in Communities (ARIC) study (net reclassification improvement 9.9%) [83] and for three-dimensional plaque burden in older adults followed for a median of 2.7 years (net reclassification improvement 23%) [84]. Because the use case scored is the detection of plaque on standard ultrasound, the most direct evidence was credited, which indicates a marginal increment (D3 = 1, route I). In VIPVIZA, pictorial information on carotid ultrasound findings, showing plaque and intima–media thickness expressed as vascular age, was given to 1749 participants and their physicians and reinforced by a nurse telephone call; at 1 year, the Framingham risk score, a primary outcome, was lower than in the control group (1783 randomised), in an analysis of the 3175 participants who completed follow-up (difference 1.07; 95% CI 0.11–2.03), with a parallel difference in the Systematic Coronary Risk Evaluation (SCORE) [85]. Calculated risk scores are predicted-risk constructs, not measured intermediate outcomes, and are not used for scoring. The clinical-utility score rests instead on LDL-C, a validated intermediate outcome, which was lower in the intervention group at 1 year (difference 0.17 mmol/L; 95% CI 0.11–0.24) and at 3 years (0.23 mmol/L; 95% CI 0.16–0.30) [86], the same type of evidence that supports the score for CAC [41]. The effect is modest, and no hard-outcome data exist (D5 = 2). The use case is assigned to Tier B with a total of 13, on the boundary with Tier C.

Studies of the ABI disagree about its incremental value but not about its prognostic strength. In the ABI Collaboration, an individual-participant meta-analysis of 16 population cohorts, an ABI of 0.90 or less, compared with a normal ABI of 1.11–1.40, was associated with a 4.2-fold higher 10-year cardiovascular mortality in men (95% CI 3.3–5.4) and a 3.5-fold higher cardiovascular mortality in women (95% CI 2.4–5.1), the association persisted after adjustment for the Framingham risk score, and adding the ABI to the Framingham score would have changed the risk category of 19% of men and 36% of women; the cohorts were based in Australia, Europe and the United States and were predominantly white, although some included Japanese American, American Indian, Black or Hispanic participants [87]. Evidence from other populations is sparser. In a small single-centre cross-sectional series from Iran, significant coronary artery disease was found on angiography in 41 of 48 patients (85%) with peripheral artery disease (PAD) scheduled for vascular surgery, more often in those with hypertension [88]; such a selected surgical series illustrates the clustering of coronary disease and risk factors once PAD is established, but it does not inform the yield or utility of ABI screening in asymptomatic adults and was not used for scoring. In intermediate-risk MESA participants, the increment in the ABI was small [39]. Differences in the populations studied (general versus intermediate risk) and in the reclassification metrics used may largely explain the discrepancy. Although an ABI of 0.90 or less is the diagnostic threshold for PAD [89], route S was not credited because recent prevention guidance describes the ABI as a measure of atherosclerosis with utility in risk assessment and limited evidence of utility for guiding clinical care [2]; crediting it would raise the total to 15 without changing the tier (Table S1). The index is also unreliable when arterial stiffness or medial calcification makes the ankle arteries poorly compressible, which produces falsely high values above 1.40 [89]; such calcification is common in diabetes and advanced CKD, and a toe–brachial index can then be used [89]. Because this limitation affects measurement in particular groups, it is scored under transportability (D7) and not under prognostic validity; given the strength, graded (reverse J-shaped) form and independence of the association, and its consistency across these cohorts, especially in men (results in women were more heterogeneous) [87], D2 was changed during revision from 2 to 3. The randomised evidence on screening is conflicting. In the Aspirin for Asymptomatic Atherosclerosis (AAA) trial, 3350 people with an ABI of 0.95 or less, identified by screening 28,980 adults, were randomised to aspirin or placebo; vascular events did not differ after a mean of 8.2 years (HR 1.03; 95% CI 0.84–1.27), and major haemorrhage requiring admission was numerically more frequent with aspirin [90]. In VIVA, which randomised 50,156 men aged 65–74 years to an invitation to screening for abdominal aortic aneurysm, PAD and hypertension, with aspirin and statin therapy offered to those with vascular disease, or to no screening, all-cause mortality was reduced over a median of 4.4 years (HR 0.93; 95% CI 0.88–0.98) [91], whereas DANCAVAS, which combined the ABI with CAC scoring and other tests, did not significantly reduce it [42]. Neither combined programme isolates the contribution of the ABI. An abnormal screening result therefore warrants clinical assessment rather than automatic antiplatelet therapy: in asymptomatic PAD, the 2024 United States guideline on lower-extremity PAD considers single antiplatelet therapy reasonable on the basis of expert opinion because trials such as AAA, which enrolled people with an ABI of 0.95 or less, did not provide adequate evidence specific to this group [92]; the decision also takes the individual’s bleeding risk into account. The action scored is statin therapy with risk-factor intensification, for which the ABI identifies people with higher expected benefit (D4 = 2). With conflicting randomised evidence (D5 = 1), the use case totals 13 and is assigned to Tier B, on the boundary with Tier C, and it is among the least stable assignments in the sensitivity analyses (Section 5.6).

### 5.5. Genomic, Multi-Omic and Life-Course Markers

Polygenic risk scores (PRS) for coronary artery disease identify 8% of the population with more than a 3-fold increase in risk, a proportion 20 times larger than that of carriers of monogenic mutations conferring comparable risk [93]. Their increment in 10-year prediction beyond conventional risk factors is small. In one large population cohort, the C-statistic was 0.76 for the pooled cohort equations and 0.78 when a PRS was added, with a net reclassification improvement of 4.0% [94], and among middle-aged white participants of two United States cohorts, adding a PRS did not significantly improve discrimination or risk reclassification [95]. In analyses of statin trials, participants with the highest genetic risk derived the largest relative and absolute benefit from therapy [96]. Disclosure of a genetic risk score in the MI-GENES trial of 203 participants lowered LDL-C at 6 months (96.5 versus 105.9 mg/dL), mainly through more frequent statin initiation (39% versus 22%) [97], and post hoc follow-up over a median of 9.5 years suggested fewer major adverse cardiovascular events after disclosure, an observation limited by very small numbers of events [98]. Communicating DNA-based risk estimates did not significantly change smoking, diet or physical activity in a meta-analysis of randomised and quasi-randomised trials [99]. PRS are several times more accurate in people of European ancestry than in other ancestries [100], and a 2025 clinical consensus statement of the European Society of Cardiology noted that its guidelines do not currently advocate routine clinical use of PRS and that such information is most relevant when 10-year risk is close to a treatment threshold, as in people at intermediate risk considering statin therapy [101]. The use case is assigned to Tier C (total 11).

CHIP was associated with a 1.9-fold risk of coronary heart disease in two prospective cohorts and a 4.0-fold risk of early-onset myocardial infarction in two retrospective case–control studies [102], but the evidence cited does not show that CHIP improves risk prediction beyond conventional risk factors; decision-relevant information therefore scores 0 (route I), changed during revision from 1. In an Icelandic cohort, adding a risk score derived from large-scale plasma proteomics to clinical risk factors that did not include cholesterol or blood pressure values increased the C-index by 0.014 (95% CI 0.002–0.028), a statistically significant but modest increment [103] (D3 = 1). For both use cases, D7 was changed during revision from 1 to 2 because consistent performance across populations has not been shown, which corresponds to the level-2 anchor, rather than shown to be reduced (Table S1). Neither is linked to a proven intervention or available as a standardised clinical test, and both use cases are assigned to Tier D.

Life-course and sex-specific history is the least costly marker class. Pre-eclampsia was associated with a 2.16-fold risk of later ischaemic heart disease and a 3.70-fold risk of hypertension [104], and among 144,260 postmenopausal women in the UK Biobank, natural and surgical menopause before the age of 40 years were associated with a higher incidence of a composite of cardiovascular diseases after adjustment for conventional risk factors (HRs 1.36 and 1.87) [105]. The two exposures are scored together because they are female-specific history items that are listed among the European risk modifiers [8] and prompt the same action. In the Norwegian HUNT study, however, adding pregnancy complications to established risk factors improved prediction only slightly (C-index difference 0.004; 95% CI 0.002–0.006) [106]. The action that this history supports is earlier and more regular measurement of BP, glucose and lipids, not a specific therapy, and no trial has shown that acting on it improves outcomes. Surveillance of this kind is generic advice under the D4 anchor, so therapeutic linkage scores 1, and the first gating rule places the use case in Tier C although its total of 13 would otherwise fall in Tier B. Obtaining the history costs little and carries no measurement harm (D6 = 3), so it can be collected broadly (Section 6.3); the result then informs individual decisions, for example, as a risk enhancer when a treatment decision is otherwise uncertain.

Social and contextual markers were not scored as separate use cases because the action they prompt is rarely a specific clinical therapy. They nevertheless enter risk estimation, with a social deprivation index offered as an optional predictor in PREVENT [10] and social deprivation listed among the European risk modifiers [8]. Their main role in the framework is to inform the equity subcomponent of D7.

### 5.6. Sensitivity of Tier Assignments to Scores, Thresholds, Weights and Rules

Because the scores are judgements and the thresholds heuristic, the dependence of the tier assignments on these choices was examined in deterministic sensitivity analyses (Table S4A,B). The single-domain analysis, the one-point uniform threshold shifts and the removal of the gating rules, which were reported in the original version, were repeated with the revised scores; the two-domain, two-point and single cut-point threshold, combined, weighting, leave-one-domain-out and D3-capping analyses were added during revision of the manuscript. First, each domain score of each use case was changed by one point in either direction, within the 0–3 range, and the tier recomputed. Of 222 such perturbations, 40 (18.0%) changed the tier (Table S4A). The changes were concentrated in four use cases that lie on tier boundaries and change tier with a one-point reduction in any domain: FH case-finding (total 17; Tier A to B) and the Lp(a), carotid plaque and ABI use cases (total 13; Tier B to C), which together accounted for 28 of the 40 changes. T2D detection changed tier with a one-point reduction in therapeutic linkage or clinical-utility evidence, ten other use cases each changed with a single perturbation, always of one of these two domains, and no single-domain perturbation changed the tiers of triglyceride-directed therapy, PRS, CHIP and proteomic scores. Therapeutic linkage was the most influential domain (14 of 40 changes), which reflects its role in the gating rules. The analysis also identifies the evidence that matters most: a single additional point for clinical utility would move apoB into Tier A, and a single additional point for therapeutic linkage would move the pregnancy-complication and premature-menopause use case into Tier B. Second, changing two domains simultaneously by one point each altered the tier in 334 of 1124 combinations (29.7%; Table S4B), and at least one combination changed the tier of 18 of the 19 use cases. Third, lowering all thresholds by one point changed no assignment, whereas raising them by one point moved the four boundary use cases; shifts of two points also moved T2D detection to Tier B, the prediabetes, FIB-4 and hs-CRP use cases to Tier C and the triglyceride use case to Tier D when thresholds were raised, and the PRS use case to Tier B and the proteomic score to Tier C when they were lowered. Fourth, when the single-domain perturbations were repeated with thresholds shifted by −1, 0 or +1 point, 141 of 666 scenarios (21.2%) differed from the published tier. Fifth, two interpretable alternatives to equal weighting, defined by the suspected overlaps between domains and not adjusted to reproduce the existing assignments, were applied with the cut-points rescaled in proportion to the maximum attainable total (Table S4B): counting D4 and D5 once (each weighted 0.5) moved FH case-finding to Tier B and the carotid plaque and proteomic-score use cases to Tier C, and counting D1, D6 and D7 once (each weighted one third) moved the Lp(a), carotid plaque and ABI use cases to Tier C and the triglyceride use case to Tier D. Several weighted totals lie within 0.1 point of a rescaled cut-point, so these results depend on the rescaling convention. Removing each domain from the total in turn changed between one and seven assignments, mostly among the boundary use cases. Finally, capping routes C and S of D3 at 2 changed only FH case-finding (to Tier B), and removing the gating rules changed only the pregnancy-complication and premature-menopause use case (to Tier B); the second gating rule did not determine any current assignment. Table S4B summarises, for each use case, the perturbations that change its tier, and three patterns emerge. Four assignments lie on a tier boundary and change with a one-point reduction in any domain and with a one-point rise in all thresholds (FH case-finding, Lp(a), carotid plaque and the ABI). Eleven further assignments change with a one-point change in therapeutic linkage or clinical-utility evidence but not with a one-point threshold shift (LDL-C, apoB, BP, T2D detection, prediabetes, eGFR with UACR, natriuretic peptides, FIB-4, hs-CRP, CAC, and pregnancy complications and premature menopause); four of them (T2D detection, prediabetes, FIB-4 and hs-CRP) also change with two-point threshold shifts. Thus 15 of the 19 assignments depend on a single-point judgement in D4 or D5. This is the intended effect of the gating rules, which tie the tier to therapeutic linkage and clinical-utility evidence, but it also means that the tiers are only as reliable as these two judgements. The remaining four use cases did not change with any single-domain perturbation: the triglyceride, PRS and proteomic-score use cases changed only with simultaneous changes of two points, in two domains or in all thresholds, or with alternative weightings, and CHIP with none of the perturbations examined. These summaries were compiled during revision and were not prespecified; they describe sensitivity to the perturbations examined, not validity, because all perturbations were applied to the same author-assigned scores and thresholds. All perturbations were counted as equally plausible, so the proportions reported are not estimates of the probability that a tier is misclassified. Because most assignments depend on single-point judgements, individual tier assignments should be regarded as provisional and should not be interpreted as definitive clinical priorities.

## 6. Integrative Synthesis: From Actionability Scores to Primary Care Pathways

### 6.1. Prediction Is Not Actionability

Viewed together (Figure 2), the scores suggest that prognostic strength and actionability are only loosely coupled. The five Tier A use cases share three features: standardised and largely inexpensive measurement; a marker that is either causal or a direct index of organ involvement or disease; and randomised evidence that treating people selected by the marker reduces hard outcomes. Two are partial exceptions: in FH, benefit is inferred from LDL-C lowering and long-term cohort data because placebo-controlled hard-outcome trials are lacking, and for T2D detection the screening strategy itself did not reduce mortality in the one trial that tested it. None of the newer markers combines all three features. CAC reaches the maximum score for decision-relevant information through substantial reclassification (route I), whereas the benchmark factors do so as constituents of risk equations or by defining high-risk states (routes C and S), but it falls short on clinical utility and harms. Proteomic scores and CHIP carry prognostic information without any therapeutic linkage. The hs-CRP and carotid plaque use cases, by contrast, are assigned to Tier B despite modest predictive increments because measurement is feasible and the results lead either to a therapy that has been effective in the population they select or to a measurable reduction in LDL-C; the Lp(a) and ABI use cases reach the lower boundary of the same tier mainly through strong prognostic validity and an indirect pathway to statin therapy. The PRS use case shares the profile of the hs-CRP and carotid plaque use cases but remains in Tier C because of limited measurement readiness and transportability.

Lp(a) occupies an unusual position. Its causal status is well supported, but its incremental information across the population is marginal and, in the absence of a proven Lp(a)-lowering therapy, its actionability is borrowed from LDL-C lowering and global risk management. The framework does not contradict guidance supporting one-time Lp(a) measurement in adults [8,9]: obtaining a single result carries little cost or harm, and the distinction between acquiring information and acting on it (Section 4.3) allows broad testing to coexist with selective action. What the framework adds is the recognition that the value of the result currently lies in triage and that this value would change substantially if outcome trials were positive. This reasoning is consistent with the convergence of the two major dyslipidaemia guidelines, despite different risk equations, on selective use of CAC and on one-time measurement of Lp(a) [8,9].

Actionability also depends on the health system. Where computed tomography, natriuretic peptide assays or apoB and Lp(a) measurements are expensive or unavailable, measurement readiness falls, and the corresponding use cases may drop a tier; where population registries and structured recall already exist, the burden of follow-up falls, and the totals of some Tier B use cases rise, although none could reach Tier A without stronger therapeutic linkage or clinical-utility evidence (second gating rule). The sensitivity analysis in Section 5.6 shows which use cases are most sensitive to such local rescoring. The framework is intended to be re-applied locally, not transferred unchanged; implications for resource-limited settings are considered in Section 6.6.

A second pattern concerns the direction of evidence gaps. For most Tier B and C use cases, the missing evidence is not more prognostic data but trials that randomise people to marker-guided versus usual care with clinically meaningful endpoints. Prognostic studies continue to accumulate because they are cheaper and faster, whereas trials of marker-based strategies such as ADDITION-Cambridge, DANCAVAS, EISNER, VIPVIZA, VIVA, PONTIAC and STOP-HF remain few and have produced mixed results [37,38,41,42,57,85,91].

### 6.2. The Underuse–Overuse Paradox

Several of the highest-tier use cases also have substantial implementation gaps. Albuminuria testing, whose use case scores 19 of 21 points in people with diabetes or hypertension, is performed in a minority of eligible patients [12], and preventive care for people at high cardiovascular risk in European primary care remains poorly developed [11]. At the same time, attention and resources increasingly flow to markers whose use cases fall in Tiers C and D. Trials of general health checks, which offer many tests to adults unselected for disease or risk factors, found little or no effect on total or cardiovascular mortality [17]. A practical implication of the framework is therefore a sequencing principle: in most primary care populations, closing gaps in Tier A testing and treatment should generally precede the introduction of markers from lower-tier use cases because the expected gain per person tested is likely to be greatest where therapeutic linkage and clinical-utility evidence are strongest.

### 6.3. A Proposed Conceptual Primary Care Pathway

**Figure 3:**
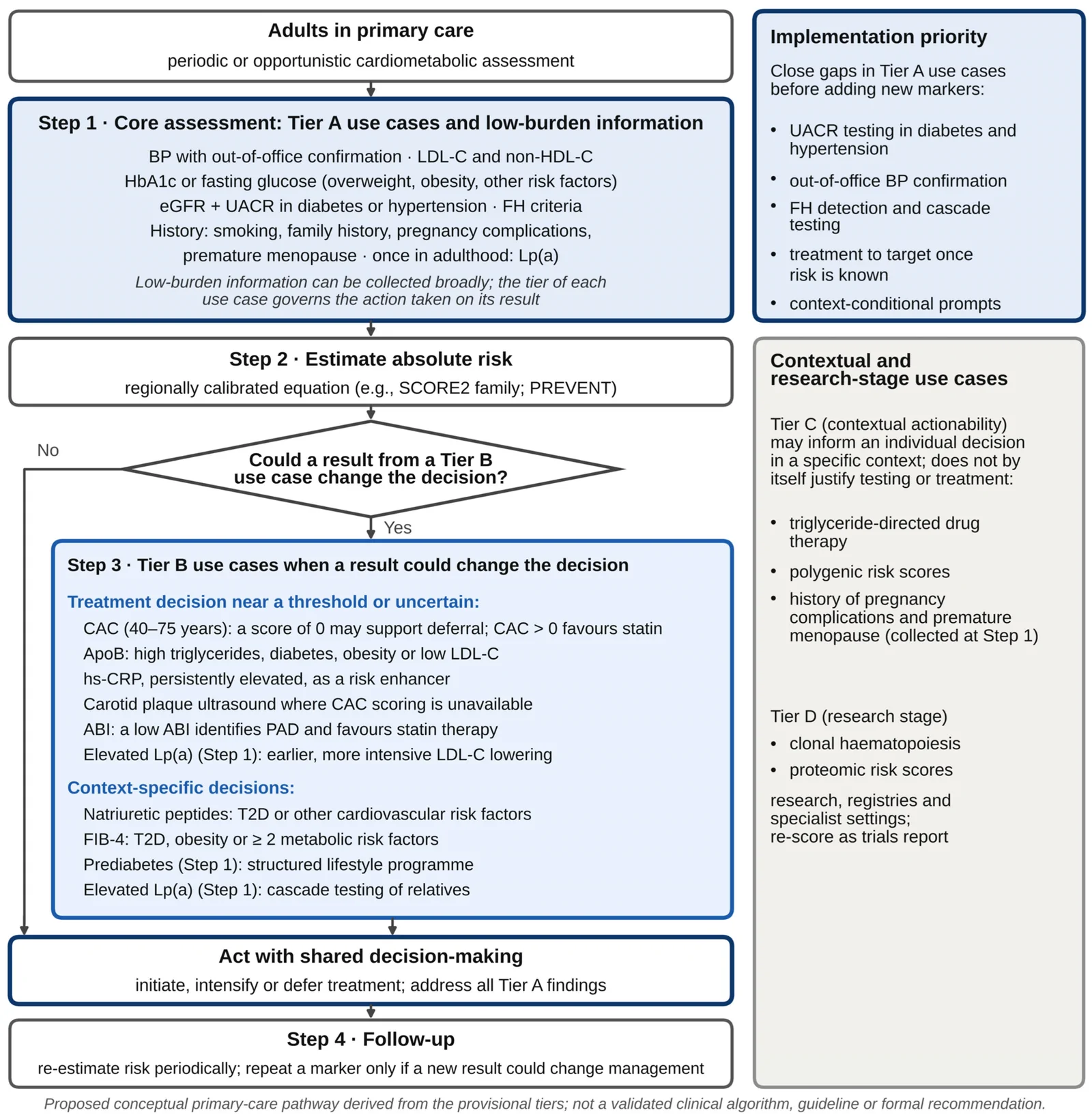
Proposed conceptual primary care pathway derived from the provisional tier assignments of the framework. Tier A use cases and low-burden information, including a history of pregnancy complications and premature menopause and a single Lp(a) measurement, are obtained at Step 1, before absolute risk is estimated with a regionally calibrated equation (Step 2). Acquiring information broadly does not imply routine action: each result is acted on according to the tier of its use case. A test from a Tier B use case is added, or its result acted on, only when the result could change a specific decision (Step 3): the treatment decision when risk lies near a threshold or the decision is otherwise uncertain or a context-specific decision such as further cardiac or liver evaluation, referral to a structured lifestyle programme or cascade testing. Markers are repeated only when a new result could change management (Step 4). Tier C information may inform an individual decision in a specific context without by itself justifying testing or treatment, and Tier D use cases are confined to research, registries and specialist settings. Original schematic developed by the authors; the pathway is not a validated clinical algorithm, guideline or formal recommendation. ABI, ankle–brachial index; ApoB, apolipoprotein B; BP, blood pressure; CAC, coronary artery calcium; eGFR, estimated glomerular filtration rate; FH, familial hypercholesterolaemia; FIB-4, Fibrosis-4 index; HbA1c, glycated haemoglobin; hs-CRP, high-sensitivity C-reactive protein; LDL-C, low-density lipoprotein cholesterol; Lp(a), lipoprotein(a); non-HDL-C, non-high-density lipoprotein cholesterol; PAD, peripheral artery disease; PREVENT, Predicting Risk of cardiovascular disease EVENTs; SCORE2, Systematic Coronary Risk Evaluation 2; T2D, type 2 diabetes; UACR, urinary albumin-to-creatinine ratio.

The framework can be translated into a proposed conceptual pathway with four steps (Figure 3). The pathway is derived from the provisional tier assignments and has not been evaluated as a clinical algorithm. Step 1 applies the Tier A use cases to eligible adults, together with information whose acquisition costs little: a history that includes pregnancy complications and premature menopause and a single Lp(a) measurement in adulthood. Collecting this information broadly does not make every result routinely actionable; the tier of each use case governs what is done with the result. Step 2 estimates absolute risk with a regionally calibrated equation, such as the SCORE2 family in Europe [13] or PREVENT in the United States [10]. Step 3 adds a test from a Tier B use case, or acts on its result, only when the result could change a specific decision; when no such decision is pending, clinicians act directly with shared decision-making. For the treatment decision, Step 3 applies when the estimate lies near a threshold or the decision is otherwise uncertain: CAC in adults aged 40–75 years at borderline or intermediate risk, apoB in the discordance contexts of high triglycerides, diabetes, obesity or low LDL-C, persistently elevated hs-CRP as a risk enhancer, carotid plaque ultrasound where CAC scoring is unavailable, the ABI, in which a value of 0.90 or less identifies PAD and favours statin therapy, and an elevated Lp(a) obtained at Step 1, which favours earlier and more intensive LDL-C lowering. Other Tier B use cases inform context-specific decisions that do not depend on the treatment threshold: natriuretic peptides in T2D or other cardiovascular risk factors, FIB-4 in T2D, obesity or multiple metabolic risk factors, referral to a structured lifestyle programme when prediabetes is detected at Step 1, and cascade testing of first-degree relatives when Lp(a) is elevated. Step 4 re-estimates risk periodically and repeats a marker only when a new result could change management. Tier C information, including the history of pregnancy complications and premature menopause, may inform an individual decision in a specific context but does not by itself justify testing or a change in treatment, and Tier D use cases are reserved for research, registries and specialist settings.

### 6.4. Equity, Overdiagnosis and Threshold Choices

Precision prevention can widen disparities if markers are less accurate, less available or less often acted upon in disadvantaged groups. PRS illustrate the first problem [100,101], and the replacement of race-based eGFR equations shows that such problems can be reduced [65]. Overdiagnosis is the second concern. Each additional marker creates new categories of people labelled as being at risk, and incidental findings, often from imaging, start cascades of care that physicians report as commonly harmful [80]; overdiagnosis harms healthy people through labelling and unnecessary tests [107]. The regression to normoglycaemia observed in many older adults with prediabetes [63] is a reminder that labels carry costs as well as opportunities. Equity also depends on follow-through: a marker that is measured equally in all groups but acted upon less often in some is likely to widen gaps in outcomes, which is why D7 rates access, uptake and follow-through (equity) as well as test performance (transportability) and why even the benchmark use cases score 2 on this domain [43,54]. Finally, treatment thresholds are value judgements, not biological constants, and they are specific to the equation used: the 2025 United States hypertension guideline uses a PREVENT-estimated 10-year cardiovascular risk of 7.5% to guide drug treatment of stage 1 hypertension [53], and the 2026 dyslipidaemia guideline has also adopted PREVENT [9]. Because a triage marker can only change decisions for people near a threshold, its actionability depends on where those thresholds fall.

### 6.5. Implications for Guidelines, Decision Support and Research Reporting

For guideline developers, the proposed framework could offer a transparent way to document why a marker is considered for routine or selective use, or not at all, and which specific evidence would change that judgement. Because the domains are scored separately, disagreements between panels, for example, about the harms of CAC or the transportability of PRS, can be located precisely instead of being expressed as differences in overall judgement. For decision support in primary care, the triad suggests that electronic prompts should be conditional on context: a prompt for UACR in a person with diabetes or hypertension who has not been tested is well justified, whereas an unconditional prompt for an emerging biomarker is not. The format in which results are fed back also shapes their consequences. Communicating DNA-based risk estimates did not significantly change health behaviour [99], whereas pictorial information on carotid ultrasound findings was followed by lower LDL-C and calculated cardiovascular risk [85,86], which suggests that decision support should specify how a result is to be communicated as well as when the test is to be ordered. For researchers, the framework implies reporting standards that go beyond discrimination: reclassification within the decision zone, net benefit at clinically relevant thresholds, harms and downstream testing, and performance across sex and ancestry groups [14,16,27].

### 6.6. Illustrative Implications for Preventive Decisions

The tiers are intended to inform how a marker is used, not to prescribe treatment. Table 3 illustrates, for selected use cases, how the framework outputs might bear on decisions in practice: whether testing is routine or selective, when it is most useful, how a result might alter the choice or intensity of treatment, when it might justify further evaluation, and when repeating the test is or is not informative. Each example is tied to an action already supported by the cited guidance or trials; the framework contributes framework-informed prioritisation and an explicit link between a result and a decision, not formal clinical recommendations. The tiers refer to the scored preventive use cases: they do not replace guideline recommendations or clinical judgement for the individual patient, and the tier of a use case does not argue against a test that is clinically indicated for another purpose, such as the evaluation of symptoms. Read in this way, a higher tier signals greater confidence that acting on the result changes management and outcomes, not merely greater confidence in risk prediction. A higher tier does not, however, denote higher cardiovascular risk: the intensity of treatment and follow-up depends on the result, the person’s absolute risk and the evidence for the linked intervention. In a Tier A use case, a confirmed abnormal result is expected to trigger a guideline-supported action; in a Tier B use case, a result is most useful when a decision on initiating, deferring or intensifying treatment is pending in the context scored; and in a Tier C use case, a result is weighed only alongside other information when a decision is otherwise uncertain.

Resource-limited settings sharpen these priorities. Where budgets, laboratory capacity or imaging are constrained, the largest preventive gain is likely to come from complete application of low-cost, guideline-established use cases with high actionability, namely BP measurement with confirmation, lipid and glucose testing, eGFR with UACR in people with diabetes or hypertension, and treatment to target because treatment and control remain incomplete in many countries [6,43,54]. Use cases with lower measurement readiness or a burdensome testing pathway, such as CAC scoring, natriuretic peptide screening or polygenic scores, carry an opportunity cost: the resources they consume, including the downstream testing they can trigger, are then unavailable for closing gaps in Tier A use cases. This does not make newer markers inappropriate in such settings; a one-time Lp(a) measurement or a natriuretic peptide test in a person with diabetes may still change an individual decision when the test is affordable and the linked action is available. Local rescoring of measurement readiness, harms and burden, and transportability and equity (Section 6.1) is the mechanism by which the framework accommodates such differences, and equitable access to the actions that follow testing matters as much as access to the test itself.

## 7. Strengths, Limitations and Distinctive Contribution

Most reviews of cardiometabolic markers compare them on discrimination or reclassification. This review compares them along the whole chain from measurement to outcome and integrates evidence that is usually considered separately. Existing frameworks, including the updated American Heart Association criteria [14,16], define the dimensions needed to establish predictive and clinical utility; the contribution of the present work is to operationalise these dimensions for comparison across markers. Its distinctive elements are the marker–context–action triad as the unit of appraisal, seven explicitly scored domains, the separation of target and triage routes, gating rules that prevent a use case from reaching a higher tier on prognostic grounds alone, actionability tiers, comparison across 19 use cases and sensitivity analyses of tier assignments. By taking the triad as the unit of appraisal, the framework helps to explain why the same test can be decisive in one population and uninformative in another. By separating the target and triage routes, it clarifies the status of markers such as Lp(a) and hs-CRP, whose causal and clinical roles are frequently conflated. Contradictory findings, for example, for CAC, the ABI, triglyceride-directed therapy and diabetes screening, are made visible instead of being resolved by assertion, and the tier-migration triggers in Section 8 turn the synthesis into a research agenda. Even before validation, the framework can organise heterogeneous evidence, expose the reasoning behind each judgement, compare defined use cases, identify evidence gaps and migration triggers, and generate testable validation priorities.

The framework has several limitations. Evidence was identified through narrative, concept-driven searching, which is inherently selective and may be influenced by publication and citation bias favouring positive marker studies; the searches were not designed to be reproducible (Section 2). Each marker was scored for the context and decision with the strongest evidence, so the tiers describe selected use cases, not the markers in general. The scores are ordinal structured judgements made by the authors and were not assigned independently by two or more assessors, so inter-rater agreement was not assessed and the reliability of the scores and tier assignments is unknown; borderline domains may reasonably be scored differently by others. The construct definitions and the refined anchors were made explicit during revision, after the original scores had been assigned, and all scores were then re-assessed with knowledge of the original scores, so the reassessment was neither blinded nor independent. These limitations affect the scores and tier assignments, which may reflect the authors’ particular judgements; the evidence summarised for each use case (Table S2) and the conceptual distinctions on which the framework rests can be appraised independently of the scores. Summing ordinal scores with equal weights is a design choice that has not been shown to be optimal. The tier thresholds are heuristic, not empirically derived, and were calibrated so that undisputed benchmark factors fall in Tier A, which introduces a degree of circularity: these assignments cannot serve as validation of the thresholds, and the framework has not been externally validated or tested prospectively. Four assignments lie on tier boundaries, changing with a one-point reduction in any domain score and with a one-point rise in all thresholds, and 11 others change with a one-point change in therapeutic linkage or clinical-utility evidence (Section 5.6), so most tier assignments depend on single-point judgements in these two domains. Some overlap between domains is inherent: D4 and D5 draw on related treatment evidence for target-route markers and wherever trials enrolled people defined by the marker or its condition (for example, eGFR with UACR, T2D detection and hs-CRP), and the rules in Table S5 reduce but cannot eliminate correlated judgements; D3 also combines routes that rest on different kinds of evidence, so D3 scores are not strictly comparable across routes. Actionability depends on the health system, so scores for measurement readiness and burden may not transfer between settings. The underlying evidence is dominated by cohorts of European ancestry from high-income countries; heterogeneity of assays, units and acquisition protocols, particularly for Lp(a), natriuretic peptides and CAC, limits comparability; and the equity subcomponent of D7 was rated from limited direct evidence on access and uptake and received the same rating for every use case, so it did not discriminate between them. Cost-effectiveness, which varies between health systems, was considered only indirectly through measurement readiness and burden. Finally, the evidence base is changing quickly: guidelines are adopting new risk equations, and outcome trials of Lp(a)-lowering therapies are in progress or awaiting peer-reviewed publication, so several scores will need revision as peer-reviewed data appear.

## 8. Future Directions

The framework generates specific, testable priorities (Table 4). For the framework itself, an appropriate next step is to assess the reliability of the scores: assessors not involved in its development could apply the anchors and rules in Table 1 and Table S5 to the same use cases, and their agreement on domain scores and tiers could be quantified with statistics suited to ordinal ratings. Consensus methods could subsequently refine the anchors, weights and thresholds, and validation against criteria independent of those used to set the thresholds, such as the judgements of guideline panels not involved in development or the results of marker-guided trials, should precede any clinical use. The applicability of the framework across clinical and health-system settings and the effect of framework-informed pathways on care and outcomes (Section 6.6) also remain to be examined. Validation should include populations of diverse ancestry and socioeconomic position, and economic evaluations that model the full testing pathway, including downstream investigations triggered by incidental findings, are needed for the Tier B imaging use cases. For individual use cases, the evidence most likely to change tiers is identifiable. Peer-reviewed outcome data from trials of Lp(a)-lowering therapies will determine whether Lp(a) becomes a therapeutic target, which would raise its therapeutic linkage and clinical-utility scores, or remains a triage marker. A randomised trial of CAC-guided versus equation-guided statin allocation with hard outcomes, in people near treatment thresholds, would resolve the principal uncertainty for CAC. Large pragmatic trials of natriuretic peptide-guided prevention are needed to confirm PONTIAC and STOP-HF. For PRS, a move beyond Tier C would require a two-point gain in its limiting domains (Table 4), for example, from hard-endpoint trials of PRS-informed treatment in populations of diverse ancestry together with multi-ancestry scores of equivalent performance. For the highest-tier use cases, the priority is implementation research, such as electronic prompts and pharmacist-led or nurse-led pathways, to close the gaps in albuminuria testing, BP confirmation and FH detection. Across all markers, assays should be harmonised, with Lp(a) reported in molar units, and validation studies should be powered to detect differences in performance across sex, ancestry and socioeconomic groups instead of reporting pooled estimates alone.

Future applications could also extend the framework to comorbid conditions that cluster with cardiometabolic risk, such as COPD and OSA, provided that each is defined as a use case by the population and the specific preventive decision its identification is intended to inform. In a meta-analysis of 29 datasets, people with COPD were more likely than those without to have a diagnosis of cardiovascular disease (odds ratio 2.46; 95% CI 2.02–3.00) and more often had hypertension and diabetes, but they were also far more likely to have ever smoked [108], an exposure common to both conditions. This co-occurrence does not show that identifying COPD changes a specific cardiometabolic preventive decision beyond the assessment of smoking and conventional risk factors, and we did not identify studies that tested this question directly. OSA is associated with cardiovascular and cerebrovascular disease and T2D [109], but in SAVE, continuous positive airway pressure (CPAP) did not reduce cardiovascular events among 2717 adults with moderate-to-severe OSA and established cardiovascular disease (HR 1.10; 95% CI 0.91–1.32), although it reduced snoring and daytime sleepiness and improved health-related quality of life and mood [110]. In a meta-analysis of 10 trials in adults with sleep apnoea, positive airway pressure was not associated with fewer major adverse cardiovascular events (relative risk 0.77; 95% CI 0.53–1.13) [111], and the United States Preventive Services Task Force judged the evidence insufficient to assess the balance of benefits and harms of screening for OSA in asymptomatic adults or those with unrecognised symptoms [109]. Because both conditions are diagnosed and managed in their own right, and because evidence that their detection improves cardiometabolic outcomes is currently lacking, they were not scored in this review.

## 9. Conclusions

In the use cases evaluated, cardiometabolic risk markers differed less in their capacity to predict than in their capacity to change what clinicians do, and it is the latter that determines their value in primary care. The proposed semi-quantitative framework structures the appraisal of actionability for defined marker–context–action use cases, distinguishes prediction from clinical consequence and makes the reasoning behind each judgement open to challenge. Applied by the authors to 19 use cases, it provisionally placed the use cases of established and mostly inexpensive measures in the highest tier: LDL-C, BP, detection of T2D, kidney function with albuminuria and detection of FH. These are nonetheless incompletely implemented. The evaluated use cases of CAC, apoB, natriuretic peptides, FIB-4, hs-CRP, prediabetes detection, Lp(a), carotid plaque and the ABI were assigned to Tier B, the last three on the boundary with Tier C: their results are most useful when they can alter a specific decision. The use cases of triglyceride-directed drug therapy, a history of pregnancy complications and premature menopause, and PRS were placed in Tier C: these results may inform individual decisions but do not by themselves justify testing or treatment. Clonal haematopoiesis and proteomic scores remain research tools. The tier assignments are provisional outputs of a framework that has not been validated, not clinical recommendations; four of them change with a one-point reduction in any domain score and most of the others with a one-point change in therapeutic linkage or clinical-utility evidence. The most consequential uncertainties are whether lowering Lp(a) reduces events, whether CAC-guided care improves hard outcomes, and whether the prognostic value of natriuretic peptides and PRS can be translated into better outcomes at scale. The framework identifies these evidence gaps and may inform future research, guideline development and implementation; until they are closed, precision prevention in primary care is best advanced by acting fully on the use cases already known to be actionable and adding new markers only where their results can change decisions.

## Figures and Tables

**Table 1:** Domains, guiding questions and scoring anchors of the proposed semi-quantitative clinical actionability framework.

Domain	Guiding Question	Score 0	Score 1	Score 2	Score 3
D1 Measurement readiness	Can the marker be measured reliably, affordably and promptly in primary care?	Research assay only or unavailable	Specialised laboratory or imaging; incomplete standardisation, limited availability or high test cost	Widely available and standardised, with cost, referral, unit or assay caveats	Routine, inexpensive and standardised, with established thresholds
D2 Prognostic validity	Is the marker independently and consistently associated with hard cardiometabolic outcomes?	Inconsistent or null association	Association limited to selected, cross-sectional or retrospective data, or not independent of established markers	Independent association in large prospective cohorts	Strong, graded association across large prospective cohorts and diverse populations, including individual-participant meta-analyses
D3 Decision-relevant information	Does the result inform the decision at hand, through route I (incremental), C (constituent) or S (state-defining)?	None	Route I: statistically significant but clinically marginal increment (e.g., ΔC < 0.01, little reclassification near the threshold)	Route I: meaningful reclassification or rule-out in the decision zone	Route I: substantial reclassification near the threshold, including de-risking; route C: core constituent of guideline-recommended risk equations; or route S: defines a clinically recognised high-risk state on its own
D4 Therapeutic linkage	Does an actionable pathway exist, and how directly does the result trigger or modify an intervention of proven efficacy?	No intervention depends on the result	Generic advice only, or efficacy of the linked intervention not established in the marker-defined group	Indirect pathway: the result shifts the expected absolute benefit of a proven intervention (triage route), or the marker is a target with efficacy shown on surrogate outcomes only	Direct pathway: the marker is an established target, or defines a condition, for which the linked intervention reduces hard outcomes in RCTs
D5 Clinical-utility evidence	Is there direct evidence that care informed by the marker, or directed at the population or condition it defines, improves outcomes?	None, or consistent evidence of no benefit	Observational or modelling evidence only, trials showing changes in behaviour or process only, or conflicting randomised evidence	RCT evidence of improved validated intermediate outcomes, or of hard-outcome benefit in a population selected by a triage marker without a marker-guided comparator	RCT evidence of hard-outcome benefit from marker-guided care, or from treatment directed at the marker or at the condition it defines
D6 Harms and burden of the testing pathway (reverse-scored)	What harms and burdens do measurement, confirmation and downstream investigation impose on the person tested (excluding test cost and treatment effects)?	Substantial (radiation, invasive cascades, overdiagnosis) without mitigation	Moderate (e.g., radiation or frequent incidental findings with downstream testing)	Minor and manageable (false positives needing repeat testing, labelling)	Negligible
D7 Transportability (T) and equity (E)	T: is performance maintained across sex, age, ancestry and socioeconomic groups and settings? E: are access, uptake and follow-through likely to be equitable? T and E are rated separately; the lower rating is scored	T: poor transportability; E: high risk of widening disparities	T: substantially reduced validity in major population groups; E: major access or uptake barriers	T or E: recognised limitations that can be mitigated, or consistency or equity not affirmatively shown	T: affirmative evidence of consistent performance across groups; E: affirmative evidence of equitable access and uptake

Each domain is scored 0–3 on an ordinal scale (total 0–21); the levels are categories defined by the anchors, not interval measurements, and the score assigned is the highest level whose anchor is met. Hard cardiometabolic outcomes: cardiovascular events, kidney failure, severe liver disease and death. D3 routes: I, incremental information beyond baseline assessment; C, core constituent of guideline-recommended risk equations; S, direct identification of a clinically recognised high-risk state; the route credited is recorded for each use case in Table S1. D5 reading rules: conflicting randomised evidence at a given level does not support that level; consistent evidence of no benefit scores 0; when a randomised trial of the testing strategy did not improve hard outcomes, D5 is at most 2. Validated intermediate outcomes are measured risk factors or disease states with an established relation to hard outcomes (e.g., LDL-C, BP, incident T2D); calculated risk scores are not counted as such. D6 excludes the cost and availability of the test (scored in D1), inequity of access (scored in D7) and adverse effects of treatment, which are reflected in D4 and D5. D7: transportability (T) and equity (E) are rated separately on these anchors, and the lower rating is scored. Construct definitions and rules preventing double counting are given in Table S5. Tier A (routine actionability): total ≥ 17 with D4 = 3 and D5 ≥ 2 (second gating rule). Tier B (selective actionability): D4 ≥ 2 and a total of 13–16, or of ≥17 without meeting Tier A criteria. Tier C (contextual actionability): total 9–12, or any total >8 with D4 ≤ 1 (first gating rule). Tier D (research stage): total ≤8. Tiers are provisional outputs of the proposed framework. ΔC, change in C-statistic; RCT, randomised controlled trial.

**Table 2:** Application of the proposed framework to 19 marker–context–action use cases in primary care: domain scores and provisional tiers.

No.	Marker	Context	Decision Informed	D1	D2	D3	D4	D5	D6	D7	Total	Tier
Established causal risk factors												
1	LDL-C/non-HDL-C	All adults (40–75 years; lifetime perspective)	Initiate or intensify lipid-lowering therapy	3	3	3	3	3	3	2	20	A
2	Apolipoprotein B	Hypertriglyceridaemia, diabetes, obesity or low LDL-C (discordance)	Refine atherogenic burden and treatment intensity	2	3	2	3	1	3	2	16	B
3	Familial hypercholesterolaemia case-finding	LDL-C ≥ 190 mg/dL (4.9 mmol/L), clinical criteria, child–parent or cascade screening	Early high-intensity LDL-C lowering; cascade testing	2	3	3	3	2	2	2	17	A
4	Blood pressure (office, with out-of-office confirmation)	All adults	Diagnose hypertension; initiate or intensify treatment	3	3	3	3	3	2	2	19	A
5	HbA1c or fasting glucose: type 2 diabetes	Adults with overweight, obesity or other risk factors	Diagnose T2D; glucose-lowering, lipid-lowering and organ-protective therapy	3	3	3	3	2	2	2	18	A
6	HbA1c or fasting glucose: prediabetes	Adults with overweight, obesity or other risk factors	Identify prediabetes; structured lifestyle programme	3	2	1	2	2	2	2	14	B
Markers of cardiometabolic organ involvement												
7	eGFR plus UACR	Adults with diabetes and/or hypertension	Confirm and stage CKD; consider guideline-indicated RAS blockade, SGLT2 inhibitor and, in T2D, finerenone; intensify risk management	3	3	3	3	3	2	2	19	A
8	Natriuretic peptides (BNP or NT-proBNP)	Adults with T2D or other cardiovascular risk factors, without HF	Identify stage B HF; echocardiography, intensified cardioprotective therapy	2	3	2	2	2	2	2	15	B
9	FIB-4 index	Adults with T2D, obesity or ≥2 metabolic risk factors	Rule out advanced fibrosis; second-line test or referral	3	2	2	2	1	2	2	14	B
Circulating risk enhancers												
10	Lipoprotein(a)	Adults (once in adulthood)	Intensify LDL-C lowering and risk-factor control; cascade testing	2	3	1	2	1	2	2	13	B
11	hs-CRP	Adults at borderline or intermediate risk	Risk enhancer informing statin initiation	3	2	1	2	2	2	2	14	B
12	Triglycerides	Adults with triglycerides 150–499 mg/dL (1.7–5.6 mmol/L)	Initiate triglyceride-directed drug therapy	3	1	0	1	1	2	2	10	C
Subclinical atherosclerosis imaging												
13	Coronary artery calcium score	Adults aged 40–75 years at borderline or intermediate risk with an uncertain statin decision	Initiate, intensify or defer statin therapy	2	3	3	2	2	1	2	15	B
14	Carotid plaque ultrasound	Adults at moderate risk	Reclassify risk; communicate subclinical disease	2	2	1	2	2	2	2	13	B
15	Ankle–brachial index	Asymptomatic adults (screening)	Identify asymptomatic PAD; initiate or intensify statin therapy	2	3	1	2	1	2	2	13	B
Life-course, genomic and multi-omic markers												
16	Pregnancy complications and premature menopause	Women (history-taking)	Earlier, more frequent risk-factor surveillance	3	2	1	1	1	3	2	13	C
17	Polygenic risk score for coronary artery disease	Adults at intermediate risk or younger adults	Statin or lifestyle intensification	1	2	1	2	2	2	1	11	C
18	Clonal haematopoiesis of indeterminate potential	Adults (sequencing)	No established preventive action	1	2	0	0	0	1	2	6	D
19	Large-scale proteomic risk score	Adults (research platforms)	No established preventive action	0	2	1	0	0	2	2	7	D

Scores are ordinal structured judgements made by the authors, based on the evidence summarised in Section 5; the rationale for each score, the D3 route, the D7 subcomponent ratings and the supporting references are given in Table S1. Tiers describe the evaluated use cases, not intrinsic properties of the markers, and are provisional outputs of a framework that has not been validated; totals are organising summaries, not measurements. Six scores were changed in the reassessment performed during revision (use case 10, D3, from 2 to 1; use case 12, D3, from 1 to 0; use case 15, D2, from 2 to 3; use case 18, D3, from 1 to 0; use cases 18 and 19, D7, from 1 to 2). D1–D7, domains defined in Table 1 (D6 reverse-scored). Tiers: A, routine actionability; B, selective actionability; C, contextual actionability; D, research stage. BNP, B-type natriuretic peptide; CKD, chronic kidney disease; eGFR, estimated glomerular filtration rate; FIB-4, Fibrosis-4 index; HbA1c, glycated haemoglobin; HF, heart failure; hs-CRP, high-sensitivity C-reactive protein; LDL-C, low-density lipoprotein cholesterol; non-HDL-C, non-high-density lipoprotein cholesterol; NT-proBNP, N-terminal pro-B-type natriuretic peptide; PAD, peripheral artery disease; RAS, renin–angiotensin system; SGLT2, sodium–glucose cotransporter 2; T2D, type 2 diabetes; UACR, urinary albumin-to-creatinine ratio.

**Table 3:** Illustrative implications of selected use cases for preventive decisions in primary care (framework-informed prioritisation, not formal recommendations).

Use Case (Provisional Tier)	Testing Approach Suggested by the Tier	How the Result Can Change Management	Further Evaluation and Repeat Testing	Basis
eGFR plus UACR in diabetes or hypertension (A)	Routine in the scored context	Confirmed albuminuric CKD prompts assessment for guideline-indicated RAS blockade and SGLT2 inhibition; in T2D with albuminuria persisting despite maximally tolerated RAS blockade, finerenone may be added when eGFR and serum potassium permit; cardiovascular risk management is intensified	Confirm an elevated UACR on a first morning sample; establish chronicity (≥3 months) from previous results, imaging or clinical history, or by repeat testing if these are lacking; check potassium and creatinine after starting RAS blockade or finerenone; monitoring frequency follows CKD risk category	[2,32,35,36]
BP with out-of-office confirmation (A)	Routine	Confirmed hypertension prompts treatment, with intensity guided by targets and, in the United States, by PREVENT-estimated risk in stage 1 hypertension	Confirm elevated office values out of office before diagnosis; repeat to monitor control	[50,52,53]
CAC score, 40–75 years, uncertain statin decision (B)	Selective: when the statin decision is uncertain near a threshold	A score of 0 may support deferral with later reassessment; detectable CAC favours statin therapy, with lower LDL-C goals at higher scores	Not used for monitoring; repeat only if a new result could change a deferred decision; incidental findings need a defined pathway	[8,9,40,80]
Lp(a), once in adulthood (B, boundary)	Measure once; act selectively	An elevated value favours earlier and more intensive LDL-C lowering and stricter control of other risk factors; no Lp(a)-specific therapy is established	Repeat measurement is rarely informative because concentration is largely genetically determined; cascade testing of first-degree relatives	[8,9,33]
hs-CRP, borderline or intermediate risk (B)	Selective: when the statin decision is uncertain	A persistently elevated value (>2 mg/L) favours statin initiation	Repeat after intercurrent illness to confirm persistence; not used to monitor treatment	[8,18]
Natriuretic peptides, T2D or other cardiovascular risk factors (B)	Selective, context-specific	An elevated value prompts echocardiography and may justify intensified cardioprotective therapy	Optimal interval for repeat testing not established by outcome trials	[37,38,67]
ABI, asymptomatic adults (B, boundary)	Selective: when a statin decision is uncertain	A value of 0.90 or less identifies PAD, in which statin therapy has larger expected absolute benefit; after clinical assessment, single antiplatelet therapy is reasonable in asymptomatic PAD (expert opinion), taking bleeding risk into account	Values above 1.40 indicate poorly compressible arteries and call for an alternative test such as the toe–brachial index	[23,87,89,90,92]
Pregnancy complications and premature menopause (C)	Collected in routine history-taking; no additional testing	May act as a risk enhancer when a treatment decision is otherwise uncertain	Earlier and more regular measurement of BP, glucose and lipids	[8,104,105]
CAD polygenic score (C)	Not for routine testing; an existing result may be weighed when a decision is otherwise uncertain	May inform an individual decision near a treatment threshold	Not repeated (germline); performance is lower outside European ancestry	[100,101]

Illustrative examples derived from the provisional tiers and the evidence in Section 5; they are not formal clinical recommendations or a treatment algorithm, and local guidance, resources and patient preferences determine how the cited actions are applied. Tiers: A, routine actionability; B, selective actionability; C, contextual actionability. ABI, ankle–brachial index; BP, blood pressure; CAC, coronary artery calcium; CAD, coronary artery disease; CKD, chronic kidney disease; eGFR, estimated glomerular filtration rate; hs-CRP, high-sensitivity C-reactive protein; LDL-C, low-density lipoprotein cholesterol; Lp(a), lipoprotein(a); PAD, peripheral artery disease; PREVENT, Predicting Risk of cardiovascular disease EVENTs; RAS, renin–angiotensin system; SGLT2, sodium–glucose cotransporter 2; T2D, type 2 diabetes; UACR, urinary albumin-to-creatinine ratio.

**Table 4:** Evidence that would move selected use cases between tiers (tier-migration triggers).

Use Case (Current Score, Tier)	Limiting Domain(s)	Evidence that Would Change the Tier	Expected Consequence
Lp(a), once in adulthood (13, B)	D3, D4, D5	Peer-reviewed phase 3 trials showing that Lp(a)-lowering therapy reduces hard outcomes, including in primary prevention	Target route established: D4 3 and D5 3 (total 16, Tier B); Tier A would also require one further point in another domain (e.g., D3 ≥2). Negative trials would confirm triage-only use
CAC score, borderline/intermediate risk (15, B)	D4, D5, D6	RCT of CAC-guided versus equation-guided treatment with hard endpoints; low-dose acquisition and standard management of incidental findings	D4 3 and D5 3 (D6 2 with mitigation) → Tier A
ApoB, discordance contexts (16, B)	D5	RCT comparing apoB-guided with LDL-C-guided treatment	D5 ≥ 2 → Tier A
Natriuretic peptides, T2D or other cardiovascular risk factors (15, B)	D4, D5	Large pragmatic RCT of natriuretic peptide-guided prevention with hard endpoints	D4 3, D5 3 → Tier A
hs-CRP, borderline/intermediate risk (14, B)	D3, D4	Outcome trials of anti-inflammatory therapy in primary prevention; standardised repeat measurement	Target route for inflammation (D4 3, with D5 3 if trials select by hs-CRP); total 15–16, remaining Tier B
FIB-4, T2D or obesity (14, B)	D4, D5	Randomised trials showing that FIB-4-based pathways reduce liver or cardiovascular events	D5 3 (total 16, Tier B); Tier A would also require a linked therapy with hard-outcome benefit (D4 3; total 17)
Prediabetes, overweight or obesity (14, B)	D3, D4, D5	RCTs showing that structured programmes for screen-detected prediabetes reduce cardiovascular events	D4 3 and D5 3 (total 16): firmer Tier B; Tier A would also require one further point in another domain (e.g., D3 ≥ 2)
Pregnancy complications and premature menopause (13, C)	D4, D5	Evidence that the history identifies women with larger expected benefit from a proven intervention; trials of structured postpartum or mid-life risk-factor programmes with long-term outcomes	D4 2 → Tier B
CAD polygenic score (11, C)	D1, D3, D5, D7	Hard-outcome RCTs of PRS-informed treatment; multi-ancestry scores with equivalent performance; standardised clinical reporting	Tier B
Triglycerides, triglyceride-directed drug therapy (10, C)	D2, D3, D4, D5	Trial showing benefit of triglyceride lowering independent of apoB reduction, with an inert comparator, including primary prevention	Target route established: D4 3 and D5 ≥ 2 (total ≥ 13) → Tier B
ABI, asymptomatic adults (13, B)	D3, D5	RCT of ABI-guided statin therapy in asymptomatic adults with hard outcomes; evidence of meaningful reclassification in intermediate-risk adults	D5 ≥ 2 or D3 ≥ 2 (total ≥ 14): Tier B, no longer on the boundary with Tier C
CHIP (6, D)	D1, D3, D4, D5	Standardised clinical assay; RCT of targeted therapy in carriers	Tier C or B
Proteomic risk score (7, D)	D1, D4, D5	Clinically validated, standardised assay; demonstration that results change management and outcomes	Tier C
Tier A use cases (e.g., eGFR + UACR, 19, A)	Implementation	Implementation trials (electronic prompts, team-based pathways) that close testing and treatment gaps	No tier change; larger population benefit

ABI, ankle–brachial index; apoB, apolipoprotein B; CAC, coronary artery calcium; CAD, coronary artery disease; CHIP, clonal haematopoiesis of indeterminate potential; D1–D7, domains defined in Table 1; eGFR, estimated glomerular filtration rate; FIB-4, Fibrosis-4 index; hs-CRP, high-sensitivity C-reactive protein; LDL-C, low-density lipoprotein cholesterol; Lp(a), lipoprotein(a); PRS, polygenic risk score; RCT, randomised controlled trial; T2D, type 2 diabetes; UACR, urinary albumin-to-creatinine ratio.

## Data Availability

No new primary empirical data were collected for this narrative review. The domain scores and sensitivity analyses developed by the authors are presented in the manuscript and its Supplementary Materials.

## Supplementary Materials

The following supporting information can be downloaded at https://www.mdpi.com/article/10.3390/jcm15218706/s1. Table S1: Domain-level scoring rationale, D3 routes and D7 subcomponent ratings for the 19 marker–context–action use cases; Table S2: Quantitative results of studies informing the domain scores; Table S3: Self-assessment against the Scale for the Assessment of Narrative Review Articles (SANRA); Table S4A: Sensitivity of tier assignments to single-domain score perturbations; Table S4B: Additional sensitivity analyses of tier assignments performed during revision of the manuscript; Table S5: Construct definitions of the seven domains and rules preventing double counting.

## Abbreviations

The following abbreviations are used in this manuscript:

AAA	Aspirin for Asymptomatic Atherosclerosis
ABI	ankle–brachial index
apoB	apolipoprotein B
ARIC	Atherosclerosis Risk in Communities
ASCVD	atherosclerotic cardiovascular disease
BNP	B-type natriuretic peptide
BP	blood pressure
CAC	coronary artery calcium
CAD	coronary artery disease
CHIP	clonal haematopoiesis of indeterminate potential
CI	confidence interval
CKD	chronic kidney disease
CKM	cardiovascular–kidney–metabolic
COPD	chronic obstructive pulmonary disease
CPAP	continuous positive airway pressure
CRP	C-reactive protein
eGFR	estimated glomerular filtration rate
FH	familial hypercholesterolaemia
FIB-4	Fibrosis-4 index
FPG	fasting plasma glucose
HbA1c	glycated haemoglobin
HDL	high-density lipoprotein
HF	heart failure
HR	hazard ratio
hs-CRP	high-sensitivity C-reactive protein
LDL	low-density lipoprotein
LDL-C	low-density lipoprotein cholesterol
Lp(a)	lipoprotein(a)
MASLD	metabolic dysfunction-associated steatotic liver disease
MESA	Multi-Ethnic Study of Atherosclerosis
non-HDL(-C)	non-high-density lipoprotein (cholesterol)
NT-proBNP	N-terminal pro-B-type natriuretic peptide
OSA	obstructive sleep apnoea
PAD	peripheral artery disease
PREVENT	Predicting Risk of cardiovascular disease EVENTs
PRS	polygenic risk score(s)
RAS	renin–angiotensin system
RCT	randomised controlled trial
SANRA	Scale for the Assessment of Narrative Review Articles
SCORE	Systematic Coronary Risk Evaluation
SCORE2	Systematic Coronary Risk Evaluation 2
SGLT2	sodium–glucose cotransporter 2
T2D	type 2 diabetes
UACR	urinary albumin-to-creatinine ratio

## References

[1] Ndumele C.E., Rangaswami J., Chow S.L., Neeland I.J., Tuttle K.R., Khan S.S., Coresh J., Mathew R.O., Baker-Smith C.M., Carnethon M.R. (2023). Cardiovascular-Kidney-Metabolic Health: A Presidential Advisory from the American Heart Association. Circulation.

[2] Ndumele C.E., Rodriguez F., Dixon D.L., Khan S.S., Mukherjee D., Bajaj M., Bangalore S., Bozkurt B., Breathett K., Clarke S.L. (2026). 2026 AHA/ACC/ADA/ASN Guideline for the Prevention, Detection, Evaluation, and Management of Cardiovascular-Kidney-Metabolic Syndrome. J. Am. Coll. Cardiol..

[3] European Association for the Study of the Liver (EASL), European Association for the Study of Diabetes (EASD), European Association for the Study of Obesity (EASO) (2024). EASL–EASD–EASO Clinical Practice Guidelines on the management of metabolic dysfunction-associated steatotic liver disease (MASLD). J. Hepatol..

[4] Mantovani A., Csermely A., Petracca G., Beatrice G., Corey K.E., Simon T.G., Byrne C.D., Targher G. (2021). Non-alcoholic fatty liver disease and risk of fatal and non-fatal cardiovascular events: An updated systematic review and meta-analysis. Lancet Gastroenterol. Hepatol..

[5] Mensah G.A., Fuster V., Murray C.J.L., Roth G.A., Global Burden of Cardiovascular Diseases and Risks Collaborators (2023). Global Burden of Cardiovascular Diseases and Risks, 1990–2022. J. Am. Coll. Cardiol..

[6] NCD Risk Factor Collaboration (NCD-RisC) (2024). Worldwide trends in diabetes prevalence and treatment from 1990 to 2022: A pooled analysis of 1108 population-representative studies with 141 million participants. Lancet.

[7] Damen J.A.A.G., Hooft L., Schuit E., Debray T.P.A., Collins G.S., Tzoulaki I., Lassale C.M., Siontis G.C.M., Chiocchia V., Roberts C. (2016). Prediction models for cardiovascular disease risk in the general population: Systematic review. BMJ.

[8] Mach F., Koskinas K.C., Roeters van Lennep J.E., Tokgözoğlu L., Badimon L., Baigent C., Benn M., Binder C.J., Catapano A.L., De Backer G.G. (2025). 2025 Focused Update of the 2019 ESC/EAS Guidelines for the management of dyslipidaemias. Eur. Heart J..

[9] Blumenthal R.S., Morris P.B., Gaudino M., Johnson H.M., Anderson T.S., Bittner V.A., Blankstein R., Brewer L.C., Cho L., de Ferranti S.D. (2026). 2026 ACC/AHA/AACVPR/ABC/ACPM/ADA/AGS/APhA/ASPC/NLA/PCNA Guideline on the Management of Dyslipidemia. J. Am. Coll. Cardiol..

[10] Khan S.S., Matsushita K., Sang Y., Ballew S.H., Grams M.E., Surapaneni A., Blaha M.J., Carson A.P., Chang A.R., Ciemins E. (2024). Development and Validation of the American Heart Association’s PREVENT Equations. Circulation.

[11] Kotseva K., De Backer G., De Bacquer D., Rydén L., Hoes A., Grobbee D., Maggioni A., Marques-Vidal P., Jennings C., Abreu A. (2021). Primary prevention efforts are poorly developed in people at high cardiovascular risk: A report from the European Society of Cardiology EURObservational Research Programme EUROASPIRE V survey in 16 European countries. Eur. J. Prev. Cardiol..

[12] Shin J.-I., Chang A.R., Grams M.E., Coresh J., Ballew S.H., Surapaneni A., Matsushita K., Bilo H.J.G., Carrero J.J., Chodick G. (2021). Albuminuria Testing in Hypertension and Diabetes: An Individual-Participant Data Meta-Analysis in a Global Consortium. Hypertension.

[13] SCORE2 Working Group and ESC Cardiovascular Risk Collaboration (2021). SCORE2 risk prediction algorithms: New models to estimate 10-year risk of cardiovascular disease in Europe. Eur. Heart J..

[14] Hlatky M.A., Greenland P., Arnett D.K., Ballantyne C.M., Criqui M.H., Elkind M.S.V., Go A.S., Harrell F.E., Hong Y., Howard B.V. (2009). Criteria for evaluation of novel markers of cardiovascular risk: A scientific statement from the American Heart Association. Circulation.

[15] Fryback D.G., Thornbury J.R. (1991). The efficacy of diagnostic imaging. Med. Decis. Mak..

[16] Khan S.S., Greenland P., Hayman L.L., Khera R., Navar A.M., Pencina M.J., Reza N., Shah S.H., Shanbhag S., Weber B. (2026). Criteria to Assess the Predictive and Clinical Utility of Novel Models, Biomarkers, and Tools for Risk of Cardiovascular Disease: A Scientific Statement from the American Heart Association. Circulation.

[17] Krogsbøll L.T., Jørgensen K.J., Gøtzsche P.C. (2019). General health checks in adults for reducing morbidity and mortality from disease. Cochrane Database Syst. Rev..

[18] Ridker P.M., Danielson E., Fonseca F.A.H., Genest J., Gotto A.M., Kastelein J.J.P., Koenig W., Libby P., Lorenzatti A.J., MacFadyen J.G. (2008). Rosuvastatin to prevent vascular events in men and women with elevated C-reactive protein. N. Engl. J. Med..

[19] Berg J.S., Foreman A.K.M., O’Daniel J.M., Booker J.K., Boshe L., Carey T., Crooks K.R., Jensen B.C., Juengst E.T., Lee K. (2016). A semiquantitative metric for evaluating clinical actionability of incidental or secondary findings from genome-scale sequencing. Genet. Med..

[20] Baethge C., Goldbeck-Wood S., Mertens S. (2019). SANRA—A scale for the quality assessment of narrative review articles. Res. Integr. Peer Rev..

[21] Emerging Risk Factors Collaboration (2011). Separate and combined associations of body-mass index and abdominal adiposity with cardiovascular disease: Collaborative analysis of 58 prospective studies. Lancet.

[22] Ference B.A., Ginsberg H.N., Graham I., Ray K.K., Packard C.J., Bruckert E., Hegele R.A., Krauss R.M., Raal F.J., Schunkert H. (2017). Low-density lipoproteins cause atherosclerotic cardiovascular disease. 1. Evidence from genetic, epidemiologic, and clinical studies. A consensus statement from the European Atherosclerosis Society Consensus Panel. Eur. Heart J..

[23] Cholesterol Treatment Trialists’ (CTT) Collaborators (2012). The effects of lowering LDL cholesterol with statin therapy in people at low risk of vascular disease: Meta-analysis of individual data from 27 randomised trials. Lancet.

[24] C Reactive Protein Coronary Heart Disease Genetics Collaboration (CCGC) (2011). Association between C reactive protein and coronary heart disease: Mendelian randomisation analysis based on individual participant data. BMJ.

[25] Kamstrup P.R., Tybjaerg-Hansen A., Steffensen R., Nordestgaard B.G. (2009). Genetically elevated lipoprotein(a) and increased risk of myocardial infarction. JAMA.

[26] Detrano R., Guerci A.D., Carr J.J., Bild D.E., Burke G., Folsom A.R., Liu K., Shea S., Szklo M., Bluemke D.A. (2008). Coronary calcium as a predictor of coronary events in four racial or ethnic groups. N. Engl. J. Med..

[27] Vickers A.J., Elkin E.B. (2006). Decision curve analysis: A novel method for evaluating prediction models. Med. Decis. Mak..

[28] Rose G. (1985). Sick individuals and sick populations. Int. J. Epidemiol..

[29] Visseren F.L.J., Mach F., Smulders Y.M., Carballo D., Koskinas K.C., Bäck M., Benetos A., Biffi A., Boavida J.-M., Capodanno D. (2021). 2021 ESC Guidelines on cardiovascular disease prevention in clinical practice. Eur. Heart J..

[30] Thanassoulis G., Williams K., Altobelli K.K., Pencina M.J., Cannon C.P., Sniderman A.D. (2016). Individualized Statin Benefit for Determining Statin Eligibility in the Primary Prevention of Cardiovascular Disease. Circulation.

[31] Khan S.S., Ndumele C.E., Bhave N., Blumenthal R.S., Coresh J., Huang X., Joseph J.J., Khera A., Ho J.E., Lloyd-Jones D.M. (2026). Use of Predicted Risk and Expected Benefit to Guide Decision-Making in Cardiovascular-Kidney-Metabolic Syndrome for the Primary Prevention of Cardiovascular Disease: A Scientific Statement from the American Heart Association and American College of Cardiology. J. Am. Coll. Cardiol..

[32] Kidney Disease: Improving Global Outcomes (KDIGO) CKD Work Group (2024). KDIGO 2024 Clinical Practice Guideline for the Evaluation and Management of Chronic Kidney Disease. Kidney Int..

[33] Kronenberg F., Mora S., Stroes E.S.G., Ference B.A., Arsenault B.J., Berglund L., Dweck M.R., Koschinsky M., Lambert G., Mach F. (2022). Lipoprotein(a) in atherosclerotic cardiovascular disease and aortic stenosis: A European Atherosclerosis Society consensus statement. Eur. Heart J..

[34] Staplin N., de la Sierra A., Ruilope L.M., Emberson J.R., Vinyoles E., Gorostidi M., Ruiz-Hurtado G., Segura J., Baigent C., Williams B. (2023). Relationship between clinic and ambulatory blood pressure and mortality: An observational cohort study in 59 124 patients. Lancet.

[35] Heerspink H.J.L., Stefánsson B.V., Correa-Rotter R., Chertow G.M., Greene T., Hou F.-F., Mann J.F.E., McMurray J.J.V., Lindberg M., Rossing P. (2020). Dapagliflozin in Patients with Chronic Kidney Disease. N. Engl. J. Med..

[36] Agarwal R., Filippatos G., Pitt B., Anker S.D., Rossing P., Joseph A., Kolkhof P., Nowack C., Gebel M., Ruilope L.M. (2022). Cardiovascular and kidney outcomes with finerenone in patients with type 2 diabetes and chronic kidney disease: The FIDELITY pooled analysis. Eur. Heart J..

[37] Huelsmann M., Neuhold S., Resl M., Strunk G., Brath H., Francesconi C., Adlbrecht C., Prager R., Luger A., Pacher R. (2013). PONTIAC (NT-proBNP selected prevention of cardiac events in a population of diabetic patients without a history of cardiac disease): A prospective randomized controlled trial. J. Am. Coll. Cardiol..

[38] Ledwidge M., Gallagher J., Conlon C., Tallon E., O’Connell E., Dawkins I., Watson C., O’Hanlon R., Bermingham M., Patle A. (2013). Natriuretic peptide-based screening and collaborative care for heart failure: The STOP-HF randomized trial. JAMA.

[39] Yeboah J., McClelland R.L., Polonsky T.S., Burke G.L., Sibley C.T., O’Leary D., Carr J.J., Goff D.C., Greenland P., Herrington D.M. (2012). Comparison of novel risk markers for improvement in cardiovascular risk assessment in intermediate-risk individuals. JAMA.

[40] Nasir K., Bittencourt M.S., Blaha M.J., Blankstein R., Agatston A.S., Rivera J.J., Miedema M.D., Sibley C.T., Shaw L.J., Blumenthal R.S. (2015). Implications of Coronary Artery Calcium Testing Among Statin Candidates According to American College of Cardiology/American Heart Association Cholesterol Management Guidelines: MESA (Multi-Ethnic Study of Atherosclerosis). J. Am. Coll. Cardiol..

[41] Rozanski A., Gransar H., Shaw L.J., Kim J., Miranda-Peats L., Wong N.D., Rana J.S., Orakzai R., Hayes S.W., Friedman J.D. (2011). Impact of coronary artery calcium scanning on coronary risk factors and downstream testing: The EISNER (Early Identification of Subclinical Atherosclerosis by Noninvasive Imaging Research) prospective randomized trial. J. Am. Coll. Cardiol..

[42] Lindholt J.S., Søgaard R., Rasmussen L.M., Mejldal A., Lambrechtsen J., Steffensen F.H., Frost L., Egstrup K., Urbonaviciene G., Busk M. (2022). Five-Year Outcomes of the Danish Cardiovascular Screening (DANCAVAS) Trial. N. Engl. J. Med..

[43] Marcus M.E., Manne-Goehler J., Theilmann M., Farzadfar F., Saeedi Moghaddam S., Keykhaei M., Hajebi A., Tschida S., Lemp J.M., Aryal K.K. (2022). Use of statins for the prevention of cardiovascular disease in 41 low-income and middle-income countries: A cross-sectional study of nationally representative, individual-level data. Lancet Glob. Health.

[44] Ference B.A., Kastelein J.J.P., Ray K.K., Ginsberg H.N., Chapman M.J., Packard C.J., Laufs U., Oliver-Williams C., Wood A.M., Butterworth A.S. (2019). Association of Triglyceride-Lowering LPL Variants and LDL-C-Lowering LDLR Variants with Risk of Coronary Heart Disease. JAMA.

[45] Emerging Risk Factors Collaboration (2012). Lipid-related markers and cardiovascular disease prediction. JAMA.

[46] Johannesen C.D.L., Mortensen M.B., Langsted A., Nordestgaard B.G. (2021). Apolipoprotein B and Non-HDL Cholesterol Better Reflect Residual Risk Than LDL Cholesterol in Statin-Treated Patients. J. Am. Coll. Cardiol..

[47] Wald D.S., Bestwick J.P., Morris J.K., Whyte K., Jenkins L., Wald N.J. (2016). Child-Parent Familial Hypercholesterolemia Screening in Primary Care. N. Engl. J. Med..

[48] Wiegman A., Hutten B.A., de Groot E., Rodenburg J., Bakker H.D., Büller H.R., Sijbrands E.J.G., Kastelein J.J.P. (2004). Efficacy and Safety of Statin Therapy in Children with Familial Hypercholesterolemia: A Randomized Controlled Trial. JAMA.

[49] Luirink I.K., Wiegman A., Kusters D.M., Hof M.H., Groothoff J.W., de Groot E., Kastelein J.J.P., Hutten B.A. (2019). 20-Year Follow-up of Statins in Children with Familial Hypercholesterolemia. N. Engl. J. Med..

[50] Blood Pressure Lowering Treatment Trialists’ Collaboration (2021). Pharmacological blood pressure lowering for primary and secondary prevention of cardiovascular disease across different levels of blood pressure: An individual participant-level data meta-analysis. Lancet.

[51] Wright J.T., Williamson J.D., Whelton P.K., Snyder J.K., Sink K.M., Rocco M.V., Reboussin D.M., Rahman M., Oparil S., SPRINT Research Group (2015). A Randomized Trial of Intensive versus Standard Blood-Pressure Control. N. Engl. J. Med..

[52] McEvoy J.W., McCarthy C.P., Bruno R.M., Brouwers S., Canavan M.D., Ceconi C., Christodorescu R.M., Daskalopoulou S.S., Ferro C.J., Gerdts E. (2024). 2024 ESC Guidelines for the management of elevated blood pressure and hypertension. Eur. Heart J..

[53] Jones D.W., Ferdinand K.C., Taler S.J., Johnson H.M., Shimbo D., Abdalla M., Altieri M.M., Bansal N., Bello N.A., Bress A.P. (2025). 2025 AHA/ACC/AANP/AAPA/ABC/ACCP/ACPM/AGS/AMA/ASPC/NMA/PCNA/SGIM Guideline for the Prevention, Detection, Evaluation and Management of High Blood Pressure in Adults. Hypertension.

[54] NCD Risk Factor Collaboration (NCD-RisC) (2021). Worldwide trends in hypertension prevalence and progress in treatment and control from 1990 to 2019: A pooled analysis of 1201 population-representative studies with 104 million participants. Lancet.

[55] Emerging Risk Factors Collaboration (2010). Diabetes mellitus, fasting blood glucose concentration, and risk of vascular disease: A collaborative meta-analysis of 102 prospective studies. Lancet.

[56] Holman R.R., Paul S.K., Bethel M.A., Matthews D.R., Neil H.A.W. (2008). 10-Year Follow-up of Intensive Glucose Control in Type 2 Diabetes. N. Engl. J. Med..

[57] Simmons R.K., Echouffo-Tcheugui J.B., Sharp S.J., Sargeant L.A., Williams K.M., Prevost A.T., Kinmonth A.L., Wareham N.J., Griffin S.J. (2012). Screening for type 2 diabetes and population mortality over 10 years (ADDITION-Cambridge): A cluster-randomised controlled trial. Lancet.

[58] Huang Y., Cai X., Mai W., Li M., Hu Y. (2016). Association between prediabetes and risk of cardiovascular disease and all cause mortality: Systematic review and meta-analysis. BMJ.

[59] Emerging Risk Factors Collaboration (2014). Glycated hemoglobin measurement and prediction of cardiovascular disease. JAMA.

[60] Knowler W.C., Barrett-Connor E., Fowler S.E., Hamman R.F., Lachin J.M., Walker E.A., Nathan D.M. (2002). Diabetes Prevention Program Research Group. Reduction in the incidence of type 2 diabetes with lifestyle intervention or metformin. N. Engl. J. Med..

[61] Gong Q., Zhang P., Wang J., Ma J., An Y., Chen Y., Zhang B., Feng X., Li H., Chen X. (2019). Morbidity and mortality after lifestyle intervention for people with impaired glucose tolerance: 30-year results of the Da Qing Diabetes Prevention Outcome Study. Lancet Diabetes Endocrinol..

[62] Goldberg R.B., Orchard T.J., Crandall J.P., Boyko E.J., Budoff M., Dabelea D., Gadde K.M., Knowler W.C., Lee C.G., Nathan D.M. (2022). Effects of Long-term Metformin and Lifestyle Interventions on Cardiovascular Events in the Diabetes Prevention Program and Its Outcome Study. Circulation.

[63] Rooney M.R., Rawlings A.M., Pankow J.S., Echouffo Tcheugui J.B., Coresh J., Sharrett A.R., Selvin E. (2021). Risk of Progression to Diabetes Among Older Adults with Prediabetes. JAMA Intern. Med..

[64] Matsushita K., Coresh J., Sang Y., Chalmers J., Fox C., Guallar E., Jafar T., Jassal S.K., Landman G.W.D., Muntner P. (2015). Estimated glomerular filtration rate and albuminuria for prediction of cardiovascular outcomes: A collaborative meta-analysis of individual participant data. Lancet Diabetes Endocrinol..

[65] Inker L.A., Eneanya N.D., Coresh J., Tighiouart H., Wang D., Sang Y., Crews D.C., Doria A., Estrella M.M., Froissart M. (2021). New Creatinine- and Cystatin C-Based Equations to Estimate GFR without Race. N. Engl. J. Med..

[66] Willeit P., Kaptoge S., Welsh P., Butterworth A.S., Chowdhury R., Spackman S.A., Pennells L., Gao P., Burgess S., Natriuretic Peptides Studies Collaboration (2016). Natriuretic peptides and integrated risk assessment for cardiovascular disease: An individual-participant-data meta-analysis. Lancet Diabetes Endocrinol..

[67] Pop-Busui R., Januzzi J.L., Bruemmer D., Butalia S., Green J.B., Horton W.B., Knight C., Levi M., Rasouli N., Richardson C.R. (2022). Heart Failure: An Underappreciated Complication of Diabetes. A Consensus Report of the American Diabetes Association. Diabetes Care.

[68] Hagström H., Talbäck M., Andreasson A., Walldius G., Hammar N. (2020). Ability of Noninvasive Scoring Systems to Identify Individuals in the Population at Risk for Severe Liver Disease. Gastroenterology.

[69] Srivastava A., Gailer R., Tanwar S., Trembling P., Parkes J., Rodger A., Suri D., Thorburn D., Sennett K., Morgan S. (2019). Prospective evaluation of a primary care referral pathway for patients with non-alcoholic fatty liver disease. J. Hepatol..

[70] Patel A.P., Wang M., Pirruccello J.P., Ellinor P.T., Ng K., Kathiresan S., Khera A.V. (2021). Lp(a) (Lipoprotein[a]) Concentrations and Incident Atherosclerotic Cardiovascular Disease: New Insights From a Large National Biobank. Arterioscler. Thromb. Vasc. Biol..

[71] Ridker P.M., Moorthy M.V., Cook N.R., Rifai N., Lee I.-M., Buring J.E. (2024). Inflammation, Cholesterol, Lipoprotein(a), and 30-Year Cardiovascular Outcomes in Women. N. Engl. J. Med..

[72] Burgess S., Ference B.A., Staley J.R., Freitag D.F., Mason A.M., Nielsen S.F., Willeit P., Young R., Surendran P., Karthikeyan S. (2018). Association of LPA Variants with Risk of Coronary Disease and the Implications for Lipoprotein(a)-Lowering Therapies: A Mendelian Randomization Analysis. JAMA Cardiol..

[73] Emerging Risk Factors Collaboration (2012). C-reactive protein, fibrinogen, and cardiovascular disease prediction. N. Engl. J. Med..

[74] Emerging Risk Factors Collaboration (2009). Major lipids, apolipoproteins, and risk of vascular disease. JAMA.

[75] Bhatt D.L., Steg P.G., Miller M., Brinton E.A., Jacobson T.A., Ketchum S.B., Doyle R.T., Juliano R.A., Jiao L., Granowitz C. (2019). Cardiovascular Risk Reduction with Icosapent Ethyl for Hypertriglyceridemia. N. Engl. J. Med..

[76] Nicholls S.J., Lincoff A.M., Garcia M., Bash D., Ballantyne C.M., Barter P.J., Davidson M.H., Kastelein J.J.P., Koenig W., McGuire D.K. (2020). Effect of High-Dose Omega-3 Fatty Acids vs Corn Oil on Major Adverse Cardiovascular Events in Patients at High Cardiovascular Risk: The STRENGTH Randomized Clinical Trial. JAMA.

[77] Das Pradhan A., Glynn R.J., Fruchart J.-C., MacFadyen J.G., Zaharris E.S., Everett B.M., Campbell S.E., Oshima R., Amarenco P., Blom D.J. (2022). Triglyceride Lowering with Pemafibrate to Reduce Cardiovascular Risk. N. Engl. J. Med..

[78] Curry S.J., Krist A.H., Owens D.K., Barry M.J., Caughey A.B., Davidson K.W., Doubeni C.A., Epling J.W., Kemper A.R., US Preventive Services Task Force (2018). Risk Assessment for Cardiovascular Disease with Nontraditional Risk Factors: US Preventive Services Task Force Recommendation Statement. JAMA.

[79] Messenger B., Li D., Nasir K., Carr J.J., Blankstein R., Budoff M.J. (2016). Coronary calcium scans and radiation exposure in the multi-ethnic study of atherosclerosis. Int. J. Cardiovasc. Imaging.

[80] Ganguli I., Simpkin A.L., Lupo C., Weissman A., Mainor A.J., Orav E.J., Rosenthal M.B., Colla C.H., Sequist T.D. (2019). Cascades of Care After Incidental Findings in a US National Survey of Physicians. JAMA Netw. Open.

[81] Inaba Y., Chen J.A., Bergmann S.R. (2012). Carotid plaque, compared with carotid intima-media thickness, more accurately predicts coronary artery disease events: A meta-analysis. Atherosclerosis.

[82] Gepner A.D., Young R., Delaney J.A., Tattersall M.C., Blaha M.J., Post W.S., Gottesman R.F., Kronmal R., Budoff M.J., Burke G.L. (2015). Comparison of coronary artery calcium presence, carotid plaque presence, and carotid intima-media thickness for cardiovascular disease prediction in the Multi-Ethnic Study of Atherosclerosis. Circ. Cardiovasc. Imaging.

[83] Nambi V., Chambless L., Folsom A.R., He M., Hu Y., Mosley T., Volcik K., Boerwinkle E., Ballantyne C.M. (2010). Carotid intima-media thickness and presence or absence of plaque improves prediction of coronary heart disease risk: The ARIC (Atherosclerosis Risk In Communities) study. J. Am. Coll. Cardiol..

[84] Baber U., Mehran R., Sartori S., Schoos M.M., Sillesen H., Muntendam P., Garcia M.J., Gregson J., Pocock S., Falk E. (2015). Prevalence, impact, and predictive value of detecting subclinical coronary and carotid atherosclerosis in asymptomatic adults: The BioImage study. J. Am. Coll. Cardiol..

[85] Näslund U., Ng N., Lundgren A., Fhärm E., Grönlund C., Johansson H., Lindahl B., Lindahl B., Lindvall K., Nilsson S.K. (2019). Visualization of asymptomatic atherosclerotic disease for optimum cardiovascular prevention (VIPVIZA): A pragmatic, open-label, randomised controlled trial. Lancet.

[86] Bengtsson A., Norberg M., Ng N., Carlberg B., Grönlund C., Hultdin J., Lindahl B., Lindahl B., Nordin S., Nyman E. (2021). The beneficial effect over 3 years by pictorial information to patients and their physician about subclinical atherosclerosis and cardiovascular risk: Results from the VIPVIZA randomized clinical trial. Am. J. Prev. Cardiol..

[87] Fowkes F.G., Murray G.D., Butcher I., Heald C.L., Lee R.J., Chambless L.E., Folsom A.R., Hirsch A.T., Dramaix M., Ankle Brachial Index Collaboration (2008). Ankle brachial index combined with Framingham Risk Score to predict cardiovascular events and mortality: A meta-analysis. JAMA.

[88] Rai A., Baridkazemi S., Sobhiyeh M., Amiri M., Ghorbani M., Shafiei S., Rahmanipour E. (2024). Prevalence and risk factors associated with coronary artery disease in Iranian patients with peripheral artery disease. J. Vasc. Nurs..

[89] Aboyans V., Criqui M.H., Abraham P., Allison M.A., Creager M.A., Diehm C., Fowkes F.G.R., Hiatt W.R., Jönsson B., Lacroix P. (2012). Measurement and Interpretation of the Ankle-Brachial Index: A Scientific Statement from the American Heart Association. Circulation.

[90] Fowkes F.G., Price J.F., Stewart M.C., Butcher I., Leng G.C., Pell A.C., Sandercock P.A., Fox K.A., Lowe G.D., Murray G.D. (2010). Aspirin for prevention of cardiovascular events in a general population screened for a low ankle brachial index: A randomized controlled trial. JAMA.

[91] Lindholt J.S., Søgaard R. (2017). Population screening and intervention for vascular disease in Danish men (VIVA): A randomised controlled trial. Lancet.

[92] Gornik H.L., Aronow H.D., Goodney P.P., Arya S., Brewster L.P., Byrd L., Chandra V., Drachman D.E., Eaves J.M., Ehrman J.K. (2024). ACC/AHA/AACVPR/APMA/ABC/SCAI/SVM/SVN/SVS/SIR/VESS Guideline for the Management of Lower Extremity Peripheral Artery Disease: A Report of the American College of Cardiology/American Heart Association Joint Committee on Clinical Practice Guidelines. Circulation.

[93] Khera A.V., Chaffin M., Aragam K.G., Haas M.E., Roselli C., Choi S.H., Natarajan P., Lander E.S., Lubitz S.A., Ellinor P.T. (2018). Genome-wide polygenic scores for common diseases identify individuals with risk equivalent to monogenic mutations. Nat. Genet..

[94] Elliott J., Bodinier B., Bond T.A., Chadeau-Hyam M., Evangelou E., Moons K.G.M., Dehghan A., Muller D.C., Elliott P., Tzoulaki I. (2020). Predictive Accuracy of a Polygenic Risk Score-Enhanced Prediction Model vs a Clinical Risk Score for Coronary Artery Disease. JAMA.

[95] Mosley J.D., Gupta D.K., Tan J., Yao J., Wells Q.S., Shaffer C.M., Kundu S., Robinson-Cohen C., Psaty B.M., Rich S.S. (2020). Predictive Accuracy of a Polygenic Risk Score Compared with a Clinical Risk Score for Incident Coronary Heart Disease. JAMA.

[96] Mega J.L., Stitziel N.O., Smith J.G., Chasman D.I., Caulfield M.J., Devlin J.J., Nordio F., Hyde C.L., Cannon C.P., Sacks F.M. (2015). Genetic risk, coronary heart disease events, and the clinical benefit of statin therapy: An analysis of primary and secondary prevention trials. Lancet.

[97] Kullo I.J., Jouni H., Austin E.E., Brown S.-A., Kruisselbrink T.M., Isseh I.N., Haddad R.A., Marroush T.S., Shameer K., Olson J.E. (2016). Incorporating a Genetic Risk Score Into Coronary Heart Disease Risk Estimates: Effect on Low-Density Lipoprotein Cholesterol Levels (the MI-GENES Clinical Trial). Circulation.

[98] Naderian M., Hamed M.E., Vaseem A.A., Norland K., Dikilitas O., Teymourzadeh A., Bailey K.R., Kullo I.J. (2025). Effect of Disclosing a Polygenic Risk Score for Coronary Heart Disease on Adverse Cardiovascular Events. Circ. Genom. Precis. Med..

[99] Hollands G.J., French D.P., Griffin S.J., Prevost A.T., Sutton S., King S., Marteau T.M. (2016). The impact of communicating genetic risks of disease on risk-reducing health behaviour: Systematic review with meta-analysis. BMJ.

[100] Martin A.R., Kanai M., Kamatani Y., Okada Y., Neale B.M., Daly M.J. (2019). Clinical use of current polygenic risk scores may exacerbate health disparities. Nat. Genet..

[101] Schunkert H., Di Angelantonio E., Inouye M., Patel R.S., Ripatti S., Widen E., Sanderson S.C., Kaski J.P., McEvoy J.W., Vardas P. (2025). Clinical utility and implementation of polygenic risk scores for predicting cardiovascular disease: A clinical consensus statement of the ESC Council on Cardiovascular Genomics, the ESC Cardiovascular Risk Collaboration, and the European Association of Preventive Cardiology. Eur. Heart J..

[102] Jaiswal S., Natarajan P., Silver A.J., Gibson C.J., Bick A.G., Shvartz E., McConkey M., Gupta N., Gabriel S., Ardissino D. (2017). Clonal Hematopoiesis and Risk of Atherosclerotic Cardiovascular Disease. N. Engl. J. Med..

[103] Helgason H., Eiriksdottir T., Ulfarsson M.O., Choudhary A., Lund S.H., Ivarsdottir E.V., Hjorleifsson Eldjarn G., Einarsson G., Ferkingstad E., Moore K.H.S. (2023). Evaluation of Large-Scale Proteomics for Prediction of Cardiovascular Events. JAMA.

[104] Bellamy L., Casas J.-P., Hingorani A.D., Williams D.J. (2007). Pre-eclampsia and risk of cardiovascular disease and cancer in later life: Systematic review and meta-analysis. BMJ.

[105] Honigberg M.C., Zekavat S.M., Aragam K., Finneran P., Klarin D., Bhatt D.L., Januzzi J.L., Scott N.S., Natarajan P. (2019). Association of Premature Natural and Surgical Menopause with Incident Cardiovascular Disease. JAMA.

[106] Markovitz A.R., Stuart J.J., Horn J., Williams P.L., Rimm E.B., Missmer S.A., Tanz L.J., Haug E.B., Fraser A., Timpka S. (2019). Does pregnancy complication history improve cardiovascular disease risk prediction? Findings from the HUNT study in Norway. Eur. Heart J..

[107] Moynihan R., Doust J., Henry D. (2012). Preventing overdiagnosis: How to stop harming the healthy. BMJ.

[108] Chen W., Thomas J., Sadatsafavi M., FitzGerald J.M. (2015). Risk of cardiovascular comorbidity in patients with chronic obstructive pulmonary disease: A systematic review and meta-analysis. Lancet Respir. Med..

[109] Mangione C.M., Barry M.J., Nicholson W.K., Cabana M., Chelmow D., Rucker Coker T., Davidson K.W., Davis E.M., Donahue K.E., US Preventive Services Task Force (2022). Screening for Obstructive Sleep Apnea in Adults: US Preventive Services Task Force Recommendation Statement. JAMA.

[110] McEvoy R.D., Antic N.A., Heeley E., Luo Y., Ou Q., Zhang X., Mediano O., Chen R., Drager L.F., Liu Z. (2016). CPAP for Prevention of Cardiovascular Events in Obstructive Sleep Apnea. N. Engl. J. Med..

[111] Yu J., Zhou Z., McEvoy R.D., Anderson C.S., Rodgers A., Perkovic V., Neal B. (2017). Association of Positive Airway Pressure with Cardiovascular Events and Death in Adults with Sleep Apnea: A Systematic Review and Meta-analysis. JAMA.

