# Supplementary material for: From Risk Markers to Preventive Action: A Proposed Semi-Quantitative Clinical Actionability Framework for Cardiometabolic Precision Prevention in Primary Care—A Narrative Review
Source: J Clin Med. 2026 Nov 7;15(21):8706. doi: 10.3390/jcm15218706 (PMC13764083; doi:10.3390/jcm15218706)
Supplement: Supplementary file 1 [file jcm-15-08706-s001.zip › jcm-4629366-supplementary.pdf]

Supplementary Materials

From Risk Markers to Preventive Action: A Proposed Semi-Quantitative Clinical Actionability Framework for Cardiometabolic Precision Prevention in Primary Care—A Narrative Review

Table S1. Domain-level scoring rationale, D3 routes and D7 subcomponent ratings for the 19 marker–context–action use cases.

Domain	Score	Rationale and key evidence
**1. LDL-C / non-HDL-C** — All adults (40–75 years; lifetime perspective); initiate or intensify lipid-lowering therapy. Total 20; Tier A.		
D1 Measurement readiness	3	Inexpensive, standardised and repeatable; non-HDL cholesterol is derived from the same panel.
D2 Prognostic validity	3	Causal, dose-dependent and log-linear relation with ASCVD from genetic, epidemiological and randomised evidence [22].
D3 Decision-relevant information	3	Route C: cholesterol fractions from the same lipid panel enter the major risk equations [10,13].
D4 Therapeutic linkage	3	Direct pathway (target route): LDL-C lowering reduces major vascular events, including at low baseline risk [23].
D5 Clinical-utility evidence	3	Randomised evidence of hard-outcome benefit from treatment directed at the marker [23]; the same trials inform D4 (efficacy of LDL-C lowering) and D5 (hard-outcome benefit of treatment directed at the marker), as specified for target-route markers in Table S5.
D6 Harms and burden	3	Negligible harms of the testing pathway; minimal labelling. Adverse effects of statin therapy are reflected in the outcome evidence (D4–D5).
D7 Transportability (T) and equity (E)	2	T 2: standardised measurement, and relative treatment benefit maintained across categories of baseline risk and largely irrespective of age and sex [23], but consistent performance across ancestry and socioeconomic groups is not documented in the evidence cited. E 2: access to and uptake of lipid-lowering therapy are uneven, with low statin use in many health systems [43]; equitable implementation not affirmatively demonstrated.
**2. Apolipoprotein B** — Hypertriglyceridaemia, diabetes, obesity or low LDL-C (discordance); refine atherogenic burden and treatment intensity. Total 16; Tier B.		
D1 Measurement readiness	2	Standardised immunoassay, widely available but not reported routinely in all health systems.
D2 Prognostic validity	3	Particle number is the causal exposure: LPL and LDLR variants have near-identical associations with coronary heart disease per unit of apoB [44]; in the 22 studies (91,307 participants) of an individual-participant meta-analysis that measured apolipoproteins, apoB had an association with coronary heart disease of very similar shape and magnitude to that of non-HDL cholesterol, and the apoB/apolipoprotein A-I ratio had an HR of 1.49 per SD [74].

Domain	Score	Rationale and key evidence
D3 Decision-relevant information	2	Route I: informative when apoB and LDL-C are discordant (hypertriglyceridaemia, diabetes, obesity or low LDL-C). In the general population, adding apoB and apolipoprotein A-I barely improved discrimination (C-index change 0.0006) [45], whereas in statin-treated individuals discordantly high apoB with LDL-C below the median was associated with myocardial infarction (HR 1.49) [46], indicating that LDL-C underestimates risk in this subgroup. Because [46] is a discordance analysis rather than a formal reclassification analysis, the score of 2 is a judgement; a score of 1 would leave the use case in Tier B (total 15). Guideline support for apoB testing to assess residual risk [9] is not counted as decision-relevant information.
D4 Therapeutic linkage	3	Direct pathway: apoB-containing lipoproteins are the causal exposure [44], and lowering them with statin therapy reduces major vascular events in randomised trials [22,23]; apoB is used to assess residual risk once LDL-C and non-HDL-C goals are met [9].
D5 Clinical-utility evidence	1	Statin trials lowered apoB, but apoB was not used to select participants or guide treatment; no randomised trial has compared apoB-guided with LDL-C-guided treatment, so evidence for the decision informed by apoB in discordance contexts is genetic [44] and observational.
D6 Harms and burden	3	Negligible harms of the testing pathway.
D7 Transportability (T) and equity (E)	2	T 2 and E 2: no major limitation identified, but consistent performance across groups and equitable access have not been affirmatively shown.
**3. Familial hypercholesterolaemia case-finding** — LDL-C ≥ 190 mg/dL (4.9 mmol/L), clinical criteria, child–parent or cascade screening; early high-intensity LDL-C lowering; cascade testing. Total 17; Tier A.		
D1 Measurement readiness	2	LDL-C universally available; access to genetic testing varies.
D2 Prognostic validity	3	Markedly premature ASCVD without early treatment: cumulative incidence of cardiovascular events of 26% by the age of 39 years among affected parents [49]; mutations that reduce LDL-receptor function cause markedly higher LDL-C and a dose-dependent increase in ASCVD risk [22].
D3 Decision-relevant information	3	Route S: defines a high-risk state on its own, independently of risk equations [29].
D4 Therapeutic linkage	3	Direct pathway: early high-intensity LDL-C lowering, whose efficacy on hard outcomes is established [23], and child–parent screening that identifies affected relatives [47].
D5 Clinical-utility evidence	2	In a placebo-controlled trial in children with FH, pravastatin lowered LDL-C, a validated intermediate outcome, and reduced progression of carotid intima–media thickness [48]; during 20 years of follow-up of this cohort, LDL-C fell from 237.3 to 160.7 mg/dL and event rates were low [49]; no placebo-controlled hard-outcome trial in FH.
D6 Harms and burden	2	Implications of a genetic diagnosis for the person tested, including labelling; the burden of cascade testing falls on relatives and is not scored in D6 (Table S5).
D7 Transportability (T) and equity (E)	2	T 2: detection yield and variant spectrum differ between populations. E 2: uptake of cascade testing and of early treatment varies; equitable implementation not affirmatively shown.

Domain	Score	Rationale and key evidence
**4. Blood pressure (office, with out-of-office confirmation)** — All adults; diagnose hypertension; initiate or intensify treatment. Total 19; Tier A.		
D1 Measurement readiness	3	Universally available; ambulatory measurement carries most of the prognostic information [34], and out-of-office measurement is recommended to confirm the diagnosis [52,53].
D2 Prognostic validity	3	Strong, graded association with mortality; ambulatory BP is the most informative measure [34]; systolic BP is a core predictor in risk equations derived from 45 cohorts (SCORE2) and 25 data sets (PREVENT) [10,13].
D3 Decision-relevant information	3	Route C: constituent of the major risk equations [10,13].
D4 Therapeutic linkage	3	Direct pathway (target route): each 5 mmHg reduction in systolic BP lowers the risk of major cardiovascular events [50].
D5 Clinical-utility evidence	3	Randomised evidence of hard-outcome benefit across baseline BP levels and with intensive targets [50,51]; as for LDL-C, the same trials inform D4 and D5 for different properties (Table S5).
D6 Harms and burden	2	Overdiagnosis from unconfirmed office readings (white-coat hypertension carried no excess mortality [34]) and the burden of out-of-office confirmation. Adverse effects of intensive treatment [51] are reflected in the outcome evidence (D4–D5).
D7 Transportability (T) and equity (E)	2	T 2: treatment benefit consistent across baseline BP categories and in people with and without prior cardiovascular disease [50], but consistent performance across sex, ancestry and socioeconomic groups is not documented in the evidence cited. E 2: treatment and control rates vary widely between countries and between women and men [54].
**5. HbA1c or fasting glucose: type 2 diabetes** — Adults with overweight, obesity or other risk factors; diagnose T2D; glucose-lowering, lipid-lowering and organ-protective therapy. Total 18; Tier A.		
D1 Measurement readiness	3	Routine, standardised measurement with established diagnostic thresholds.
D2 Prognostic validity	3	Diabetes confers about a two-fold risk of coronary heart disease and ischaemic stroke in a meta-analysis of 102 prospective studies [55].
D3 Decision-relevant information	3	Route S (also route C): T2D is a clinically recognised condition whose cardiovascular risk European prevention guidance classifies as moderate to very high according to diabetes duration and target-organ damage [29], with about twice the risk of coronary heart disease and ischaemic stroke [55], and enters risk equations such as PREVENT [10].
D4 Therapeutic linkage	3	Direct pathway: in newly diagnosed T2D, glucose-lowering therapy was followed by fewer myocardial infarctions and deaths over 10 years of post-trial monitoring [56]; SGLT2 inhibition and finerenone reduce kidney and cardiovascular events in T2D with CKD [35,36].
D5 Clinical-utility evidence	2	Treatment of the condition detected reduces hard outcomes [56], but screening high-risk adults did not reduce all-cause, cardiovascular or diabetes-related mortality over a median of 9.6 years [57]; under the D5 reading rules, a null trial of the testing strategy limits the score to 2.
D6 Harms and burden	2	Diagnosis requires confirmatory testing; false-positive results and labelling.
D7 Transportability (T) and equity (E)	2	T 2: HbA1c is affected by anaemia, haemoglobin variants and ancestry-related differences. E 2: many adults with diabetes remain untreated [6].

Domain	Score	Rationale and key evidence
**6. HbA1c or fasting glucose: prediabetes** — Adults with overweight, obesity or other risk factors; identify prediabetes; structured lifestyle programme. Total 14; Tier B.		
D1 Measurement readiness	3	Routine, standardised measurement; definitions of prediabetes differ between organisations.
D2 Prognostic validity	2	Independent but modest association with cardiovascular disease (relative risks 1.13–1.30 across definitions) in prospective cohorts [58].
D3 Decision-relevant information	1	Route I: fasting glucose or impaired fasting glucose did not significantly improve the prediction of vascular disease in people without diabetes [55]; adding HbA1c increased the C-index by 0.0018 (95% CI 0.0003–0.0033) without a significant improvement in reclassification [59], a statistically significant but marginal increment; HbA1c is not a core predictor in PREVENT and enters only an optional model [10]. Route S is not credited, because prediabetes is a risk factor rather than a disease [58] and European prevention guidance does not treat it as a high-risk category [29]; crediting route S would give D3 = 3 (total 16, Tier B).
D4 Therapeutic linkage	2	Target with efficacy on an intermediate outcome: lifestyle intervention and metformin prevent or delay T2D [60]; effects on hard outcomes are inconsistent [61,62].
D5 Clinical-utility evidence	2	Randomised evidence of reduced incidence of diabetes, a validated intermediate outcome [60]; hard-outcome evidence conflicting, with benefit in the Da Qing study [61] but not in the Diabetes Prevention Program Outcomes Study [62]; these trials enrolled people with impaired glucose tolerance, and intervention data for prediabetes defined by HbA1c or fasting glucose are few [58].
D6 Harms and burden	2	Labelling and overdiagnosis, particularly in older adults, in whom regression to normoglycaemia and death are more frequent than progression [63].
D7 Transportability (T) and equity (E)	2	T 2: HbA1c is affected by anaemia, haemoglobin variants and ancestry. E 2: access to structured lifestyle programmes is uneven.
**7. eGFR plus UACR** — Adults with diabetes and/or hypertension; confirm and stage CKD; consider guideline-indicated RAS blockade, SGLT2 inhibitor and, in T2D, finerenone; intensify risk management. Total 19; Tier A.		
D1 Measurement readiness	3	Inexpensive and standardised; race-free estimating equations available [65].
D2 Prognostic validity	3	Independent associations with cardiovascular mortality and HF, improving prediction beyond traditional risk factors, in 637,315 individuals from 24 cohorts [64].
D3 Decision-relevant information	3	Routes C and S (also route I): improves prediction of cardiovascular death and HF [64]; eGFR is a core predictor and UACR an optional predictor in PREVENT [10]; moderate or severe CKD is classified as a high-risk or very-high-risk state [29].
D4 Therapeutic linkage	3	Direct pathway: SGLT2 inhibition reduces kidney and cardiovascular events in CKD defined by eGFR and UACR [35], as does finerenone in people with T2D and CKD [36]; RAS blockade is recommended or suggested in albuminuric CKD according to the level of albuminuria and the presence of diabetes [32].
D5 Clinical-utility evidence	3	Randomised evidence of hard-outcome benefit from treatment directed at the condition the markers define [35,36]; these trials enrolled people defined by eGFR and UACR and therefore also inform D5 (Table S5).

Domain	Score	Rationale and key evidence
D6 Harms and burden	2	Biological variability of UACR requires confirmatory testing [32]; labelling as CKD.
D7 Transportability (T) and equity (E)	2	T 2: race-free estimating equations adopted, with smaller but residual differences between groups [65]. E 2: albuminuria testing is infrequent and uneven [12].
**8. Natriuretic peptides (BNP or NT-proBNP)** — Adults with T2D or other cardiovascular risk factors, without HF; identify stage B HF; echocardiography, intensified cardioprotective therapy. Total 15; Tier B.		
D1 Measurement readiness	2	Widely available immunoassays, but BNP and NT-proBNP are distinct analytes with assay-specific thresholds (BNP ≥ 50 pg/mL in [38]; NT-proBNP > 125 pg/mL in [37]) that are not interchangeable; cost exceeds that of routine chemistry.
D2 Prognostic validity	3	Top versus bottom third of NT-proBNP: risk ratio 2.00 for coronary heart disease, stroke or HF in an individual-participant meta-analysis [66].
D3 Decision-relevant information	2	Route I: C-index increase of 0.019 (95% CI 0.016–0.022) for coronary heart disease, stroke or HF beyond conventional risk factors in an individual-participant meta-analysis [66]; identifies people for echocardiography in the decision zone. Route S was not credited, which is a judgement: recent prevention guidance defines pre-HF by cardiac biomarker elevation or imaging evidence of cardiac dysfunction without symptoms of HF, includes it in CKM stage 3 and recommends intensive lifestyle and risk-factor management for it and, in T2D or CKD, SGLT2 inhibition, but it recommends biomarker testing in adults with increased predicted HF risk to guide further diagnostic testing, such as cardiac imaging, and coordinated care [2], which is the triage role scored here; crediting route S would give D3 = 3 (total 16, Tier B).
D4 Therapeutic linkage	2	Indirect pathway: triage to further testing, such as echocardiography, and individualised preventive treatment [67].
D5 Clinical-utility evidence	2	Small randomised trials: fewer cardiac hospitalisations or deaths with intensified therapy in T2D with elevated NT-proBNP [37]; lower prevalence of left ventricular dysfunction with or without HF after BNP screening and collaborative care [38], in which emergency hospitalisation for major cardiovascular events, a secondary end point, was also less frequent (incidence rate ratio 0.60; 95% CI 0.45–0.81). Because the hard-outcome results come from two small trials, one of them for a secondary end point, and have not been replicated, the score of 2 is a judgement; a score of 3 would give a total of 16 (Tier B).
D6 Harms and burden	2	False-positive results increase demand for imaging; in the screening trial, the intervention group underwent more cardiovascular investigations (incidence rate ratio 1.71; 95% CI 1.61–1.83) [38].
D7 Transportability (T) and equity (E)	2	T 2: lower concentrations in obesity; interpretation depends on age, sex and kidney function. E 2: equitable access not affirmatively shown.
**9. FIB-4 index** — Adults with T2D, obesity or ≥ 2 metabolic risk factors; rule out advanced fibrosis; second-line test or referral. Total 14; Tier B.		
D1 Measurement readiness	3	Calculated from routine laboratory tests [3].
D2 Prognostic validity	2	FIB-4 and other non-invasive scores predict incident severe liver disease in a large population cohort, with modest accuracy that improves in people with metabolic risk factors [68]; advanced fibrosis is associated with cardiovascular events [4].

Domain	Score	Rationale and key evidence
D3 Decision-relevant information	2	Route I: supports rule-out of advanced fibrosis within a stepwise pathway [3]; in a primary-care pathway starting with FIB-4, unnecessary referrals to secondary care fell by 81% (OR 0.193; 95% CI 0.111–0.337) [69].
D4 Therapeutic linkage	2	Indirect pathway: triage to elastography, specialist referral and treatment, including resmetirom for non-cirrhotic steatohepatitis with significant fibrosis where approved [3], whose benefit has been shown on histological end points.
D5 Clinical-utility evidence	1	Primary-care pathways increase the detection of advanced fibrosis, a process outcome; no hard-outcome data [69].
D6 Harms and burden	2	Indeterminate and false-positive results lead to second-line testing and referral.
D7 Transportability (T) and equity (E)	2	T 2: performance and thresholds depend on age (age-specific cut-offs), and validation is limited in some populations; in a population cohort, predictive ability was higher in men and decreased with time [68]. E 2: equitable access to second-line testing not affirmatively shown.
**10. Lipoprotein(a)** — Adults (once in adulthood); intensify LDL-C lowering and risk-factor control; cascade testing. Total 13; Tier B.		
D1 Measurement readiness	2	Widely available, but assays are incompletely standardised, some are sensitive to apolipoprotein(a) isoform size, and no standard factor for converting mass into molar units is recommended [33].
D2 Prognostic validity	3	Causal by genetic evidence [25]; linear risk gradient [70]; a single measurement predicted events over 30 years in women [71].
D3 Decision-relevant information	1	Route I: across the population, adding Lp(a) to conventional risk factors increased the C-index by 0.0016, with net reclassification improvement below 1% and an estimated 4.1% of intermediate-risk people reclassified to high risk [45]; reclassification is confined to the minority with high concentrations. Recognition as a risk modifier or risk-enhancing factor [8,9,33] is not itself counted as decision-relevant information. Score changed during revision from 2 to 1.
D4 Therapeutic linkage	2	Indirect pathway (triage): no proven Lp(a)-lowering therapy, and large absolute reductions are probably required [72]; elevated values prompt more intensive LDL-C lowering [9].
D5 Clinical-utility evidence	1	Evidence limited to genetic and modelling analyses of the expected benefit of Lp(a) lowering [72]; no randomised trial of Lp(a)-guided care and no peer-reviewed outcome trial demonstrating cardiovascular event reduction with Lp(a)-lowering therapy.
D6 Harms and burden	2	Labelling without a specific therapy; implications for relatives, which arise through cascade testing, are not scored in D6 (Table S5).
D7 Transportability (T) and equity (E)	2	T 2: concentrations differ between ancestry groups, which complicates common thresholds. E 2: equitable access not affirmatively shown. Assay and unit heterogeneity is scored in D1 only [33].
**11. hs-CRP** — Adults at borderline or intermediate risk; risk enhancer informing statin initiation. Total 14; Tier B.		
D1 Measurement readiness	3	Inexpensive, standardised high-sensitivity assays.
D2 Prognostic validity	2	Independent but modest association [73]; not causal by Mendelian randomisation [24].

Domain	Score	Rationale and key evidence
D3 Decision-relevant information	1	Route I: C-index increase of 0.0039 beyond conventional risk factors [73]; in intermediate-risk adults, the decision zone, hs-CRP was independently associated with incident coronary heart disease (HR 1.28; 95% CI 1.00–1.64), with a net reclassification improvement for coronary heart disease of 0.079, compared with 0.659 for CAC [39]. The score of 1 is a judgement; a score of 2 would give a total of 15 (Tier B).
D4 Therapeutic linkage	2	Indirect pathway: triage to statin therapy [18].
D5 Clinical-utility evidence	2	Hard-outcome benefit of statin therapy in a population selected by hs-CRP, without a marker-guided comparator [18].
D6 Harms and burden	2	Acute-phase elevations require repeat testing; only persistently elevated values are considered [8].
D7 Transportability (T) and equity (E)	2	T 2: concentrations vary with sex and adiposity. E 2: equitable access not affirmatively shown. Variation with intercurrent illness is scored in D6 (repeat testing).
**12. Triglycerides** — Adults with triglycerides 150–499 mg/dL (1.7–5.6 mmol/L); initiate triglyceride-directed drug therapy. Total 10; Tier C.		
D1 Measurement readiness	3	Routine measurement.
D2 Prognostic validity	1	Association with coronary heart disease not independent of HDL and non-HDL cholesterol (HR 0.99; 95% CI 0.94–1.05) [74]; risk tracks apoB-containing particles [44].
D3 Decision-relevant information	0	Route I: no statistically significant increment in prediction has been shown: adding triglycerides to conventional risk factors that included total and HDL cholesterol changed the C-index by 0.0000 (95% CI −0.0001 to 0.0002) in 24 cohorts [45]; the absence of an independent association with coronary heart disease [74] is scored in D2, and the risk carried by triglyceride-rich lipoproteins tracks apoB-containing particles [44]. Score changed during revision from 1 to 0, because a score of 1 requires a statistically significant increment.
D4 Therapeutic linkage	1	Efficacy of triglyceride-directed therapy not established in the marker-defined group: benefit with icosapent ethyl [75] but not with other agents [76,77], and no significant benefit in the primary-prevention cohort of REDUCE-IT [75].
D5 Clinical-utility evidence	1	Conflicting randomised evidence on hard outcomes [75–77].
D6 Harms and burden	2	High within-person and postprandial variability often requires repeat measurement before a drug decision; labelling. Adverse effects of therapy, such as atrial fibrillation with icosapent ethyl [75], are reflected in D4–D5.
D7 Transportability (T) and equity (E)	2	T 2 and E 2: no major limitation identified, but consistent performance and equitable implementation have not been affirmatively shown.
**13. Coronary artery calcium score** — Adults aged 40–75 years at borderline or intermediate risk with an uncertain statin decision; initiate, intensify or defer statin therapy. Total 15; Tier B.		
D1 Measurement readiness	2	Requires computed tomography referral; availability and test cost vary between health systems.
D2 Prognostic validity	3	Strong, graded prediction across four ethnic groups [26].
D3 Decision-relevant information	3	Route I: greatest improvement in discrimination and reclassification among the novel markers compared in intermediate-risk adults [39]; a score of zero de-risks [40].
D4 Therapeutic linkage	2	Indirect pathway: triage to statin initiation, intensification or deferral [8,9].

Domain	Score	Rationale and key evidence
D5 Clinical-utility evidence	2	Improved risk-factor profiles without increased downstream testing in a randomised trial [41]; screening that included CAC did not significantly reduce all-cause mortality [42].
D6 Harms and burden	1	Radiation, with an effective dose of about 1 mSv [79]; incidental findings and cascades of care [80]; downstream testing was not increased in one randomised trial [41].
D7 Transportability (T) and equity (E)	2	T 3: graded prediction in four ethnic groups [26]. E 2: equitable access to scanning has not been shown, and uptake that depends on reimbursement may differ between socioeconomic groups.
**14. Carotid plaque ultrasound** — Adults at moderate risk; reclassify risk; communicate subclinical disease. Total 13; Tier B.		
D1 Measurement readiness	2	Widely available but operator-dependent.
D2 Prognostic validity	2	Plaque predicts myocardial infarction more accurately than intima–media thickness in population-based studies, with modest discrimination [81].
D3 Decision-relevant information	1	Route I: in MESA, adding plaque presence to traditional risk factors increased the C-statistic for cardiovascular disease from 0.756 to 0.760 ($P < 0.05$), less than CAC presence did (0.776) [82]; intima–media thickness alone adds little [39]. Larger improvements were reported for plaque combined with intima–media thickness (net reclassification improvement 9.9%) [83] and for three-dimensional plaque burden in older adults followed for a median of 2.7 years (23%) [84]; because these measurements differ from the scored use case, the most direct evidence was credited (marginal increment), and crediting them would give D3 = 2 (total 14, Tier B). Stand-alone discrimination [81] is scored in D2.
D4 Therapeutic linkage	2	Indirect pathway: triage to statin therapy and risk-factor intensification.
D5 Clinical-utility evidence	2	In a randomised trial of pictorial information on carotid ultrasound findings, LDL-C, a validated intermediate outcome, was lower in the intervention group at 1 and 3 years (differences 0.17 and 0.23 mmol/L) [86]; the primary outcomes, calculated risk scores, also improved [85] but are not counted as intermediate outcomes; no hard-outcome evidence.
D6 Harms and burden	2	Incidental findings and labelling of subclinical disease; operator dependence is scored in D1.
D7 Transportability (T) and equity (E)	2	T 2: limited data in diverse populations. E 2: equitable access not affirmatively shown.
**15. Ankle–brachial index** — Asymptomatic adults (screening); identify asymptomatic PAD; initiate or intensify statin therapy. Total 13; Tier B.		
D1 Measurement readiness	2	Simple but time-consuming Doppler measurement.

Domain	Score	Rationale and key evidence
D2 Prognostic validity	3	Individual-participant meta-analysis of 16 population cohorts from Australia, Europe and the United States (48,294 participants without coronary heart disease), predominantly white but including Japanese American, American Indian, Black and Hispanic participants: risk rose consistently as the ABI fell below 1.11 (reverse J-shaped relation), and an ABI of 0.90 or less, compared with 1.11–1.40, was associated with higher 10-year cardiovascular mortality in men (HR 4.2) and women (HR 3.5), independently of the Framingham risk score [87]. The unreliability of the ABI in poorly compressible arteries [89] is a measurement limitation in particular groups and is scored in D7, not here. Because the cohorts were predominantly white, the anchor element of diverse populations is met only in part, so this score is a judgement; a score of 2 would give a total of 12 (Tier C). Score changed during revision from 2 to 3.
D3 Decision-relevant information	1	Route I: in intermediate-risk adults, the decision zone, the increment was small (net reclassification improvement for coronary heart disease 0.036, compared with 0.659 for CAC) [39]; the reclassification of 19% of men and 36% of women across risk categories in general populations [87] counts category changes without a net reclassification analysis and was not credited. Route S was not credited, which is a judgement: an ABI of 0.90 or less is the diagnostic threshold for PAD [89], but recent prevention guidance describes the ABI as a measure of atherosclerosis with utility in risk assessment and limited evidence of utility for guiding clinical care [2]; crediting route S would give D3 = 3 (total 15, Tier B).
D4 Therapeutic linkage	2	Indirect pathway: identifies asymptomatic peripheral artery disease, which carries high cardiovascular risk [87] and hence a larger expected absolute benefit from statin therapy [23]; single antiplatelet therapy is considered after clinical assessment, taking bleeding risk into account: it is reasonable in asymptomatic PAD on the basis of expert opinion [92], although aspirin did not reduce vascular events after ABI screening [90].
D5 Clinical-utility evidence	1	Conflicting randomised evidence: aspirin did not reduce vascular events after ABI screening [90]; combined screening that included the ABI reduced all-cause mortality in one trial [91] but not significantly in another [42]; no trial of ABI-guided statin allocation; evidence judged insufficient [78].
D6 Harms and burden	2	False-positive results and follow-up testing.
D7 Transportability (T) and equity (E)	2	T 2: unreliable when medial arterial calcification makes arteries poorly compressible [89], which is more common in diabetes and advanced CKD; the toe–brachial index can be used instead [89]. E 2: equitable access not affirmatively shown.
**16. Pregnancy complications and premature menopause** — Women (history-taking); earlier, more frequent risk-factor surveillance. Total 13; Tier C.		
D1 Measurement readiness	3	History-taking.
D2 Prognostic validity	2	Pre-eclampsia is associated with later ischaemic heart disease and hypertension [104]; premature natural and surgical menopause are associated with incident cardiovascular disease after adjustment for conventional risk factors [105].
D3 Decision-relevant information	1	Route I: in 18,231 parous women, adding pregnancy complications to established risk factors increased the C-index by 0.004 (95% CI 0.002–0.006), with a net reclassification improvement of 0.02 (95% CI 0.002–0.05), a statistically significant but marginal increment [106]; no analysis of the incremental value of premature menopause was cited.

Domain	Score	Rationale and key evidence
D4 Therapeutic linkage	1	Generic advice: earlier and more frequent risk-factor surveillance; listed among risk modifiers [8], but no intervention of established efficacy in the history-defined group.
D5 Clinical-utility evidence	1	No trial of history-guided prevention with long-term outcomes; support is indirect, from the higher incidence of hypertension after pre-eclampsia [104] and the benefit of treating hypertension once detected [50].
D6 Harms and burden	3	No measurement harm.
D7 Transportability (T) and equity (E)	2	T 2: accuracy of recall varies between settings and groups. E 2: access to postpartum care and follow-up varies.
**17. Polygenic risk score for coronary artery disease** — Adults at intermediate risk or younger adults; statin or lifestyle intensification. Total 11; Tier C.		
D1 Measurement readiness	1	Requires genome-wide genotyping in a specialised laboratory and is not routinely available in primary care. The position of European Society of Cardiology guidelines on routine clinical use [101] is not a measurement property and is not counted here.
D2 Prognostic validity	2	Association with coronary artery disease, with 8% of the population at more than threefold risk [93]; adds information to the pooled cohort equations in a large cohort [94].
D3 Decision-relevant information	1	Route I: small and inconsistent increments in 10-year prediction: C-statistic 0.76 to 0.78 with net reclassification improvement of 4.0% in one cohort [94], no significant improvement in two others [95].
D4 Therapeutic linkage	2	Indirect pathway: larger relative and absolute statin benefit at high genetic risk [96].
D5 Clinical-utility evidence	2	Small randomised trial: disclosure lowered LDL-C through more frequent statin initiation [97]; communication of DNA-based risk did not significantly change behaviour [99].
D6 Harms and burden	2	Psychological impact; false reassurance. In post hoc follow-up of a randomised disclosure trial, fewer participants underwent diagnostic testing for coronary heart disease after disclosure of a risk score that included a PRS (HR 0.51; 95% CI 0.32–0.81) [98].
D7 Transportability (T) and equity (E)	1	T 1: reduced accuracy outside European ancestry [100]. E 2: equitable access not affirmatively shown.
**18. Clonal haematopoiesis of indeterminate potential** — Adults (sequencing); no established preventive action. Total 6; Tier D.		
D1 Measurement readiness	1	Requires sequencing; not standardised for clinical use.
D2 Prognostic validity	2	1.9-fold risk of coronary heart disease in nested case–control analyses of two prospective cohorts and 4.0-fold risk of early-onset myocardial infarction in two retrospective case–control studies [102].
D3 Decision-relevant information	0	Route I: the evidence cited shows associations [102] (scored in D2) but no improvement in risk prediction beyond conventional risk factors. Score changed during revision from 1 to 0, because a score of 1 requires a statistically significant increment.
D4 Therapeutic linkage	0	No proven intervention.
D5 Clinical-utility evidence	0	None.
D6 Harms and burden	1	Haematological incidental findings and anxiety.

Domain	Score	Rationale and key evidence
D7 Transportability (T) and equity (E)	2	T 2: limited validation in diverse populations; age-dependent prevalence. E 2: equity of access not assessable outside research settings. Score changed during revision from 1 to 2, because limited validation corresponds to the level-2 anchor (consistency not affirmatively shown), not to the level-1 anchor (substantially reduced validity).
**19. Large-scale proteomic risk score** — Adults (research platforms); no established preventive action. Total 7; Tier D.		
D1 Measurement readiness	0	Research platforms only.
D2 Prognostic validity	2	Associations with cardiovascular events in large cohorts [103].
D3 Decision-relevant information	1	Route I: in an Icelandic cohort, adding a protein risk score to clinical risk factors (age, sex, statin use, hypertension treatment, T2D, body-mass index and smoking, without cholesterol or BP values) increased the C-index by 0.014 (95% CI 0.002–0.028), a statistically significant but modest increment [103]; a score of 0 would give a total of 6 (Tier D).
D4 Therapeutic linkage	0	No linked intervention.
D5 Clinical-utility evidence	0	None.
D6 Harms and burden	2	No direct harm; uncertain interpretation.
D7 Transportability (T) and equity (E)	2	T 2: limited validation beyond derivation populations. E 2: equity of access not assessable outside research settings. Score changed during revision from 1 to 2, because limited validation corresponds to the level-2 anchor (consistency not affirmatively shown), not to the level-1 anchor (substantially reduced validity).

Scores follow the anchors in Table 1 of the main article (0–3 per domain, ordinal; D6 reverse-scored and restricted to the testing pathway). Each D3 rationale states the route credited (I, incremental information beyond baseline assessment; C, core constituent of guideline-recommended risk equations; S, direct identification of a clinically recognised high-risk state). For D7, transportability (T) and equity (E) are rated separately and the lower rating is the D7 score; under the refined anchors, the D7 scores of use cases 18 and 19 were changed from 1 to 2, and no other D7 score changed. Scores were assigned by the authors as structured judgements. During revision of the manuscript, every score was re-assessed against the refined anchors and the construct definitions in Table S5; six scores were changed (use case 10, D3, from 2 to 1; use case 12, D3, from 1 to 0; use case 15, D2, from 2 to 3; use case 18, D3, from 1 to 0; use cases 18 and 19, D7, from 1 to 2), and several rationales were edited so that each limitation is scored in one domain only. The re-assessment was made with knowledge of the original scores and was neither blinded nor independent. Reference numbers correspond to the reference list of the main article. Statements without a reference are structured judgements about measurement, access or burden. Abbreviations are listed at the end of this document.

Table S2. Quantitative results of studies informing the domain scores.

Ref.	Design and population	Marker or intervention	Principal quantitative findings	Use case (domains)
[7]	Systematic review of prediction models for cardiovascular disease in the general population	Risk prediction models	363 models; 36% externally validated	Context (Section 1)
[17]	Cochrane review of 17 randomised trials (15 with outcome data; 251,891 participants)	General health checks	Total mortality RR 1.00 (0.97–1.03); cardiovascular mortality RR 1.05 (0.94–1.16)	Context (Section 1)
[21]	Individual-participant analysis of 58 prospective cohorts (221,934 participants)	Body-mass index, waist circumference, waist-to-hip ratio	HR per 1 SD after adjustment for systolic BP, diabetes and lipids 1.07, 1.10 and 1.12; C-index change −0.0001, −0.0001 and 0.0008	Context (Section 2)
[22]	Consensus synthesis of more than 200 cohort, Mendelian randomisation and randomised studies (> 2 million participants)	LDL	Consistent, dose-dependent, log-linear relation with ASCVD that increases with duration of exposure	1 (D2); 2 (D4); 3 (D2)
[23]	Individual-participant meta-analysis of 27 statin trials	LDL-C lowering	RR per 1 mmol/L 0.79 (0.77–0.81); 0.62 and 0.69 in the two lowest risk categories; about 11 fewer major vascular events per 1000 over 5 years per 1 mmol/L at a 5-year risk below 10%	1 (D4, D5, D7); 2, 3, 15 (D4)
[43]	Pooled nationally representative surveys from 41 low- and middle-income countries	Statin use among eligible adults	Primary prevention 8.0% (6.9–9.3); secondary prevention 21.9% (20.0–24.0); of the country characteristics examined, per-capita health spending accounted for most of the statistical variation in statin use	1 (D7)
[44]	Mendelian randomisation	LPL and LDLR variants	OR per 10 mg/dL lower apoB 0.771 (0.741–0.802) and 0.773 (0.747–0.801)	2 (D2, D4, D5); 12 (D2, D3)
[45]	Individual-participant analysis of 37 prospective cohorts (165,544 participants without baseline cardiovascular disease)	Apolipoproteins B and A-I, Lp(a), triglycerides and other lipid-related markers added to conventional risk factors	C-index change 0.0006 (0.0002–0.0009) for apoB and apolipoprotein A-I (26 cohorts, 139,581 participants), 0.0016 (0.0009–0.0023) for Lp(a) (24 cohorts, 133,502 participants) and 0.0000 (−0.0001 to 0.0002) for triglycerides (24 cohorts, 133,919 participants); net reclassification improvement < 1% for apoB and apolipoprotein A-I and for Lp(a); 4.1% of intermediate-risk adults reclassified to high risk with Lp(a)	2 (D3); 10 (D3); 12 (D3)

Ref.	Design and population	Marker or intervention	Principal quantitative findings	Use case (domains)
[46]	Cohort (Copenhagen General Population Study), 13,015 statin-treated individuals; median 8 years	Discordance of apoB and non-HDL cholesterol with LDL-C	Discordantly high apoB with LDL-C below the median: myocardial infarction HR 1.49 (1.15–1.92); all-cause mortality HR 1.21 (1.07–1.36)	2 (D3)
[47]	Child–parent screening in primary care (10,095 children)	Cholesterol and FH mutations	Mutation prevalence 1 in 273; 8 people (4 children, 4 parents) identified per 1000 children screened	3 (D4)
[48]	Randomised, placebo-controlled trial, 214 children with FH aged 8–18 years; 2 years	Pravastatin 20–40 mg/d	LDL-C −24.1% vs. +0.3%; carotid intima–media thickness change differed by 0.014 mm (P = 0.02)	3 (D5)
[49]	20-year follow-up of children with FH treated with statins, compared with affected parents	Statin therapy from childhood	LDL-C 237.3 → 160.7 mg/dL; cumulative cardiovascular events by age 39 years 1% vs. 26%	3 (D2, D5)
[50]	Individual-participant meta-analysis of 48 randomised trials (344,716 participants)	BP lowering	Per 5 mmHg systolic BP reduction: HR 0.91 (0.89–0.94) without and 0.89 (0.86–0.92) with prior cardiovascular disease; no heterogeneity by baseline BP	4 (D4, D5, D7); 16 (D5)
[51]	Randomised trial, 9361 high-risk adults without diabetes; median 3.26 years	Intensive vs. standard systolic BP target	Primary outcome HR 0.75 (0.64–0.89); all-cause mortality HR 0.73 (0.60–0.90); more serious adverse events of hypotension, syncope, electrolyte abnormalities and acute kidney injury	4 (D5)
[34]	Cohort, 59,124 patients; median 9.7 years	Clinic vs. 24-h ambulatory BP	All-cause death per SD (upper four fifths of each distribution): 24-h systolic HR 1.41 (1.36–1.47) vs. clinic 1.18 (1.13–1.23); mutually adjusted 1.43 vs. 1.04	4 (D1, D2, D6)
[54]	Pooled analysis of 1201 population-representative studies (104 million participants)	Hypertension treatment and control	2019: treated 47% of women and 38% of men with hypertension; controlled 23% and 18%; wide variation between countries	4 (D7)
[55]	Individual-participant meta-analysis of 102 prospective studies (698,782 participants)	Diabetes; fasting glucose	Diabetes: HR 2.00 (1.83–2.19) for coronary heart disease and 2.27 (1.95–2.65) for ischaemic stroke; fasting glucose did not significantly improve prediction in people without diabetes	5 (D2, D3); 6 (D3)
[56]	Post-trial monitoring of a randomised trial in newly diagnosed T2D (4209 randomised); 10 years	Intensive glucose control (sulfonylurea–insulin; metformin in overweight patients)	Sulfonylurea–insulin: relative reductions in myocardial infarction 15% and death from any cause 13%; metformin: 33% and 27%	5 (D4, D5)

Ref.	Design and population	Marker or intervention	Principal quantitative findings	Use case (domains)
[57]	Cluster-randomised trial, 33 general practices, 20,184 adults at high risk; median 9.6 years	Screening for T2D vs. no screening	All-cause mortality HR 1.06 (0.90–1.25); cardiovascular mortality HR 1.02 (0.75–1.38)	5 (D5)
[35]	Randomised trial, 4304 participants with eGFR 25–75 mL/min/1.73 m² and UACR 200–5000 mg/g	Dapagliflozin	Primary composite 9.2% vs. 14.5%; HR 0.61 (0.51–0.72); consistent with and without diabetes	5 (D4); 7 (D4, D5)
[36]	Prespecified pooled analysis of two randomised trials, 13,026 patients with T2D and CKD; median 3.0 years	Finerenone vs. placebo	Cardiovascular composite HR 0.86 (0.78–0.95); kidney composite HR 0.77 (0.67–0.88)	5 (D4); 7 (D4, D5)
[60]	Randomised trial, 3234 adults with elevated fasting and post-load glucose; mean 2.8 years	Lifestyle intervention; metformin	Incidence of diabetes reduced by 58% (lifestyle) and 31% (metformin)	6 (D4, D5)
[61]	Cluster-randomised trial in impaired glucose tolerance; 30-year follow-up	Lifestyle intervention	Diabetes onset delayed by 3.96 years; cardiovascular events HR 0.74 (0.59–0.92); all-cause death HR 0.74 (0.61–0.89)	6 (D4, D5)
[62]	Long-term follow-up of a randomised trial, 3234 participants with impaired glucose tolerance; median 21 years	Lifestyle intervention; metformin	Major cardiovascular events: metformin vs. placebo HR 1.03 (0.78–1.37); lifestyle vs. placebo HR 1.14 (0.87–1.50)	6 (D4, D5)
[58]	Meta-analysis of 53 prospective cohorts (1,611,339 participants)	Prediabetes	Composite cardiovascular disease RR 1.13–1.30, depending on definition	6 (D2)
[59]	Individual-participant analysis of 73 prospective studies (294,998 participants without known diabetes or cardiovascular disease); median 9.9 years	HbA1c added to conventional risk factors	C-index change 0.0018 (0.0003–0.0033); net reclassification improvement 0.42 (−0.63 to 1.48)	6 (D3)
[63]	Cohort, 3412 older adults (mean age 75.6 years); median 5 years	HbA1c- or fasting glucose-defined prediabetes	HbA1c definition: 9% progressed, 13% regressed, 19% died; impaired fasting glucose: 8%, 44% and 16%	6 (D6)
[64]	Individual-participant meta-analysis	eGFR and UACR	C-statistic increase for cardiovascular mortality 0.0139 (UACR) vs. 0.0065 (eGFR); for HF 0.0196 vs. 0.0109	7 (D2, D3)

Ref.	Design and population	Marker or intervention	Principal quantitative findings	Use case (domains)
[65]	Development and validation of estimating equations	Race-free creatinine and cystatin C equations	Creatinine equation without race: median underestimation of measured GFR by 3.6 mL/min/1.73 m^2 in Black participants and overestimation by 3.9 in non-Black participants; creatinine–cystatin C equations more accurate, with smaller differences between groups	7 (D1, D7)
[66]	Individual-participant meta-analysis, 40 prospective studies (95,617 participants)	NT-proBNP	Top vs. bottom third: RR 2.00 (1.77–2.26) for coronary heart disease, stroke or HF; C-index increase 0.019	8 (D2, D3)
[37]	Randomised trial, 300 patients with T2D and NT-proBNP > 125 pg/mL, free of cardiac disease	Intensified RAS blockade and beta-blockade	Hospitalisation or death due to cardiac disease at 2 years: HR 0.351 (0.127–0.975); all-cause hospitalisation and unplanned cardiovascular hospitalisation or death also reduced ($P < 0.05$ for all)	8 (D1, D5)
[38]	Randomised trial, 1374 people with cardiovascular risk factors in 39 primary care practices; mean 4.2 years	BNP screening (≥ 50 pg/mL) with echocardiography and collaborative care	Left ventricular dysfunction with or without HF 5.3% vs. 8.7%; OR 0.55 (0.37–0.82); emergency hospitalisation for major cardiovascular events (secondary end point) 22.3 vs. 40.4 per 1000 patient-years, incidence rate ratio 0.60 (0.45–0.81); cardiovascular investigations 850 vs. 496 per 1000 patient-years, incidence rate ratio 1.71 (1.61–1.83)	8 (D1, D5, D6)
[4]	Meta-analysis, 36 longitudinal studies (5,802,226 individuals)	MASLD (non-alcoholic fatty liver disease)	Cardiovascular events HR 1.45 (1.31–1.61); higher risk with more advanced liver disease, especially higher fibrosis stage: HR 2.50 (1.68–3.72)	9 (D2)
[68]	Population cohort, 812,073 individuals (FIB-4 available in 126,941); follow-up up to 27 years	FIB-4 and other non-invasive fibrosis scores	Scores identified people at higher risk of incident severe liver disease; accuracy modest in the general population and higher with risk factors for fatty liver disease (AUROC up to 0.83); predictive ability higher in men and decreasing with time	9 (D2, D7)
[69]	Prospective non-randomised evaluation of a two-tier primary-care referral pathway	FIB-4-based pathway	Detection of advanced fibrosis and cirrhosis OR 5.18 (2.97–9.04) vs. standard care; unnecessary referrals reduced by 81% (OR 0.193; 0.111–0.337)	9 (D3, D5)

https://doi.org/10.3390/xxxxx

Ref.	Design and population	Marker or intervention	Principal quantitative findings	Use case (domains)
[25]	Prospective cohort with genetic (instrumental-variable) analysis	Lp(a)	Observational: myocardial infarction HR 2.6 (1.6–4.1) for Lp(a) above the 95th vs. below the 22nd percentile; genetic (instrumental-variable) estimate HR 1.22 (1.09–1.37) per doubling of Lp(a)	10 (D2)
[70]	National biobank cohort; median 11.2 years	Lp(a)	22,401 incident ASCVD events; HR 1.11 (1.10–1.12) per 50 nmol/L; linear relation	10 (D2)
[72]	Mendelian randomisation	LPA variants	Lp(a) reduction of 101.5 mg/dL (71.0–137.0) equivalent to a 38.67 mg/dL LDL-C reduction for coronary risk	10 (D4, D5)
[73]	Individual-participant meta-analysis, 52 prospective studies (246,669 participants; CRP measured in 166,596 participants from 38 cohorts)	CRP, fibrinogen	C-index increase 0.0039 (CRP); about one additional event prevented over 10 years per 400–500 people screened for CRP or fibrinogen	11 (D2, D3)
[24]	Mendelian randomisation, individual-participant data	CRP	Genetic RR per SD 1.00 (0.90–1.13) vs. observational 1.33 (1.23–1.43)	11 (D2)
[18]	Randomised trial, 17,802 people with LDL-C < 130 mg/dL and hs-CRP ≥ 2 mg/L	Rosuvastatin	Primary endpoint 0.77 vs. 1.36 per 100 person-years; HR 0.56 (0.46–0.69)	11 (D4, D5)
[74]	Individual-participant meta-analysis of 68 prospective studies (302,430 participants)	Major lipids and apolipoproteins	Coronary heart disease HR per 1 SD higher log triglyceride 0.99 (0.94–1.05) after adjustment for HDL and non-HDL cholesterol; in the 22 studies (91,307 participants) with apolipoprotein measurements, apoB and non-HDL cholesterol had associations of very similar shape and magnitude, and the HR per 1 SD of the apoB/apolipoprotein A-I ratio was 1.49 (1.39–1.60)	2 (D2); 12 (D2, D3)
[75]	Randomised trial in statin-treated patients with elevated triglycerides	Icosapent ethyl	Primary endpoint HR 0.75 (0.68–0.83); primary-prevention cohort HR 0.88 (0.70–1.10); hospitalisation for atrial fibrillation or flutter 3.1% vs. 2.1%	12 (D4, D5)
[76]	Randomised trial, 13,078 high-risk patients	Omega-3 fatty acids vs. corn oil	Primary endpoint 12.0% vs. 12.2%; HR 0.99 (0.90–1.09)	12 (D4, D5)
[77]	Randomised trial, 10,497 patients with T2D, triglycerides 200–499 mg/dL and HDL-C ≤ 40 mg/dL; median 3.4 years	Pemafibrate	Triglycerides −26.2%, apoB +4.8%; HR 1.03 (0.91–1.15)	12 (D4, D5)

Ref.	Design and population	Marker or intervention	Principal quantitative findings	Use case (domains)
[26]	Cohort (MESA), 6722 participants from four ethnic groups	CAC	Any coronary event: adjusted HR 9.67 for scores > 300 vs. 0; per doubling of the score, 15–35% higher risk of a major coronary event	13 (D2, D7)
[40]	Cohort (MESA), statin candidates	CAC	44% had a score of 0; ASCVD rate 4.2 per 1000 person-years with a score of 0	13 (D3)
[39]	Cohort (MESA), 1330 intermediate-risk participants without diabetes; median 7.6 years	CAC, ABI, hs-CRP, family history, carotid intima–media thickness, flow-mediated dilation	CAC gave the largest improvement in discrimination and reclassification among the markers compared; net reclassification improvement for coronary heart disease 0.659 (CAC), 0.160 (family history), 0.102 (carotid intima–media thickness), 0.079 (hs-CRP), 0.036 (ABI) and 0.024 (flow-mediated dilation)	11, 13, 14, 15 (D3)
[41]	Randomised trial, 2137 volunteers	CAC scanning vs. no scanning	Favourable changes in systolic BP, LDL-C and, in those with increased abdominal girth, waist circumference; similar downstream testing and costs	13 (D5, D6)
[42]	Randomised trial, 46,611 men aged 65–74 years; median 5.6 years	Invitation to screening including CAC and ABI vs. usual care	All-cause death HR 0.95 (0.90–1.00); stroke HR 0.93 (0.86–0.99)	13, 15 (D5)
[79]	Cohort (MESA), 3442 participants in six centres	CAC scanning	Mean effective radiation dose 1.05 mSv per scan (median 0.95 mSv)	13 (D6)
[81]	Meta-analysis of 11 population-based studies (54,336 participants)	Carotid plaque vs. carotid intima–media thickness	Prediction of myocardial infarction: area under the curve 0.64 vs. 0.61; relative diagnostic OR 1.35 (1.1–1.82)	14 (D2)
[82]	Cohort (MESA), 6779 participants without cardiovascular disease; mean 9.5 years	Carotid plaque presence, carotid intima–media thickness and CAC presence added to traditional risk factors	C-statistic for cardiovascular disease 0.756 with risk factors, 0.760 with carotid plaque presence ($P < 0.05$) and 0.776 with CAC presence ($P < 0.001$)	14 (D3)
[83]	Cohort (ARIC), 13,145 participants	Carotid intima–media thickness and plaque added to traditional risk factors	Area under the curve 0.742 → 0.755 (95% CI of the difference 0.008–0.017); net reclassification improvement 9.9%; about 23% of participants reclassified	14 (D3)
[84]	Cohort (BioImage), 5808 asymptomatic adults (mean age 69 years); median 2.7 years	Three-dimensional carotid plaque burden and CAC	Net reclassification improvement 0.23 (carotid plaque burden) and 0.25 (CAC); major adverse cardiac events HR 2.36 (1.13–4.92) for the highest plaque-burden tertile vs. no plaque	14 (D3)

Ref.	Design and population	Marker or intervention	Principal quantitative findings	Use case (domains)
[85]	Pragmatic randomised trial, 3532 participants (1749 intervention, 1783 control)	Pictorial information on carotid ultrasound findings (plaque, vascular age) with nurse telephone follow-up	Primary outcomes at 1 year (calculated risk scores): Framingham risk score difference 1.07 (0.11–2.03) and SCORE difference 0.16 (0.02–0.30) in favour of the intervention	14 (D5)
[86]	Same randomised trial, 3-year report (multilevel models adjusted for sex and education)	Pictorial information on carotid ultrasound findings	LDL-C lower in the intervention group by 0.17 mmol/L (0.11–0.24) at 1 year and 0.23 mmol/L (0.16–0.30) at 3 years	14 (D5)
[87]	Individual-participant meta-analysis, 16 cohorts	ABI ≤ 0.90	10-year cardiovascular mortality, ABI ≤ 0.90 vs. 1.11–1.40: HR 4.2 (3.3–5.4) in men and 3.5 (2.4–5.1) in women (2.9 [2.3–3.7] and 3.0 [2.0–4.4] after adjustment for the Framingham risk score); risk category changed in 19% of men and 36% of women	15 (D2, D3, D4)
[90]	Randomised trial, 3350 adults with ABI ≤ 0.95 identified by screening 28,980; mean 8.2 years	Aspirin vs. placebo	Primary vascular events HR 1.03 (0.84–1.27); major haemorrhage requiring admission HR 1.71 (0.99–2.97)	15 (D4, D5)
[91]	Randomised trial, 50,156 men aged 65–74 years; median 4.4 years	Screening for abdominal aortic aneurysm, peripheral artery disease and hypertension vs. no screening	All-cause mortality HR 0.93 (0.88–0.98)	15 (D5)
[104]	Meta-analysis of cohort studies	Pre-eclampsia	Hypertension RR 3.70 (2.70–5.05); ischaemic heart disease RR 2.16 (1.86–2.52)	16 (D2, D5)
[105]	Cohort (UK Biobank), 144,260 postmenopausal women; median 7 years	Natural and surgical menopause before 40 years	Composite cardiovascular disease HR 1.36 (1.19–1.56) and 1.87 (1.36–2.58), adjusted for conventional risk factors and menopausal hormone therapy	16 (D2)
[106]	Cohort (HUNT, Norway), 18,231 parous women aged ≥ 40 years without cardiovascular disease; median 8.2 years	History of pregnancy complications added to an established risk model (NORRISK 2)	C-index difference 0.004 (0.002–0.006); net reclassification improvement 0.02 (0.002–0.05); pre-eclampsia HR 1.60 (1.16–2.17) after adjustment for established risk factors	16 (D3)
[93]	Derivation and validation of genome-wide polygenic scores	CAD PRS	8.0% of the population with more than threefold risk, 20 times the frequency of monogenic mutations conferring comparable risk	17 (D2)

Ref.	Design and population	Marker or intervention	Principal quantitative findings	Use case (domains)
[94]	Population cohort	PRS added to pooled cohort equations	C-statistic 0.76 → 0.78; net reclassification improvement 4.0% (3.1–4.9)	17 (D2, D3)
[97]	Randomised trial, 203 participants	Disclosure of a genetic risk score	LDL-C at 6 months 96.5 vs. 105.9 mg/dL; statin initiation 39% vs. 22%	17 (D5)
[99]	Meta-analysis of randomised and quasi-randomised trials	Communication of DNA-based risk estimates	Smoking cessation OR 0.92 (0.63–1.35); little or no effect on diet or physical activity	17 (D5)
[102]	Whole-exome sequencing in four case–control studies (4726 participants with coronary heart disease, 3529 controls): nested case–control analyses in two prospective cohorts and two retrospective case–control studies of early-onset myocardial infarction	CHIP	Coronary heart disease 1.9-fold (1.4–2.7) in the prospective cohorts; early-onset myocardial infarction 4.0-fold (2.4–6.7) in the retrospective studies	18 (D2, D3)
[103]	Cohort (Iceland), 13,540 adults aged 40–75 years without ASCVD (test set 4018); median 15.8 years	Protein risk score derived from 4963 plasma proteins	HR 1.93 (1.75–2.13) per SD; C-index change 0.014 (0.002–0.028) when added to clinical risk factors	19 (D2, D3)

Values are those reported in the cited publications (95% confidence intervals in parentheses where reported), with the endpoint definitions of the original publications. Reference numbers correspond to the reference list of the main article. The table is a structured summary for the reader and does not represent a pooled analysis. Abbreviations are listed at the end of this document.

https://doi.org/10.3390/xxxxx

Table S3. Self-assessment against the Scale for the Assessment of Narrative Review Articles (SANRA).

SANRA item	Score	Justification
1. Justification of the article's importance for the readership	2	Section 1 sets out the burden of cardiometabolic disease, the growth in available markers and the gap between prediction and action in primary care.
2. Statement of concrete aims or formulation of questions	2	Four objectives are stated at the end of Section 1 and are addressed in Sections 3, 4, 5 and 6, respectively.
3. Description of the literature search	1	Section 2 describes the database consulted, the concept groups considered, citation tracking, the types of evidence prioritised, general inclusion and exclusion principles, the handling of conflicting evidence and the logic of use-case selection, consistent with a narrative review.
4. Referencing	2	Key statements are supported by 111 references, predominantly primary trials, individual-participant meta-analyses, Mendelian randomisation studies and current guidelines.
5. Scientific reasoning	2	The evidence hierarchy is considered explicitly, established findings are distinguished from emerging ones, and conflicting evidence is examined for each marker class (Sections 5.1–5.5).
6. Appropriate presentation of relevant endpoint data	2	Effect sizes with 95% confidence intervals and the endpoint definitions of the original publications are reported for principal outcomes in Section 5 and Table S2.
Total	11/12	Total 11 of 12. The principal limitation is item 3, which reflects the narrative design.

Each item is scored from 0 (low standard) to 2 (high standard) [20]. The self-assessment was made by the authors as a reporting aid; it concerns the reporting of the narrative review and does not validate the proposed framework. Reference numbers correspond to the reference list of the main article.

Table S4A. Sensitivity of tier assignments to single-domain score perturbations.

No.	Use case	Total	Tier	Perturbations (n)	Tier changes (n)	Perturbations that change the tier
1	LDL-C / non-HDL-C	20	A	8	1	D4−1→B
2	Apolipoprotein B	16	B	11	1	D5+1→A
3	Familial hypercholesterolaemia case-finding	17	A	11	7	D1−1→B; D2−1→B; D3−1→B; D4−1→B; D5−1→B; D6−1→B; D7−1→B
4	Blood pressure (office, with out-of-office confirmation)	19	A	9	1	D4−1→B
5	HbA1c or fasting glucose: type 2 diabetes	18	A	10	2	D4−1→B; D5−1→B
6	HbA1c or fasting glucose: prediabetes	14	B	13	1	D4−1→C
7	eGFR plus UACR	19	A	9	1	D4−1→B

No.	Use case	Total	Tier	Perturbations (n)	Tier changes (n)	Perturbations that change the tier
8	Natriuretic peptides (BNP or NT-proBNP)	15	B	13	1	D4−1→C
9	FIB-4 index	14	B	13	1	D4−1→C
10	Lipoprotein(a)	13	B	13	7	D1−1→C; D2−1→C; D3−1→C; D4−1→C; D5−1→C; D6−1→C; D7−1→C
11	hs-CRP	14	B	13	1	D4−1→C
12	Triglycerides	10	C	12	0	None
13	Coronary artery calcium score	15	B	12	1	D4−1→C
14	Carotid plaque ultrasound	13	B	14	7	D1−1→C; D2−1→C; D3−1→C; D4−1→C; D5−1→C; D6−1→C; D7−1→C
15	Ankle–brachial index	13	B	13	7	D1−1→C; D2−1→C; D3−1→C; D4−1→C; D5−1→C; D6−1→C; D7−1→C
16	Pregnancy complications and premature menopause	13	C	12	1	D4+1→B
17	Polygenic risk score for coronary artery disease	11	C	14	0	None
18	Clonal haematopoiesis of indeterminate potential	6	D	11	0	None
19	Large-scale proteomic risk score	7	D	11	0	None
	**All use cases**			**222**	**40 (18.0%)**	**Changes by domain: D1 4, D2 4, D3 4, D4 14, D5 6, D6 4, D7 4**

Each domain score of each use case was changed by −1 and by +1 within the 0–3 range, and the tier was recomputed with the rules in Table 1 of the main article. Perturbations that change the tier are listed as domain, direction and resulting tier. Lowering all tier thresholds by one point changed no tier; raising them by one point changed four (familial hypercholesterolaemia case-finding, A→B; lipoprotein(a), carotid plaque ultrasound and ankle–brachial index, B→C); applying the total-score thresholds without gating rules changed one tier (pregnancy complications and premature menopause, C→B). Results reflect the scores after the re-assessment performed during revision (use case 10, D3 = 1; use case 12, D3 = 0; use case 15, D2 = 3; use case 18, D3 = 0 and D7 = 2; use case 19, D7 = 2); additional analyses are reported in Table S4B. Abbreviations are listed at the end of this document.

Table S4B. Additional sensitivity analyses of tier assignments performed during revision of the manuscript.

No.	Use case (total, tier)	Two-domain ±1: tier changes/combinations	Uniform threshold shifts that change the tier	Single-domain ±1 with thresholds −1, 0 or +1: changes/scenarios	Weighting scenarios that change the tier	Perturbations that change the tier (summary)
1	LDL-C / non-HDL-C (20, A)	7/27	None	3/24	None	One-point reduction in D4
2	Apolipoprotein B (16, B)	3/51	None	2/33	None	One-point increase in D5
3	Familial hypercholesterolaemia case-finding (17, A)	28/51	+1, +2 (→B)	16/33	D4/D5 once; without D2, D3 or D4 (→B)	Any one-point reduction; uniform threshold shift +1 or +2; alternative weighting; D3 capping
4	Blood pressure (19, A)	8/34	None	3/27	None	One-point reduction in D4
5	HbA1c or fasting glucose: type 2 diabetes (18, A)	26/42	+2 (→B)	11/30	None	One-point reduction in D4 or D5; uniform threshold shift +2
6	HbA1c or fasting glucose: prediabetes (14, B)	26/72	+2 (→C)	9/39	Without D1 (→C)	One-point reduction in D4; uniform threshold shift +2; removal of D1 from the total
7	eGFR plus UACR (19, A)	8/34	None	3/27	None	One-point reduction in D4
8	Natriuretic peptides (15, B)	16/72	None	4/39	None	One-point reduction in D4
9	FIB-4 index (14, B)	26/72	+2 (→C)	9/39	Without D1 (→C)	One-point reduction in D4; uniform threshold shift +2; removal of D1 from the total

No.	Use case (total, tier)	Two-domain ±1: tier changes/combinations	Uniform threshold shifts that change the tier	Single-domain ±1 with thresholds −1, 0 or +1: changes/scenarios	Weighting scenarios that change the tier	Perturbations that change the tier (summary)
10	Lipoprotein(a) (13, B)	26/72	+1, +2 (→C)	15/39	D1/D6/D7 once; without D1, D2, D4, D6 or D7 (→C)	Any one-point reduction; uniform threshold shift +1 or +2; alternative weighting
11	hs-CRP (14, B)	26/72	+2 (→C)	9/39	Without D1 (→C)	One-point reduction in D4; uniform threshold shift +2; removal of D1 from the total
12	Triglycerides (10, C)	15/61	+2 (→D)	6/36	D1/D6/D7 once (→D)	Uniform threshold shift +2; alternative weighting
13	Coronary artery calcium score (15, B)	14/61	None	4/36	None	One-point reduction in D4
14	Carotid plaque ultrasound (13, B)	27/84	+1, +2 (→C)	15/42	D4/D5 once; D1/D6/D7 once; without D1, D2, D4, D5, D6 or D7 (→C)	Any one-point reduction; uniform threshold shift +1 or +2; alternative weighting
15	Ankle–brachial index (13, B)	26/72	+1, +2 (→C)	15/39	D1/D6/D7 once; without D1, D2, D4, D6 or D7 (→C)	Any one-point reduction; uniform threshold shift +1 or +2; alternative weighting
16	Pregnancy complications and premature menopause (13, C)	10/61	None	3/36	None	One-point increase in D4; removal of the gating rules
17	Polygenic risk score for coronary artery disease (11, C)	21/84	−2 (→B)	7/42	None	Uniform threshold shift −2

No.	Use case (total, tier)	Two-domain ±1: tier changes/ combinations	Uniform threshold shifts that change the tier	Single-domain ±1 with thresholds −1, 0 or +1: changes/ scenarios	Weighting scenarios that change the tier	Perturbations that change the tier (summary)
18	Clonal haematopoiesis of indeterminate potential (6, D)	0/51	None	0/33	None	None of the perturbations examined
19	Large-scale proteomic risk score (7, D)	21/51	−2 (→C)	7/33	D4/D5 once; without D1, D4 or D5 (→C)	Uniform threshold shift −2; alternative weighting
	All use cases	334/1124 (29.7%)	−1: none; +1: 4; −2: 2; +2: 9	141/666 (21.2%)	D4/D5 once: 3; D1/D6/D7 once: 4; leave-one-domain-out: 1–7	Single-domain: any one-point reduction 4, D4 or D5 only 11, none 4; no change in any analysis 1

Analyses performed during revision of the manuscript on the scores in Table 2 of the main article; the one-point uniform threshold shifts and the removal of the gating rules repeat analyses reported in the original version, and the other analyses were added during revision. Two-domain: every unordered pair of distinct domains (21 pairs) changed by ±1 each in all four sign combinations, keeping only combinations within the 0–3 range. Thresholds: all cut-points (8/9, 12/13 and 16/17) shifted together by −2, −1, +1 or +2 points; shifting only the Tier A cut-point by +1 changed use case 3, shifting only the Tier B cut-point by +1 changed use cases 10, 14 and 15, and the other single cut-point shifts of one point changed no tier. Combined: each single-domain perturbation of Table S4A (222) evaluated with thresholds shifted by −1, 0 and +1 point (666 scenarios); a change is a difference from the published tier, so it includes changes caused by the threshold shift alone. Weighting: D4/D5 once, D4 and D5 each weighted 0.5 (maximum total 18); D1/D6/D7 once, D1, D6 and D7 each weighted one third (maximum 15); without Dx, domain x removed from the total (maximum 18). In these scenarios, the inclusive cut-points (≤ 8, ≥ 13 and ≥ 17) were multiplied by the ratio of the maximum attainable total to 21, without rounding (maximum 18: Tier D ≤ 6.86, Tier B ≥ 11.14, Tier A ≥ 14.57; maximum 15: Tier D ≤ 5.71, Tier B ≥ 9.29, Tier A ≥ 12.14), and the gating rules were retained; weights were defined by the suspected overlaps between domains and were not adjusted to reproduce existing assignments. Some weighted totals lie within 0.1 point of a rescaled cut-point (use case 3: 14.50 with D4/D5 once; use cases 6, 9 and 11: 9.33 with D1/D6/D7 once), so these results depend on the rescaling convention. Capping routes C and S of D3 at 2 changed only use case 3 (A→B), and removing the gating rules changed only use case 16 (C→B). Last column: the perturbations that change the tier, summarising Table S4A (single-domain changes) and the threshold, weighting and rule-change analyses in this table; alternative weighting covers the overlap weightings and the removal of single domains listed in the sixth column. These summaries were compiled during revision from the results; they are descriptive, were not prespecified and are not a classification of validity. The two-domain results are reported as counts only, because every two-domain change either involves D4 or D5 or is equivalent to a two-point uniform threshold shift: a same-direction change in two domains other than D4 and D5 changes the tier exactly when the corresponding two-point shift

does, and an opposite-direction change in such domains cannot change it. Stability under these perturbations does not constitute validation, because all were applied to the same author-assigned scores and thresholds, and the proportions of changed scenarios are not probabilities of misclassification.

Table S5. Construct definitions of the seven domains and rules preventing double counting.

Domain	What the domain measures	Evidence scored in this domain	Evidence not scored here (domain where it is scored)	Rule preventing double counting
D1 Measurement readiness	Analytical and practical readiness of the measurement in primary care	Analytical validity; standardisation of assays and units; established thresholds; availability in primary care, need for referral or specialised equipment; turnaround; cost of the test itself	Prognostic strength (D2); harms of confirmation and downstream testing (D6); performance that differs between population groups and inequity of access or uptake (D7)	Test cost and availability are scored only here; a measurement limitation confined to particular groups is scored under transportability (D7)
D2 Prognostic validity	Strength, consistency, gradient and independence of the association with hard cardiometabolic outcomes	Prospective cohorts and individual-participant meta-analyses; genetic association studies as support for consistency	Incremental or reclassification metrics (D3); measurement reliability (D1); differences in validity between groups (D7); efficacy of treatment (D4)	Measurement limitations are not used to lower D2; group-specific limitations are scored in D7
D3 Decision-relevant information	Whether the result informs the decision at hand, by route I, C or S	Route I: change in discrimination, reclassification near the decision threshold, de-risking or rule-out; route C: core constituent of guideline-recommended risk equations; route S: clinically recognised high-risk state defined by the marker	Strength of association as such (D2); efficacy of the linked intervention (D4); guideline recommendations to measure the marker, which are not in themselves evidence of decision-relevant information	The route is recorded for every score; optional predictors in extended equations are credited only through route I; routes C and S are examined in a sensitivity analysis capped at 2
D4 Therapeutic linkage	Existence and directness of a credible pathway from the result to an intervention of established efficacy (target or triage route)	Causal evidence for target-route markers; efficacy of the linked intervention in treatment trials, in any relevant population; whether the result defines a condition with effective treatment	Evidence that acting on the result benefits the people it selects (D5); adverse effects of treatment (reflected in net efficacy, not in D6)	A treatment trial supports D4 as evidence of efficacy; it contributes to D5 only if it enrolled people defined by the marker or its condition, or directed therapy at the marker

Domain	What the domain measures	Evidence scored in this domain	Evidence not scored here (domain where it is scored)	Rule preventing double counting
D5 Clinical-utility evidence	Evidence that acting on the result, in people defined by the marker or its condition, improves outcomes	Randomised trials of marker-guided versus usual care; trials of testing strategies; treatment trials in marker-defined populations; randomised evidence on validated intermediate outcomes	Efficacy of the intervention in populations not selected by the marker (D4); calculated risk scores, which are not counted as outcomes	Reading rules: conflicting randomised evidence at a level does not support that level; consistent evidence of no benefit scores 0; a null trial of the testing strategy caps D5 at 2
D6 Harms and burden of the testing pathway	Harms and burdens of measurement, confirmation and downstream investigation for the person tested	Radiation; invasive follow-up; false-positive results; incidental findings; overdiagnosis; labelling; unnecessary downstream testing; burden of confirmation	Test cost and availability (D1); inequity of access (D7); adverse effects of treatment (D4 and D5)	Each harm is counted once, here; cost and access problems are not counted here
D7 Transportability (T) and equity (E)	T: whether measurement, thresholds and prognostic performance hold across sex, age, ancestry, socioeconomic groups and settings; E: equitable access, uptake and follow-through and the risk of widening disparities	T: subgroup validation, ancestry-specific performance, dependence of assays or thresholds on patient characteristics; E: data on uptake, treatment and follow-through by group	Overall test cost or availability (D1); harms (D6); overall prognostic strength (D2)	T and E are rated separately on the Table 1 anchors and the lower rating is scored; an access problem is scored under E only when it concerns its distribution across groups or the risk of widening disparities

Construct definitions made explicit during revision of the manuscript and applied in the re-assessment of all scores reported in Table S1. Anchors and tier rules are given in Table 1 of the main article. For target-route markers (e.g., LDL-C, blood pressure), treatment is directed at the measured value, so the same trials can inform D4 and D5 for different properties; the resulting correlation is examined in Table S4B (D4/D5 counted once). Abbreviations are listed at the end of this document.

Abbreviations

ABI	ankle–brachial index
apoB	apolipoprotein B
ARIC	Atherosclerosis Risk in Communities
ASCVD	atherosclerotic cardiovascular disease
AUROC	area under the receiver-operating characteristic curve
BNP	B-type natriuretic peptide
BP	blood pressure
CAC	coronary artery calcium
CAD	coronary artery disease
CHIP	clonal haematopoiesis of indeterminate potential
CI	confidence interval
CKD	chronic kidney disease
CKM	cardiovascular–kidney–metabolic
CRP	C-reactive protein
eGFR	estimated glomerular filtration rate
FH	familial hypercholesterolaemia
FIB-4	Fibrosis-4 index

GFR	glomerular filtration rate
HbA1c	glycated haemoglobin
HDL	high-density lipoprotein
HDL-C	high-density lipoprotein cholesterol
HF	heart failure
HR	hazard ratio
hs-CRP	high-sensitivity C-reactive protein
LDL	low-density lipoprotein
LDL-C	low-density lipoprotein cholesterol
LDLR	gene encoding the low-density lipoprotein receptor
Lp(a)	lipoprotein(a)
LPA	gene encoding apolipoprotein(a)
LPL	gene encoding lipoprotein lipase
MASLD	metabolic dysfunction-associated steatotic liver disease
MESA	Multi-Ethnic Study of Atherosclerosis
non-HDL(-C)	non-high-density lipoprotein (cholesterol)
NT-proBNP	N-terminal pro-B-type natriuretic peptide
OR	odds ratio
PAD	peripheral artery disease
PREVENT	Predicting Risk of cardiovascular disease EVENTs
PRS	polygenic risk score(s)
RAS	renin–angiotensin system
RR	risk ratio or rate ratio
SANRA	Scale for the Assessment of Narrative Review Articles
SCORE	Systematic Coronary Risk Evaluation
SCORE2	Systematic Coronary Risk Evaluation 2
SD	standard deviation
SGLT2	sodium–glucose cotransporter 2
T2D	type 2 diabetes
UACR	urinary albumin-to-creatinine ratio
